# Kabirian-based optinalysis: A conceptually grounded framework for symmetry/asymmetry, similarity/dissimilarity and identity/unidentity estimations in mathematical structures and biological sequences

**DOI:** 10.1016/j.mex.2023.102400

**Published:** 2023-10-01

**Authors:** Kabir Bindawa Abdullahi

**Affiliations:** Department of Biology, Faculty of Natural and Applied Sciences, Umaru Musa Yar'adua University, P.M.B., Katsina, Katsina State 2218, Nigeria

**Keywords:** Kabirian-based Optinalysis, Autoreflective pair, Isoreflective pair, Optiscale, Pairwise sequence analysis, Position-specific match/mismatch/deletion, Character-specific mismatch, Sensitivity

## Abstract

This paper introduces “Kabirian-based optinalysis (KBO),” a pioneering framework that addresses the longstanding challenges in estimating symmetry/asymmetry, similarity/dissimilarity, and identity/unidentity within mathematical structures and biological sequences. The existing methods often lack a strong theoretical foundation, leading to inconsistencies and limitations. Kabirian-based optinalysis draws inspiration from isomorphism and automorphism, providing a theoretically grounded framework that unifies estimation methodologies. It introduces the concept of optiscale, autoreflective pairing, isoreflective pairing, and others ensuring invariance and robustness under various mathematical transformations and establishing functional bijectivity for isomorphic or automorphic structures. This not only overcomes previous limitations but also offers precise and interpretable estimations. Additionally, the framework introduces “geometrical pairwise analysis” to improve sensitivity to position-specific and character-specific variations in biological sequences. This novel approach enhances the accuracy of sequence similarity assessments, surpassing the constraints of conventional methods. The novelty of this work extends beyond mathematics and biology, impacting diverse fields such as computer science, data analysis, pattern recognition, and evolutionary biology. Kabirian-based optinalysis presents a holistic and theoretically grounded solution that has the potential to revolutionize the analysis of complex structures and sequences, opening new horizons for interdisciplinary research.

• Inspired by automorphism and isomorphism, Kabirian-based optinalysis introduces a new paradigm-shifting and unified approach to estimations in mathematical structures and biological sequences with a solid conceptual and theoretical foundation.
• The GPA method enhances pairwise sequence similarity estimation by being sensitive to position-specific and character-specific variations and providing a comprehensive characterization of these features.

## Graphical abstract

The graphical abstract for Kabirian-based automorphic and isomorphic optinalysis was provided as a single image in the attached files of this submission.

**Figure:**
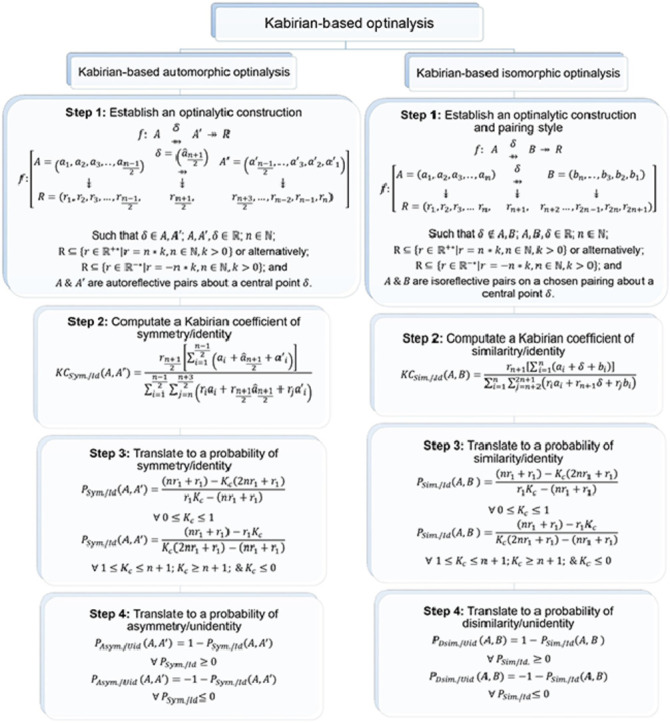
Graphical abstract for Kabirian-based automorphic and isomorphic optinalysis.

Specifications table

**Table:** 

Subject area:	Mathematics and Statistics
More specific subject area:	Numerical Analysis and Biological Sequence Analysis
Name of your method:	Kabirian-based Optinalysis
Name and reference of original method:	Not applicable
Resource availability:	Get the Python codes for Kabirian-based automorphic and isomorphic optinalysis, as well as geometrical pairwise analysis of biological sequences via these links: 1. Abdullahi, K. B. (2023). Python Codes for Kabirian-based Automorphic and Isomorphic Optinalysis. Mendeley Data, V2, doi:10.17632/gnrcj8s7fp.2 (https://data.mendeley.com/datasets/gnrcj8s7fp/2) 2. Abdullahi, K.B. (2023). Python Code for Geometrical Pairwise Analysis of Biological Sequences Following Kabirian-based Isomorphic Optinalysis. Mendeley Data, V2, doi:10.17632/tnwpt54jnb.2 (https://data.mendeley.com/datasets/tnwpt54jnb/2)

## Method details

### Terminology and definitions

In this section, a set of definitions were presented that are unique to the research. These definitions are introduced for the first time or conceptually modified and form the basis of the methodological novelties of the research.

Definition 1 Theoretical ordering

Theoretical ordering refers to theory-based, or concept-based structuring or arrangement of terms and items. For instance, the arrangement of real numbers in ascending or descending order is theory-based.

Definition 2 Empirical ordering

Conceptual ordering refers to the structuring or arrangement of terms and items based on empirical observations, measurable data, or practical considerations, as opposed to theoretical principles. For instance, the arrangement of RNA, DNA, and amino acid sequences relies on empirical evidence obtained through experimental techniques in molecular biology and genetics.

Definition 3 Autoreflective pair

Autoreflective pair describes a concatenated mirror isomorphism of a mathematical structure to itself about a center. Let $A^{\prime }=\left(a{{}^{\prime }}_{1},a{{}^{\prime }}_{2},\;a{{}^{\prime }}_{3},\ldots ,a{{}^{\prime }}_{n}\right)$ be a mirror image to $A=(a_{1},a_{2},\;a_{3},\ldots ,a_{n})$ of a mathematical structure. Then, the autoreflective pair is represented as:

Such that $\delta \in A,\;A^{\prime }$; $\delta ,A,\;A{}^{\prime }\in R$.

Remark 1 The standard notation to represent mirror isomorphism,$A≅A^{\prime }$ or $A\to A^{\prime }$, is modified as  to emphasize a center δ as an important term, as well as the concatenation of the pair. Mirror isomorphism does not involve concatenating the geometric and its mirrored object but rather establishes a duality and correspondence between the two objects, allowing for a study of their interconnected properties.

Definition 4 Isoreflective pair

An isoreflective pair describes a concatenated mirror isomorphism between two mathematical structures about a center. Let $A=(a_{1},a_{2},\;a_{3},\ldots ,a_{n})$ and $B=(b_{1},b_{2},\;b_{3},\ldots ,b_{n})$ two mathematical structures. Then, the isoreflective pair is represented as:

Such that δ∉A,B; δ,A,B∈R.

Remark 2 The standard notation to represent mirror isomorphism,A≅B or A→B, is modified as  to emphasize a center δ as an important term, as well as the concatenation of the pair.

Definition 5 Head-to-head reflection or pairing

A reflection or pairing of the isoreflective pair is head-to-head if the first terms (elements) of the isoreflective pair are maximally distant from the central connection point. Let $A=(a_{1},a_{2},\;a_{3},\ldots ,a_{n})$ and $B=(b_{1},b_{2},\;b_{3},\ldots ,b_{n})$ be two mathematical structures about a center δ. Then, head-to-head isoreflective pairing is represented as:

Such that δ∉A,B; δ,A,B∈R.

Definition 6 Tail-to-tail reflection or pairing

A reflection or pairing of an isoreflective pair is head-to-head if the first terms (elements) of the isoreflective pair are minimally distant (i.e., positioned at their closest proximity) from the central connection point. Let $A=(a_{1},a_{2},\;a_{3},\ldots ,a_{n})$ and $B=(b_{1},b_{2},\;b_{3},\ldots ,b_{n})$ two mathematical structures about a center δ. Then, tail-to-tail isoreflective pairing is represented as:

Such that δ∉A,B; δ,A,B∈R.

Definition 7 Pericentral rotation

Pericentral rotation refers to the turning of all the members of two mathematical structures of an isoreflective pair through 180° around the pericentres.

A pericentre is the median point of each mathematical structure. Pericentral rotation is similar to alternate reflection (i.e., from the head-to-head to tail-to-tail reflection or otherwise). An alternate reflection is the alternative form of reflection between isoreflective pairs. Alternate reflection can be used, in some cases, to distinguish between two similar structures but not identical to each other.

Definition 8 Central rotation

Central rotation refers to the turning of all the members of two mathematical structures of an isoreflective pair through 180° around the central point. Central rotation is similar to inversion transformation.

Let  be a tail-to-tail isoreflective pair of two mathematical structures around a central (δ), such that δ∉A,B; δ,A,B∈R

Then, its central rotation or inversion becomes :

Definition 9 Optiscale

Optiscale (denoted as R) is a subset of either the positive real numbers (excluding zero) or the negative real numbers (excluding zero). The optiscale consists of numbers that can be represented as multiples of a positive constant k, where k represents the uniform interval between the numbers in the scale. The notation used to represent the optiscale is as follows:

For the subset of positive real numbers:

$$R\subseteq \left\{r\in R^{+*}|r=n*k,\;n\in N,\;k>0\right\}$$

For the subset of negative real numbers:

$$R\subseteq \left\{r\in R^{-*}|r=-n*k,\;n\in N,\;k>0\right\}$$

In both cases, $R^{+*}$ represents the set of positive real numbers (excluding zero),$R^{-*}$ represents the set of negative real numbers(excluding zero), N represents the set of natural numbers (positive integers), and n is a natural number that acts as a multiplier for k. The optiscale includes all numbers that can be obtained by multiplying the positive constant k by a natural number n.

Because the elements of the optiscale belong to the subset of non-zero real numbers, the complexity of the arithmetic operations in optinalysis can be minimized especially when dealing with numerical values that are decimally low or high.

Definition 10 Optinalysis

Optinalysis is a function that autoreflectively or isoreflectively compares the symmetry/asymmetry, similarity/dissimilarity, and identity/unidentity within one or between two mathematical structures as a mirror-like (optic-like) reflection of each other about a central point. In other words, it is a function that numerically compares isoreflective or autoreflective pairs of mathematical structures.

**Fig. 1:**
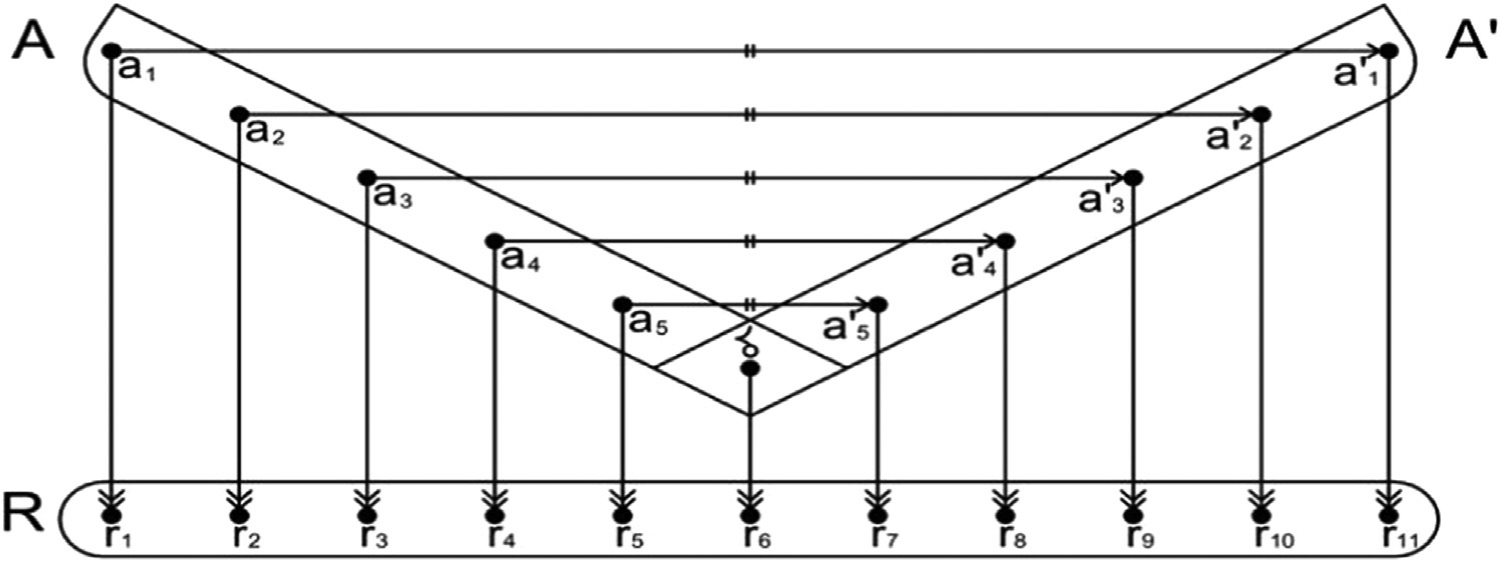
Linear mapping between an autoreflective pair of points and linear *re-mapping* with the optiscale. A represents the domain, while $A^{\prime }$ represents the co-domain of A. δ denotes a symmetrical line, and R represents the optiscale. The symbol  indicates a bijective mapping between the autoreflective pair around a symmetrical line, and  indicates a linear re-mapping with the optiscale R.

**Fig. 2:**
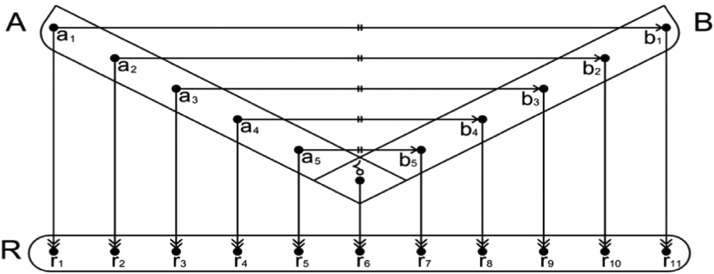
Linear mapping between an isoreflective pair of points and linear *re-mapping* with the optiscale. A represents the domain, while B represents the co-domain of A. δ denotes a central point, and R represents the optiscale. The symbol  indicates a bijective mapping between the isoreflective pair around a central point, and  indicates a linear re-mapping with the optiscale R.

Optinalysis is a function that is comprised of an assigned optiscale (R) that bijectively re-maps (a symbol  indicates a re-mapping) an autoreflective or isoreflective pair of mathematical structures. Figs. 1 and 2 illustrates how autoreflective and isoreflective pairs of points are mapped and also re-mapped with an optiscale.

Optinalysis is expressed in *optinalytic construction*. An optinalytic construction is the mathematical representation of optinalysis between isoreflective or autoreflective pairs as bijective linear mappings. Optinalysis is defined in two broad types: automorphic (shape) and isomorphic (comparative) optinalysis.

Automorphic (shape) optinalysis:

Automorphic or shape optinalysis refers to the analysis of autoreflective pairs of a mathematical structure by optinalysis. It is a method of symmetry/asymmetry and identity estimation.

Let P be an autoreflective pair of mathematical structure A and its mirror image $A^{\prime }$ about a center δ. Let  indicate a linear re-mapping. Let R be the assigned optiscale. Then, automorphic optinalysis as a function is defined as:

f:P→R

The autoreflective pair P of the mathematical structure A and its mirror image $A^{\prime }$ have been defined as . Now optiscale R is introduced into the function to establish a linear re-mapping with the P. We now have new representations (called optinalytic constructions, all of which provide the same definition but in different representations) as:

Such that $\delta \in A,A^{\prime }$;$\;A,\;A^{\prime },\delta \in R$;$R\subseteq \{r\in R^{+*}|r=n*k,\;n\in N,\;k>0\}$ or alternatively $R\subseteq \{r\in R^{-*}|r=-n*k,\;n\in N,\;k>0\}$; n∈N; and $A\;\&\;A^{\prime }$ is an autoreflective about a central point δ.

Isomorphic (comparative) optinalysis:

Isomorphic or comparative optinalysis refers to the analysis of isoreflective pairs of mathematical structures by optinalysis. It is a method of similarity/dissimilarity and identity estimation. Comparative optinalysis is defined by its optinalytic construction as follows:

Let P be an isoreflective pair of mathematical structures A and B about a center δ. Let  indicate a linear re-mapping. Let R be the assigned optiscale. Then, isomorphic optinalysis as a function is defined as:

f:P→R

The isoreflective pair P of the two mathematical structures A and B has been defined as . Now optiscale R is introduced into the function to establish a linear re-mapping with the P. We now have new representations (called optinalytic constructions):

Such that δ∉A,B;A,B,δ∈R;$R\subseteq \{r\in R^{+*}|r=n*k,\;n\in N,\;k>0\}$ or alternatively $R\subseteq \{r\in R^{-*}|r=-n*k,\;n\in N,\;k>0\}$; n∈N; and A&B are isoreflective pairs on a chosen pairing about a central point δ.

Definition 11 Scalement

A *scalement* refers to the product of any member of an autoreflective or isoreflective pair of mathematical structures and its assigned optiscale.

Let the optinalytic construction of an isoreflective pair of two mathematical structures A and B with an assigned optiscale (R) be:

Such that δ∉A,B;A,B,δ∈R;$R\subseteq \{r\in R^{+*}|r=n*k,\;n\in N,\;k>0\}$ or alternatively $R\subseteq \{r\in R^{-*}|r=-n*k,\;n\in N,\;k>0\}$; n∈N; and A&B are isoreflective pairs on a chosen pairing about a central point δ.

Then, the sum of scalements S of the isoreflective pair between mathematical structures A and B is defined as:

$$S\left(A,B\right)=\left(r_{1}.a_{1}\right)+\ldots +\left(r_{n+1}.\delta \right)+\ldots +\left(r_{2n+1}.b_{1}\right)=\sum_{i=1}^{n}\sum_{j=k=n+2}^{2n+1}\left(r_{i}a_{i}+r_{n+1}\delta +r_{j}b_{i}\right)$$

Definition 12 Kabirian coefficient of automorphic optinalysis

The level of symmetry or identity of an autoreflective pair of a mathematical structure under optinalysis is defined by the optinalytic coefficient, known as the Kabirian coefficient (Kc). The Kabirian coefficient for automorphic optinalysis is expressed as the quotient of the product of the median optiscale and the summation of all elements (of the autoreflective pair) divided by the summation of all scalements (of the autoreflective pair).

Let the optinalytic constructions of autoreflective pair of a mathematical structure A and $A^{\prime }$ with an assigned optiscale (R) be:

Such that $\delta \in A,A^{\prime }$;$\;A,\;A^{\prime },\delta \in R;R\subseteq \left\{r\in R^{+*}|r=n*k,\;n\in N,\;k>0\right\}$ or alternatively $R\subseteq \{r\in R^{-*}|r=-n*k,\;n\in N,\;k>0\}$; n∈N; and $A\;\&\;A^{\prime }$ are autoreflective pairs about a central point δ.

Then, the Kabirian coefficient of symmetry or identity between the autoreflective pair is expressed by Eqs. (1.1) and (1.2)

(1.1) $$KC_{Sym./Id.}\left(A,A^{\prime }\right)=\frac{r_{\frac{n+1}{2}}\left(a_{1}+a_{2}+a_{3}+\ldots +a_{\frac{n-1}{2}}+\;{\hat{a}}_{\frac{n+1}{2}}+a{{}^{\prime }}_{\frac{n-1}{2}}+\ldots +a{{}^{\prime }}_{3}+a{{}^{\prime }}_{2}+a{{}^{\prime }}_{1}\right)}{\begin{array}{c} \left(r_{1}.a_{1}\right)+\left(r_{2}.a_{2}\right)+\left(r_{3}.a_{3}\right)+\ldots +\left(r_{\frac{n-1}{2}}.a_{\frac{n-1}{2}}\right)+\left(r_{\frac{n+1}{2}}.{\hat{a}}_{\frac{n+1}{2}}\right)+\\ \left(r_{\frac{n+3}{2}}.a{{}^{\prime }}_{\frac{n-1}{2}}\right)+\ldots +\left(r_{n-2}.a{{}^{\prime }}_{3}\right)+\left(r_{n-1}.a{{}^{\prime }}_{2}\right)+\left(r_{n}.a{{}^{\prime }}_{1}\right) \end{array}}$$

(1.2) $$KC_{Sym.\;/Id.}\left(A,A^{\prime }\right)=\frac{r_{\frac{n+1}{2}}\left[\sum_{i=1}^{\frac{n-1}{2}}\left(a_{i}+{\hat{a}}_{\frac{n+1}{2}}+a{{}^{\prime }}_{i}\right)\right]}{\sum_{i=1}^{\frac{n-1}{2}}\sum_{j=n}^{\frac{n+3}{2}}\left(r_{i}a_{i}+r_{\frac{n+1}{2}}{\hat{a}}_{\frac{n+1}{2}}+r_{j}a{{}^{\prime }}_{i}\right)}$$

$$\left\{ \begin{array}{c} if\;g\left(A\right)=g\left(A^{\prime }\right);\;then\;KC_{Sim.\;/Id.}\left(A,A^{\prime }\right)=1\\ if\;g\left(A\right)=-g\left(A^{\prime }\right),\;or\;-\;g\left(A\right)=g\left(A^{\prime }\right);\;then\;KC_{Sim.\;/Id.}\left(A,A^{\prime }\right)=0\\ if\;g\left(A\right)<g\left(A^{\prime }\right);\;then\;0\leq \;KC_{Sim.\;/Id.}\left(A,A^{\prime }\right)\leq 1\\ if\;g\left(A\right)>g\left(A^{\prime }\right);\;then\;1\leq KC_{Sim.\;/Id.}\left(A,A^{\prime }\right)\leq n+1\\ if\;g\left(A\right)>g\left(A^{\prime }\right);\;then\;KC_{Sim.\;/Id.}\left(A,A^{\prime }\right)\geq n+1,<0 \end{array}\right.$$

Where g(A) and $g(A^{\prime })$ are the optical moments of A and $A^{\prime }$ respectively about a central point of a symmetric distance D. It is expressed by Eqs. (2.1) and (2.2).

(2.1) $$\begin{array}{c} g\left(A\right)=\left(d_{k}.a_{1}\right)+\left(d_{k-1}.a_{2}\right)+\left(d_{k-2}.a_{3}\right)+\cdots +\left(d_{1}.a_{\frac{n-1}{2}}\right)=\sum_{i=1}^{k}\sum_{j=1}^{\frac{n-1}{2}}\left(d_{i}a_{j}\right) \end{array}$$

(2.2) $$\begin{array}{c} g\left(A^{\text{'}}\right)=\left(d_{k}.{a^{\text{'}}}_{1}\right)+\left(d_{k-1}.{a^{\text{'}}}_{2}\right)+\left(d_{k-2}.{a^{\text{'}}}_{3}\right)+\cdots +\left(d_{1}.{a^{\text{'}}}_{\frac{n-1}{2}}\right)=\sum_{i=1}^{k}\sum_{j=1}^{\frac{n-1}{2}}\left(d_{i}{a^{\text{'}}}_{j}\right) \end{array}$$

Such that $A,A^{\prime },D\in R$;D{x|x≤n,x≥−n,n>0}; n,k∈N;$k=\frac{n-1}{2}$ and $A\;\&\;A^{\prime }$ are isoreflective pair about a central point δ.

Definition 13 Kabirian coefficient of isomorphic optinalysis

The level of similarity or identity of an isoreflective pair of mathematical structures under optinalysis is defined by the optinalytic coefficient, known as the Kabirian coefficient (Kc). The Kabirian coefficient for isomorphic optinalysis is expressed as the quotient of the product of the median optiscale and the summation of all elements (of the isoreflective pair) divided by the summation of all scalements (of the isoreflective pair).

Let the optinalytic constructions of isoreflective pair of two mathematical structures A and B with an assigned optiscale (R) be:

Such that δ∉A,B;A,B,δ∈R;$R\subseteq \{r\in R^{+*}|r=n*k,\;n\in N,\;k>0\}$ or alternatively $R\subseteq \{r\in R^{-*}|r=-n*k,\;n\in N,\;k>0\}$; n∈N; and A&B are isoreflective pairs on a chosen pairing about a central point δ.

Then, the Kabirian coefficient of similarity or identity between the isoreflective pair is expressed by Eqs. (3.1) and (3.2).

(3.1) $$KC_{Sim.\;/Id.}\left(A,B\right)=\frac{r_{n+1}\left(a_{1}+a_{2}+a_{3}+\ldots +a_{n}+\;\delta +b_{n}+\ldots +b_{3}+b_{2}+b_{1}\right)}{\begin{array}{c} \left(r_{1}.a_{1}\right)+\left(r_{2}.a_{2}\right)+\left(r_{3}.a_{3}\right)+\ldots +\left(r_{n}.a_{n}\right)+\left(r_{n+1}.\delta \right)+\\ \left(r_{n+2}.b_{n}\right)+\ldots +\left(r_{2n-1}.b_{3}\right)+\left(r_{2n}.b_{2}\right)+\left(r_{2n+1}.b_{1}\right) \end{array}}$$

(3.2) $$KC_{Sim.\;/Id.}\left(A,B\right)=\frac{r_{n+1}\left[\sum_{i=1}^{n}\left(a_{i}+\delta +b_{i}\right)\right]}{\sum_{i=1}^{n}\sum_{j=n+2}^{2n+1}\left(r_{i}a_{i}+r_{n+1}\delta +r_{j}b_{i}\right)}$$

$$\left\{ \begin{array}{c} if\;g\left(A\right)=g\left(B\right);\;then\;KC_{Sim.\;/Id.}\left(A,B\right)=1\\ if\;g\left(A\right)=-g\left(B\right),\;or\;-\;g\left(A\right)=g\left(B\right);\;then\;KC_{Sim.\;/Id.}\left(A,B\right)=0\\ if\;g\left(A\right)<g\left(B\right);\;then\;0\leq \;KC_{Sim.\;/Id.}\left(A,B\right)\leq 1\\ if\;g\left(A\right)>g\left(B\right);\;then\;1\leq KC_{Sim.\;/Id.}\left(A,B\right)\leq n+1\\ if\;g\left(A\right)>g\left(B\right);\;then\;KC_{Sim.\;/Id.}\left(A,B\right)\geq n+1,<0 \end{array}\right.$$

Where g(A) and g(B) are the optical moments of A and B respectively about the central point of a symmetric distance D. It is expressed by Eqs. (4.1) and (4.2).

(4.1) $$g\left(A\right)=\left(d_{n}.a_{1}\right)+\left(d_{n-1}.a_{2}\right)+\left(d_{n-2}.a_{3}\right)+\ldots +\left(d_{1}.a_{n}\right)=\sum_{i=1}^{n}\left(d_{i}a_{i}\right)$$

(4.2) $$g\left(B\right)=\left(d_{n}.b_{1}\right)+\left(d_{n-1}.b_{2}\right)+\left(d_{n-2}.b_{3}\right)+\ldots +\left(d_{1}.b_{n}\right)=\sum_{n=1}^{n}\left(d_{i}b_{i}\right)$$

Such that A,B,D∈R;D{x|x≤n,x≥−n,n>0};n∈N; and A&B are isoreflective pair in a chosen pairing about a central point δ.

## Additional information

 

## Introduction

**General overview:** In the domains of mathematics and biology, the assessment of symmetry/asymmetry, similarity/dissimilarity, and identity/unidentity within structures and sequences holds immense significance. These estimations underpin diverse applications, ranging from deciphering the hidden patterns in mathematical structures to unraveling the evolutionary relationships among biological sequences. However, the existing methods for these estimations often lack a unified theoretical foundation, leading to inconsistent interpretations and limited comparability across different domains [1], [2], [3], [4], [5], [6], [7], [8], [9], [10], [11], [12], [13], [14], [15], [16], [17], [18], [19], [20], [21], [22], [23], [24], [25], [26], [27], [28], [29], [30], [31], [32], [33], [34], [35], [36], [37]. Addressing these issues necessitates the development of a novel framework that not only unites the diverse estimates under a common theoretical paradigm but also enhances their accuracy, reliability, and interpretability.

**Background and Research Problems:** The exploration of symmetry/asymmetry, similarity/dissimilarity, and identity/unidentity estimations has been instrumental in revealing essential characteristics of various mathematical structures and biological sequences. Symmetry/asymmetry serves as a fundamental principle in both domains, manifesting in the repetitive patterns of geometric shapes and the conserved motifs of genetic sequences [1], [2], [3], [4], [5], [6], [7], [8], [9], [10], [11], [12], [13]. Similarly, concepts of similarity and identity offer insights into the structural and functional relationships among objects and sequences, guiding investigations in fields as diverse as image analysis and phylogenetics [8], [14], [15], [16], [17], [18], [19], [20], [21], [22], [23], [24], [25], [26], [27], [28], [29], [30], [31], [32], [33], [34], [35], [36], [37].

However, the lack of a cohesive theoretical framework for these estimations has led to numerous challenges. Existing methods often vary in their theoretical underpinnings and computational approaches, rendering comparisons and interpretations convoluted [1], [2], [3], [4], [5], [6], [7], [8], [9], [10], [11], [12], [13], [14], [15], [16], [17], [18], [19], [20], [21], [22], [23], [24], [25], [26], [27], [28], [29], [30], [31], [32], [33], [34], [35], [36], [37]. The imperative to unify these estimates under a shared theoretical foundation is underscored by the need to ensure accurate, reliable, and comparable estimations that possess desirable statistical properties. This includes properties like robustness (invariance to scaling and location shifts), unbiasedness, efficiency, and resistance to outliers.

Furthermore, while these estimations have foundations in mathematical concepts such as isomorphism and automorphism[38], [39], [40], many of their associated estimators and functions are not always readily validated or proven using these concepts [1], [2], [3], [4], [5], [6], [7], [8], [9], [10], [11], [12], [13], [14], [15], [16], [17], [18], [19], [20], [21], [22], [23], [24], [25], [26], [27], [28], [29], [30], [31], [32], [33], [34], [35], [36], [37]. This disconnect raises questions about the conceptual integrity and validity of the estimation techniques employed.

**Primary Objective:** The primary objective of this paper is to introduce a novel method termed “Kabiran-based optinalysis.” This method is designed to establish a theoretically grounded framework for symmetry/asymmetry, similarity/dissimilarity, and identity/unidentity estimations by drawing inspiration from the theories of isomorphism and automorphism. Unlike previous approaches, which lack a comprehensive theoretical grounding, the Kabiran-based optinalysis method aims to provide estimators and functions that are firmly rooted in these foundational concepts. By doing so, this method seeks to address the inconsistencies and limitations associated with existing techniques.

**Secondary Objective:** In addition to the primary objective, this paper also pursues a secondary goal: the development of an algorithm and estimating techniques known as “geometrical pairwise analysis.” This approach, built upon the foundations of Kabiran-based optinalysis, is specifically tailored for the pairwise analysis of biological sequences. This secondary objective is driven by the inadequacies of conventional percentage similarity metrics in capturing position-specific and character-specific variations between biological sequences. The geometrical pairwise analysis seeks to offer a more robust and sensitive alternative, enhancing our ability to discern meaningful patterns and relationships in biological sequences.

**Significance of the Study:** The proposed framework of Kabirian-based optinalysis carries profound significance for the advancement of knowledge in both mathematical analysis and biological research. By unifying symmetry/asymmetry, similarity/dissimilarity, and identity/unidentity estimations under a common theoretical umbrella, the study introduces a new perspective that transcends disciplinary boundaries. This holistic approach has the potential to revolutionize the way researchers analyze and interpret mathematical structures and biological sequences.

The implications of this study extend beyond the immediate domains of mathematics and biology. The development of a theoretically and conceptually grounded framework for estimations has the potential to contribute to a wide array of research communities and fields of study. The establishment of robust, accurate, and interpretable estimations can catalyze progress in fields as diverse as computer science, data analysis, pattern recognition, and evolutionary biology.

**Limitations of the Study:** It's important to acknowledge the limitations of this study. The application of the developed Kabiran-based optinalysis is currently limited to the geometric pairwise analysis of biological sequences. While this serves as a critical step towards enhancing the accuracy and sensitivity of pairwise analysis, it does not exhaustively address all aspects of symmetry/asymmetry, identity/unidentity, and other dimensions of similarity/dissimilarity estimations. Future research endeavors could explore the extension of the framework to encompass a broader range of estimations and applications.

**Conclusion:** The pursuit of a unified theoretical foundation for symmetry/asymmetry, similarity/dissimilarity, and identity/unidentity estimations in mathematical structures and biological sequences is a paramount endeavor. The Kabiran-based optinalysis framework and geometrical pairwise analysis techniques strive to overcome the challenges posed by the lack of theoretical cohesion in current estimation methods. By providing validated and proven functions rooted in isomorphism and automorphism theories, this study opens new avenues for accurate, reliable, and interpretable estimations across diverse domains. The potential to advance knowledge and contribute to a spectrum of research communities underscores the importance of this study in addressing pressing research problems and fostering interdisciplinary progress.

## Literature Review

**Introduction:** The estimation of symmetry/asymmetry, similarity/dissimilarity, and identity/unidentity in mathematical structures and biological sequences is a fundamental pursuit with implications across diverse domains. Existing approaches, however, lack a unified theoretical foundation, leading to inconsistencies and challenges in interpretation. This literature review aims to comprehensively explore the concepts, theories, and prior research relevant to this topic, ultimately paving the way for the introduction of the Kabirian-based optinalysis framework and its associated methodologies.

**Theoretical Foundations: Isomorphism and Automorphism:**

In this section, we established the concept of automorphism, and isomorphism as mathematical functions are reviewed to highlight how they frame and underpin the foundation of symmetry, similarity, and identity. These references provide some useful explanations [38], [39], [40].

### Isomorphism

In the realm of mathematical structures, isomorphism is a fundamental concept that yields profound insights into structural relationships. Essentially, it defines a notion of similarity or identity between two mathematical objects, often denoted as follows:

• Mathematical Representation of Isomorphism:
  • An isomorphism is symbolized by a mapping function, f, denoted as f:A→B, where A and B are mathematical structures.
  • Isomorphism is denoted as A≅B, indicating that structures A and B are isomorphic, signifying their structural equivalence.

*Bijective Correspondence:*

• For a mapping to be considered an isomorphism, it must establish a bijective correspondence. This means that the mapping, represented by φ, is both injective (one-to-one) and surjective (onto). In simpler terms, each element in A uniquely corresponds to an element in B, and vice versa.

*Preservation of Structure* ***:***

• Isomorphism rigorously preserves the underlying structure and properties of structures A and B. This preservation encompasses the relationships between elements and any operations defined within these structures.
• Mathematically, for any operation, denoted as f, defined in structure A, the following condition holds: $f\left(a,\;b\right)\;=\;f^{\prime }\left(\varphi (a),\;\varphi (b)\right)$, where f is an operation in A, and $f^{\prime }$ is the corresponding operation in B.
• Isomorphism enables mathematicians to recognize and establish that, from a structural perspective, A and B are indistinguishable.

### Automorphism

Automorphism, an extension of isomorphism, delves into the symmetrical properties within a single mathematical structure. It is, in essence, a way of mapping a structure to itself while preserving all of its mathematical structure, including vertices, edges, non-edges, and connections.

• Mathematical Representation of Automorphism:
• Automorphism is symbolized as a mapping function, f, denoted as $f:A\to Aut(A^{\prime })$, where A represents the mathematical structure, and $Aut(A^{\prime })$ represents the set of all automorphisms of A.

*Bijective Self-Correspondence:*

• Similar to isomorphism, an automorphism, represented by ψ, is a bijection within A. It ensures a one-to-one and onto mapping of elements within the structure.

*Intrinsic Characteristics Preservation:*

• Automorphism conserves the intrinsic characteristics and properties of structure A. This implies that any operation or relationship defined within A remains intact under the automorphism ψ.
• Mathematically, for any operation, denoted as g, in structure A, the following condition holds: $g\left(a,\;b\right)\;=\;g^{\prime }\left(\psi (a),\;\psi (b)\right)$, where g is an operation in A, and $g^{\prime }$ is the corresponding operation under ψ.

Automorphisms unveil the internal symmetries of a mathematical structure, revealing how the structure can transform while retaining its essential attributes. They provide insights into self-similarity and self-consistency within the structure itself.

## Definition of Injections, Surjections, and Bijections

In addition to isomorphism and automorphism, understanding the concepts of injections, surjections, and bijections of functions is crucial:

• An injection, often called a one-to-one function, is a function that maps distinct elements to distinct elements. This means that if x≠y, then f(x)≠f(y). Equivalently, iff(x)=f(y), then x=y.
• A surjection, also known as onto function, includes all elements of B in its image. In other words, for every y∈B, there exists an x∈A such that f(x)=y.
• A bijection, often referred to as a one-to-one and onto correspondence, is a function that is simultaneously injective and surjective. Another way to describe a bijection, f:A→B, is that there exists an inverse function, g:B→A, such that the composition g o f:A→A is the identity function on A, and fog:B→B is the identity function on B. The inverse function is commonly denoted as $f^{-1}$.

Furthermore, bijections have a crucial property, which allows equations involving f to be converted into equations involving $f^{-1}$. This property states that f(x)=y if and only if $x=f^{-1}\left(y\right)$.

## Challenges and Gaps

While isomorphism and automorphism provide a solid basis for understanding structural relationships, their direct application to estimation techniques is not always straightforward. Many estimators and functions utilized for symmetry/asymmetry, similarity/dissimilarity, and identity/unidentity analyses draw inspiration from these concepts, yet validation and proof through isomorphism and automorphism theories are often lacking. This presents a significant gap in the conceptual and theoretical underpinnings of these estimation methods.

**Symmetry/Asymmetry Estimations:**

Symmetry/asymmetry estimation in mathematics identifies reflection, rotation, and translation patterns, revealing structural similarities and transformations within mathematical objects. Table 1 presents a summary of various estimators used for symmetry and asymmetry measurements, highlighting their diversity in mathematical foundations, theoretical frameworks, applications, and limitations. These estimators, despite their importance, currently lack a unified theoretical framework among symmetry and asymmetry estimators can be attributed to several inherent challenges and factors [1], [2], [3], [4], [5], [6], [7], [8], [9], [10], [11], [12], [13]:

1. Diversity of Analytical Goals: Symmetry and asymmetry estimators serve a wide range of analytical goals. Some are designed to measure the degree of symmetry in geometric shapes, while others focus on characterizing asymmetry in statistical distributions or biological structures. These varying goals necessitate different mathematical approaches.
2. Varied Mathematical Foundations: These estimators are based on diverse mathematical foundations. For instance, moments and Fourier analysis rely on advanced mathematical concepts, while simple measures like skewness and kurtosis are rooted in statistics. Attempting to unify such distinct mathematical underpinnings can be complex.
3. Context-Dependent Use: The choice of estimator depends on the specific context and data type. For example, structural alignment methods are specifically tailored to analyze biological structures, while Fourier analysis is more suited for periodic data. Unifying these under a single theoretical framework may lead to a loss of context specificity.
4. Specialization in Fields: Many symmetry and asymmetry estimators have evolved within specific fields such as mathematics, physics, biology, and statistics. They have been developed to address unique challenges within these domains and often conform to domain-specific theories and practices.
5. Complexity of Estimators: Some estimators, like gyration radius or structural alignment, deal with complex and specialized mathematical concepts that are not easily reconciled with simpler measures. Attempting to unify them may lead to impractical complexity.
6. Historical Development: Estimators have been developed independently over time to address specific problems. Their historical development paths have contributed to their uniqueness, making unification challenging.
7. Data Heterogeneity: Symmetry and asymmetry estimators are applied to a wide variety of data types, including numeric data, biological sequences, structural information, and more. Developing a unified framework that accommodates this data heterogeneity is complex.
8. Practicality and Applicability: While unification may offer conceptual clarity, it often comes at the cost of practicality and adaptability to real-world applications. Different estimators are better suited to specific tasks and data types, making them valuable in their respective domains.

**Table 1:** Various estimators used for symmetry and asymmetry measurements.

Estimator	Mathematical Foundation	Theoretical Framework	Field of Application	Limitations/Weaknesses	Reference
Skewness	Statistics	Probability Theory	Finance, Economics	Sensitive to data distribution, may not capture all asymmetries.	[1]
Kurtosis	Statistics	Probability Theory	Finance, Engineering	Highly affected by outliers, measures tail behavior.	[2]
Moments	Statistics	Probability Theory	Engineering, Physics	Higher moments are less interpretable, and data-dependent.	[3]
Fourier Analysis	Mathematics, Physics	Harmonic Analysis	Signal Processing, Physics	Assumes periodicity, not suitable for all data types.	[4]
Fractal Dimension	Mathematics, Chaos Theory	Fractal Geometry	Image Analysis, Geophysics	Interpretation can be challenging, and data-specific.	[5]
Gyration Radius	Physics, Molecular Modeling	Geometric Analysis	Chemistry, Biology	Sensitive to structure changes, may require complex modeling.	[6]
Symmetry Groups	Group Theory	Algebraic Structures	Crystallography, Mathematics	Limited to specific mathematical structures.	[7]
Sequence Alignment	Bioinformatics	Graph Theory	Genetics, Proteomics	Computationally intensive, may not handle large datasets.	[8]
Symmetry Index	Image Processing	Computer Vision	Biology, Robotics	Relies on image quality and preprocessing.	[9]
Radial Distribution	Chemistry, Physics	Statistical Mechanics	Materials Science, Chemistry	Requires specialized equipment and analysis.	[10]
Gini Coefficient	Economics, Statistics	Inequality Theory	Economics, Sociology	Sensitivity to distributional changes is not suitable for all data.	[11]
Mahalanobis Distance	Statistics, Mathematics	Multivariate Analysis	Statistics, Outlier Detection	Sensitive to data dimensionality.	[12]
Entropy	Information Theory	Information Theory	Computer Science, Physics	Highly dependent on data representation.	[13]

Please note that this table offers a simplified overview. Each estimator may have various subtypes and methods, and the field of application can be much broader and nuanced. Additionally, the provided references can be used to delve into further details and sources for each estimator.

In summary, the lack of unification among symmetry and asymmetry estimators is primarily due to the diverse goals, mathematical foundations, contexts, and historical developments associated with these estimators. While unification could provide a theoretical framework, it may not always be practical or suitable given the complexity and specificity of these estimators in addressing different analytical challenges. Researchers continue to explore ways to bridge the gaps and establish connections between these estimators while respecting their unique strengths and purposes in various fields of study.

**Similarity and Identity Estimations:**

In both mathematical structures and biological sequences, assessing similarity and identity is essential for pattern recognition, evolutionary analysis, and information retrieval. Table 2 summarizes various estimators used for similarity, dissimilarity, identity, and unidentity measurements across different fields. These estimators lack a unified theoretical framework due to their diverse mathematical foundations and applications. The lack of a unified theoretical framework arises from the intrinsic diversity of these estimators. They have evolved independently to address specific analytical challenges in various domains [8], [14], [15], [16], [17], [18], [19], [20], [21], [22], [23], [24], [25], [26]. Here's why they lack unification:

1. Diverse Data Types: These estimators are designed to handle vastly different data types, including numeric vectors, sets, strings, and probability distributions. Developing a single framework to encompass all these data types is challenging.
2. Distinct Analytical Goals: Estimators serve different analytical goals, such as measuring similarity between vectors, sets, or sequences, making it difficult to find a one-size-fits-all theoretical framework.
3. Mathematical Complexity: The underlying mathematics of these estimators can be highly complex and specialized. Attempting to create a unified framework may lead to impractical complexity or loss of specificity.
4. Field-Specific Requirements: Some estimators are deeply ingrained in specific fields like bioinformatics or structural biology, where they must adhere to domain-specific theories and practices.
5. Limitations and Weaknesses: Each estimator has its limitations, such as sensitivity to outliers, dependence on data representation, and computational intensity, making them suitable for some contexts but not others.

**Table 2:** Various estimators used for similarity, dissimilarity, identity, and unidentity measurements.

Estimator	Mathematical Foundation	Theoretical Framework	Field of Application	Limitations/Weaknesses	Reference
Euclidean Distance	Mathematics, Geometry	Metric Space Theory	Mathematics, Computer Vision	Sensitive to scale and outliers.	[14]
Cosine Similarity	Linear Algebra, Statistics	Vector Space Theory	Natural Language Processing	Ignores magnitude, context-dependent.	[15]
Jaccard Similarity	Set Theory	Set Theory	Text Analysis, Data Mining	Not suitable for continuous data.	[16]
Hamming Distance	Information Theory	Binary Space	Genetics, Error Detection	Limited to binary data.	[17]
Manhattan Distance	Mathematics, Geometry	Metric Space Theory	Urban Planning, Image Analysis	Sensitive to coordinate system.	[18]
Minkowski Distance	Mathematics, Geometry	Metric Space Theory	Cluster Analysis, Pattern Recognition	Depends on the choice of 'p'.	[19]
Pearson Correlation	Statistics, Linear Algebra	Correlation Theory	Statistics, Data Analysis	Sensitive to outliers.	[20]
Mahalanobis Distance	Statistics, Linear Algebra	Multivariate Analysis	Multivariate Statistics, Clustering	Assumes multivariate normality.	[21]
Levenshtein Distance	Computer Science, Algorithms	Edit Distance Theory	Spell Checking, DNA Comparison	Computationally intensive.	[22]
Sequence Alignment	Bioinformatics	Dynamic Programming	Genetics, Proteomics	Computationally intensive.	[8]
Structural Alignment	Biology, Chemistry	Structural Biology	Protein Structure Prediction	Highly dependent on data quality.	[23]
Tanimoto Coefficient	Statistics, Chemistry	Set Theory	Chemistry, Chemoinformatics	Sensitive to data representation.	[24]
Jensen-Shannon Divergence	Information Theory, Statistics	Probability Distributions	Information Retrieval, NLP	Data-specific requires probability distributions.	[25]
Gower's Distance	Statistics, Ecology	Multivariate Analysis	Ecological Studies, Clustering	Sensitive to data types.	[26]

Please note that this table provides a simplified overview. Each estimator may have various subtypes and methods, and the field of application can be much broader and nuanced.

In essence, the diversity in data types, analytical goals, and mathematical foundations has led to the development of distinct estimators tailored to specific challenges. While unification may offer conceptual clarity, it often comes at the cost of practicality and adaptability to real-world applications. Researchers continue to explore ways to bridge the gaps between these estimators while respecting their unique strengths and purposes.

## Pairwise Analysis of Biological Sequences

Pairwise analysis of biological sequences involves comparing two genetic or protein sequences to identify similarities and differences. It's fundamental for understanding evolutionary relationships, gene function, and disease mechanisms. Its scope encompasses DNA, RNA, and protein sequences, enabling researchers to uncover vital information about genetic evolution, structural motifs, and functional domains. This analysis is essential in fields like genomics, drug discovery, and evolutionary biology, aiding in the development of treatments and our understanding of life processes. Table 3 includes a range of sequence alignment algorithms used to measure similarity or identity between biological sequences. Some of these algorithms compute a percentage of aligned residues as a metric. However, they all share a common limitation: they primarily focus on the number of identical residues without considering the position or character involved in variations [28], [29], [30], [31], [32], [33], [34], [35], [36], [37].

**Table 3:** Sequence Alignment Algorithms Used to Measure Similarity and Identity between Biological Sequences.

Metric/Estimator	Mathematical Foundation	Theoretical Framework	Field of Application	% Aligned Residues	Limitations/Weaknesses	Reference
Hamming Distance	Discrete mathematics	Set theory	Genetics, genomics	No	Only for sequences of equal length; sensitive to gaps	[27]
Levenshtein Distance	Dynamic programming	String algorithms	Sequence alignment, spell checking	No	Computationally intensive; not suitable for continuous data	[22]
Needleman-Wunsch	Dynamic programming	Pairwise alignment	Bioinformatics, molecular biology	Yes	Computationally intensive; not suitable for large datasets	[28]
Smith-Waterman	Dynamic programming	Pairwise alignment	Sequence comparison	Yes	Computationally intensive; not suitable for large datasets	[29]
BLAST	Heuristic algorithms	Sequence similarity	Sequence database search	Yes	Approximate search; sensitivity to parameter settings	[30]
FASTA	Heuristic algorithms	Sequence similarity	Sequence database search	Yes	Approximate search; sensitivity to parameter settings	[31]
Stretcher	Dynamic programming	Pairwise alignment	Sequence alignment	Yes	Allows affine gap penalties; customizable scoring schemes	[32]
Matcher	Heuristic algorithms	Sequence similarity	Sequence comparison	Yes	Handles very long sequences; scalable	[33]
MAFFT	Progressive alignment	Multiple alignment	Multiple sequence alignment	Yes	Scalable; multiple alignment capabilities	[34]
MUSCLE	Progressive alignment	Multiple alignment	Multiple sequence alignment	Yes	High accuracy; scalable	[35]
Clustal Omega	Progressive alignment	Multiple alignment	Multiple sequence alignment	Yes	Speed and accuracy improvements; handles large datasets	[36]
T-Coffee	Progressive alignment	Multiple alignment	Multiple sequence alignment	Yes	Handles diverse data types; Versatile	[37]

### Weaknesses of Percentage-Aligned Residues Metrics

The weakness of algorithms that rely solely on the percentage of aligned residues can be illustrated with the following examples using aligned sequences:

Example Aligned Sequences:

seq_0 = TACTGGTCAG
seq_1 = TACTG***A***TCAG
seq_2 = TA***G***TGGTCAG
seq_3 = TACTG **-** CAG

Percentage Similarity/Identity Calculation:

Pairwise percentage similarity/identity between seq_0 and all others:

• Percentage similarity/identity between seq_0 and seq_1 = 9/10 * 100 = 90%
• Percentage similarity/identity between seq_0 and seq_2 = 9/10 * 100 = 90%
• Percentage similarity/identity between seq_0 and seq_3 = 9/10 * 100 = 90%

In the above examples, the percentage similarity or identity calculations only consider the number of identical residues (matches) and ignore position-specific and character-specific variations, such as substitutions (mismatches) and indels (insertions/deletions).

### Limitations and the Need for Robust Algorithms

The weaknesses of percentage-aligned residues metrics are evident:

• They do not differentiate between matches, mismatches, indels, and other variations.
• They treat all matches, mismatches, or indels equally, even if they occur at critical positions.
• They do not account for the biological significance of specific residues, such as transition, transversion, baric, and isobaric variations in aligned biological sequences. This is more pronounced with percentage similarity metrics.
• They do not account for the holistic analysis and the comprehensive characterization of matches, mismatches, and indels.

As a result, these algorithms can lead to misleading similarity or identity estimates, especially when critical regions or residues are involved in variations. To address these limitations comprehensively and obtain more valid and reliable estimates and conclusions, there is a clear need for robust algorithms that consider position-specific and character-specific variations. Such algorithms should weigh matches, substitutions, and indels differently, giving a more nuanced view of sequence similarity and identity. Advanced algorithms such as those that use substitution matrices (e.g., BLOSUM), which assign different scores to different types of residue changes, and those that employ gap penalties to account for indels emphases on the quality of the alignment results but do not optimize the similarity metrics used. Although these robust algorithms provide a more biologically meaningful assessment of sequence similarity or identity, however, they have neglected other dimensions for improvement.

## Conceptual Integration and Validation

The integration of isomorphism and automorphism theories into the estimation of symmetry/asymmetry, similarity/dissimilarity, and identity/unidentity holds promise. However, many existing functions and estimators cannot be directly validated using these foundational concepts. This challenge has led to the pursuit of new methodologies that bridge the conceptual gap between theoretical principles and practical estimation techniques.

## Conclusion on the Review of Literature

The pursuit of a unified theoretical foundation for symmetry/asymmetry, similarity/dissimilarity, and identity/unidentity estimations is a complex and multi-disciplinary endeavor. The exploration of isomorphism, automorphism, and their integration into estimation techniques is crucial for advancing the coherence and validity of these analyses.

## Theorem in Kabirian-based optinalysis

The theorems in Kabirian-based optinalysis have the following highlights:

i. Kabirian-based automorphic optinalysis establishes a bijective relationship between elements of a mathematical structure and its mirror image when expressed as functions.
ii. Kabirian-based isomorphic optinalysis establishes a bijective relationship between corresponding elements of two mathematical structures when expressed as functions.
iii. Completeness invariance in optinalysis ensures estimates remain unchanged under transformations such as rotation, reflection, translation, and modulation when structures are completely symmetrical or similar.
iv. Incompleteness invariance in optinalysis ensures estimates remain unchanged under transformations such as product translation and central rotation when structures are incompletely symmetrical or similar.
v. Optinalytic normalization neutralizes the impact of incompleteness through central modulation, achieving near-completeness in incompletely symmetrical or similar structures.
vi. Kabirian-based optinalysis-to-probability translation models estimate unknown probabilities of symmetry, identity, or similarity using optinalysis coefficients and predefined optiscale, providing a relationship between actual and expected probabilities.

Theorem 1 *(functional bijectivity of Kabirian-based automorphic optinalysis)*

Theorem: Let $A=(x_{1},x_{2},\;x_{3},\ldots ,x_{n})$ and its mirror image$A^{\prime }=\left(x{{}^{\prime }}_{1},x{{}^{\prime }}_{2},\;x{{}^{\prime }}_{3},\ldots ,x{{}^{\prime }}_{n}\right)$ be a mathematical structure. If under Kabirian-based automorphic optinalysis ($K_{c}\left(A,A^{\prime }\right)$), and every element of A and $A^{\prime }$ is expressed as a function, then the resulting functions generate a bijective relationship between corresponding elements of A and $A^{\prime }$.

*Prove of* Theorem 1 *:*

Let the optinalytic construction of an autoreflective pair of symmetrical or identical mathematical structure A and its image $A^{\prime }$ be:

Such that $\delta \in A,A^{\prime }$;$\;A,A^{\prime },\delta \in R$;$R\subseteq \{r\in R^{+*}|r=n*k,\;n\in N,\;k>0\}$ or alternatively $R\subseteq \{r\in R^{-*}|r=-n*k,\;n\in N,\;k>0\}$; and A&B are autoreflective pairs about a central point δ.

By Kabirian-based optinalysis (i.e., in Eq. (5)), each element functions in Eqs. (5.1)–(6.7).

(5) $$K_{c}\left(A,A^{\prime }\right)=\frac{4\left(x_{1}+x_{2}+x_{3}+\;\delta +x{{}^{\prime }}_{3}+x{{}^{\prime }}_{2}+x{{}^{\prime }}_{1}\right)}{x_{1}+2x_{2}+3x_{3}+4\delta +5x{{}^{\prime }}_{3}+6x{{}^{\prime }}_{2}+7x{{}^{\prime }}_{1}}$$

(5.1) $$x_{1}=\frac{K_{c}\left(2x_{2}+3x_{3}+\;4\delta +5x{{}^{\prime }}_{3}+6x{{}^{\prime }}_{2}+7x{{}^{\prime }}_{1}\right)-4\left(x_{2}+x_{3}+\;\delta +x{{}^{\prime }}_{3}+x{{}^{\prime }}_{2}+x{{}^{\prime }}_{1}\right)}{4-K_{c}}$$

(5.2) $$x_{2}=\frac{K_{c}\left(x_{1}+3x_{3}+\;4\delta +5x{{}^{\prime }}_{3}+6x{{}^{\prime }}_{2}+7x{{}^{\prime }}_{1}\right)-4\left(x_{1}+x_{3}+\;\delta +x{{}^{\prime }}_{3}+x{{}^{\prime }}_{2}+x{{}^{\prime }}_{1}\right)}{4-2K_{c}}$$

(5.3) $$x_{3}=\frac{K_{c}\left(x_{1}+2x_{2}+\;4\delta +5x{{}^{\prime }}_{3}+6x{{}^{\prime }}_{2}+7x{{}^{\prime }}_{1}\right)-4\left(x_{1}+x_{2}+\;\delta +x{{}^{\prime }}_{3}+x{{}^{\prime }}_{2}+x{{}^{\prime }}_{1}\right)}{4-3K_{c}}$$

(5.4) $$\delta =\frac{K_{c}\left(x_{1}+2x_{2}+3x_{3}+5x{{}^{\prime }}_{3}+6x{{}^{\prime }}_{2}+7x{{}^{\prime }}_{1}\right)-4\left(x_{1}+x_{2}+x_{3}+x{{}^{\prime }}_{3}+x{{}^{\prime }}_{2}+x{{}^{\prime }}_{1}\right)}{4-4K_{c}}$$

(5.5) $$x{{}^{\prime }}_{3}=\frac{K_{c}\left(x_{1}+2x_{2}+3x_{3}+\;4\delta +6x{{}^{\prime }}_{2}+7x{{}^{\prime }}_{1}\right)-4\left(x_{1}+x_{2}+x_{3}+\;\delta +x{{}^{\prime }}_{2}+x{{}^{\prime }}_{1}\right)}{4-5K_{c}}$$

(5.6) $$x{{}^{\prime }}_{2}=\frac{K_{c}\left(x_{1}+2x_{2}+3x_{3}+\;4\delta +5x{{}^{\prime }}_{3}+7x{{}^{\prime }}_{1}\right)-4\left(x_{1}+x_{2}+x_{3}+\;\delta +x{{}^{\prime }}_{3}+x{{}^{\prime }}_{1}\right)}{4-6K_{c}}$$

(5.7) $$x{{}^{\prime }}_{1}=\frac{K_{c}\left(x_{1}+2x_{2}+3x_{3}+\;4\delta +5x{{}^{\prime }}_{3}+6x{{}^{\prime }}_{2}\right)-4\left(x_{1}+x_{2}+x_{3}+\;\delta +x{{}^{\prime }}_{3}+x{{}^{\prime }}_{2}\right)}{4-7K_{c}}$$

Recall the definition of bijective mapping (*one-to-one and onto*), such that if $x=x^{\prime }$, then $f\left(g(x)\right)=g\left(f(x^{\prime })\right)$. To verify that x and $x^{\prime }$ are bijective, three (3) cases of mathematical proof were evaluated (See Appendix **A** of the supplementary material). Based on the analysis of the proven cases, it is concluded that Kabirian-based automorphic optinalysis ($Opt(A,A^{\prime })$) is a construction and function that satisfied the bijective mapping which signifies automorphism of a defined mathematical structure. It was interesting to have verified that each pair of autoreflective points is bijective with a different optinalysis-derived function. That means the bijectivity of one pair-points is independent of the others.

Theorem 2 *(Functional bijectivity of Kabirian-based isomorphic optinalysis).*

Theorem: Let $A=(x_{1},x_{2},\;x_{3},\ldots ,x_{n})$ and $B=(y_{1},y_{2},\;y_{3},\ldots ,y_{n}$ be mathematical structures. If under Kabirian-based isomorphic optinalysis ($K_{c}\left(A,B\right)$), and every element of A and B is expressed as a function, then the resulting functions generate a bijective relationship between corresponding elements of A and B.

*Prove of* Theorem 2 *:*

Let the optinalytic construction of an isoreflective pair of similar or identical mathematical structures A and B be:

Such that δ∉A,B; A,B,δ∈R;$R\subseteq \{r\in R^{+*}|r=n*k,\;n\in N,\;k>0\}$ or alternatively $R\subseteq \{r\in R^{-*}|r=-n*k,\;n\in N,\;k>0\}$; and A&B are isoreflective pair on a chosen pairing about a central point δ.

By Kabirian-based optinalysis (i.e., Eq. (6)), each element functions as in Eq. (6.1)**–**(6.7).

(6) $$K_{c}\left(A,B\right)=\frac{4\left(x_{1}+x_{2}+x_{3}+\;\delta +y_{3}+y_{2}+y_{1}\right)}{x_{1}+2x_{2}+3x_{3}+4\delta +5y_{3}+6y_{2}+7y_{1}}$$

(6.1) $$x_{1}=\frac{K_{c}\left(2x_{2}+3x_{3}+\;4\delta +5y_{3}+6y_{2}+7y_{1}\right)-4\left(x_{2}+x_{3}+\;\delta +y_{3}+y_{2}+y_{1}\right)}{4-K_{c}}$$

(6.2) $$x_{2}=\frac{K_{c}\left(x_{1}+3x_{3}+\;4\delta +5y_{3}+6y_{2}+7y_{1}\right)-4\left(x_{1}+x_{3}+\;\delta +y_{3}+y_{2}+y_{1}\right)}{4-2K_{c}}$$

(6.3) $$x_{3}=\frac{K_{c}\left(x_{1}+2x_{2}+\;4\delta +5y_{3}+6y_{2}+7y_{1}\right)-4\left(x_{1}+x_{2}+\;\delta +y_{3}+y_{2}+y_{1}\right)}{4-3K_{c}}$$

(6.4) $$\delta =\frac{K_{c}\left(x_{1}+2x_{2}+3x_{3}+5y_{3}+6y_{2}+7y_{1}\right)-4\left(x_{1}+x_{2}+x_{3}+y_{3}+y_{2}+y_{1}\right)}{4-4K_{c}}$$

(6.5) $$y_{3}=\frac{K_{c}\left(x_{1}+2x_{2}+3x_{3}+\;4\delta +6y_{2}+7y_{1}\right)-4\left(x_{1}+x_{2}+x_{3}+\;\delta +y_{2}+y_{1}\right)}{4-5K_{c}}$$

(6.6) $$y_{2}=\frac{K_{c}\left(x_{1}+2x_{2}+3x_{3}+\;4\delta +5y_{3}+7y_{1}\right)-4\left(x_{1}+x_{2}+x_{3}+\;\delta +y_{3}+y_{1}\right)}{4-6K_{c}}$$

(6.7) $$y_{1}=\frac{K_{c}\left(x_{1}+2x_{2}+3x_{3}+\;4\delta +5y_{3}+6y_{2}\right)-4\left(x_{1}+x_{2}+x_{3}+\;\delta +y_{3}+y_{2}\right)}{4-7K_{c}}$$

Recall the definition of bijective mapping (*one-to-one and onto*), such that if x=y, then f(g(x))=g(f(y)). To verify that the x and y are bijective, three (3) cases of mathematical proves were evaluated (see details of the proves in Appendix B of the supplementary material). Based on the analysis of the proven cases, it is concluded that Kabirian-based isomorphic optinalysis ($Opt(A,A^{\prime })$) is a construction and function that satisfied the bijective mapping which signifies isomorphism of defined mathematical structures. It was interesting to have verified that each pair of isoreflective points are bijective with a different optinalysis-derived function. That means the bijectivity of one pair-points is independent of the others.

Theorem 3 *(Transformation invariance of completeness)*

Theorem: Let $A=(x_{1},x_{2},\;x_{3},\ldots ,x_{n})$ and its mirror image $A^{\prime }=\left(x{{}^{\prime }}_{1},x{{}^{\prime }}_{{}^{\prime }2},\;x{{}^{\prime }}_{3},\ldots ,x{{}^{\prime }}_{n}\right)$ be a mathematical structure. Let $A=(x_{1},x_{2},\;x_{3},\ldots ,x_{n})$ and $B=(x_{1},x_{2},\;x_{3},\ldots ,x_{n})$ be two mathematical structures. If under Kabirian-based automorphic optinalysis ($\mathrm{Opt}(A,A^{\text{'}})$), or Kabirian-based isomorphic optinalysis (Opt(A,B)); and A and $A^{\prime }$ are completely symmetrical/identical, or A and B are completely similar/identical; then the estimates are invariant to transformations such as pericentral rotation (alternate reflection), central rotation (inversion), translation (location shift), scaling, and central modulation.

The theorem proves 3 was described in the Appendix C of the supplementary material.

Theorem 4 *(Transformation invariance of incompleteness)*

Theorem: Let $A=(x_{1},x_{2},\;x_{3},\ldots ,x_{n})$ and its mirror image $A^{\prime }=\left(x{{}^{\prime }}_{1},x{{}^{\prime }}_{{}^{\prime }2},\;x{{}^{\prime }}_{3},\ldots ,x{{}^{\prime }}_{n}\right)$ be a mathematical structure. Let $A=(x_{1},x_{2},\;x_{3},\ldots ,x_{n})$ and $B=(x_{1},x_{2},\;x_{3},\ldots ,x_{n})$ be two mathematical structures. If under Kabirian-based automorphic optinalysis ($\mathrm{Opt}(A,A^{\text{'}})$), or Kabirian-based isomorphic optinalysis (Opt(A,B)); and A and $A^{\prime }$ are incompletely symmetrical/identical, or A and B are incompletely similar/identical; then the estimates are invariant to transformations such as product translation and central rotation (inversion).

The theorem proves 4 was described in the **Appendix D** of the supplementary material.

Theorem 5 *(Optinalytic normalization)*

Theorem: Let $A=(x_{1},x_{2},\;x_{3},\ldots ,x_{n})$ and its mirror image $A^{\prime }=\left(x{{}^{\prime }}_{1},x{{}^{\prime }}_{{}^{\prime }2},\;x{{}^{\prime }}_{3},\ldots ,x{{}^{\prime }}_{n}\right)$ be a mathematical structure. Let $A=(x_{1},x_{2},\;x_{3},\ldots ,x_{n})$ and $B=(x_{1},x_{2},\;x_{3},\ldots ,x_{n})$ be two mathematical structures. Let central modulation be δ+β. If under Kabirian-based automorphic optinalysis ($\mathrm{Opt}(A,A^{\text{'}})$), or Kabirian-based isomorphic optinalysis (Opt(A,B)); and A and $A^{\prime }$ are incompletely symmetrical/identical, or A and B are incompletely similar/identical; then the estimates of the optinalysis can be neutralized to near-completeness through central modulation at the central point that connected the mathematical structure(s).

The theorem proves 5 was described in the Appendix E of the supplementary material.

## Kabirian-based optinalysis-to-probability translation models (KBO-P TMs)

### Principle of the translation models

The translation of the Kabirian coefficient of optinalysis into a probability model cannot be accomplished using existing probability rules or theorems such as the product and addition rules of frequency-based probability or Bayesian probability. This is due to the unique nature of the optinalysis approach, which relies on independent, mutually inclusive, and multidimensional patterns of events. The multidimensional pattern adds complexity to the analysis, necessitating special considerations. In this probability model, the focus goes beyond determining the likelihood from the sample space; it also assesses the proximity or distance of observed events to the expected event.

The practical implications and applications of this probability model can be observed in scenarios such as unlocking a password. While there is only one chance in the sample space to unlock the password, each trial from the sample space carries a specific probability of being closer or more distant from the true password. This translation model captures and expresses this probability of proximity or distance of the observed event (i.e., for instance, the random trial of getting the true password) to the expected event (i.e., for instance, the true password).

Definition 14 Kabirian-based optinalysis-to-probability translation models

The Kabirian-based optinalysis-to-probability translation models are bridges that connect the outcomes of Kabirian-based optinalysis (i.e., Kabirian bi-coefficients) to probability. The translation models translate the two possible Kabirian bi-coefficients into a probability that infers the level of certainty to which the isoreflective or autoreflective pair of mathematical structures are similar, identical, symmetrical, or otherwise.

Theorem 6 *Kabirian-based optinalysis-to-probability translation models.*

Theorem: Let $P_{Sym./Id.}$ be the unknown actual probability of symmetry, or identity of a mathematical structure $X=(x_{1},x_{2},\;x_{3},\ldots ,x_{n})$ and its mirror image $X^{\prime }=\left(x{{}^{\prime }}_{1},x{{}^{\prime }}_{2},\;x{{}^{\prime }}_{3},\ldots ,x{{}^{\prime }}_{n}\right)$. Let $P_{Sim./Id.}$ be the unknown actual probability of similarity, or identity between two mathematical structures $X=(x_{1},x_{2},\;x_{3},\ldots ,x_{n})$ and$Y=(y_{1},y_{2},\;y_{3},\ldots ,y_{n})$ with n number of independent, mutually inclusive, and multidimensional events. Let the used optiscale be $R=(r_{1},\ldots ,nr_{1}+r_{1},\;\ldots ,2nr_{1}+r_{1})$. Let the overall expected probability be $P_{expected}=1$. If the Kabirian coefficient of optinalysis between X and its mirror image $X^{\prime }$ or between X and Y is estimated, then it defines the unknown actual probability ($P_{Sym./Sim./Id}$) as an isoreflective pair to the expected probability ($P_{expected}$) about a central point through the predefined optiscale R.

*Prove of* Theorem 6 *:*

Phase 1: forward translation, from Kabirian bi-coefficients to probability of symmetry and similarity

Let the Kabirian-coefficient of optinalysis between $X=(x_{1},x_{2},\;x_{3},\ldots ,x_{n})$ and $Y=(y_{1},y_{2},\;y_{3},\ldots ,y_{n})$ be $K_{c}$. Let the optiscale used be $R=(r_{1},\ldots ,nr_{1}+r_{1},\;\ldots ,2nr_{1}+r_{1})$. Let the unknown actual probability of symmetry, similarity, or identity between mathematical structures X and Y be $P_{Sym./Sim./Id}$. Let the overall expected probability be $P_{expected}=1$. Let the central point be δ=0.

Let's define the isoreflectivity of the optinalytic construction between the $P_{Sym./Sim./Id}$ and $P_{expected}$ as:

Or the optinalytic construction is inversely expressed as:where $r_{1}$ is the first term of the established optiscale and n is the number of dimensions or the sample size.

Then, Kabirian coefficient ($K_{c}$) is defined as:

(7.1) $$K_{c}=\frac{\left(nr_{1}+r_{1}\right)\left(P_{Sym./Sim./Id}+1\right)}{\left(r_{1}\times P_{Sym./Sim./Id}\right)+\left(2nr_{1}+r_{1}\right)}=K_{cx}$$

Or the Kabirian coefficient ($K_{c}$) is inversely defined as:

(7.2) $$K_{c}=\frac{\left(nr_{1}+r_{1}\right)\left(1+P_{Sym./Sim./Id}\right)}{r_{1}+P_{Sym./Sim./Id}\left(2nr_{1}+r_{1}\right)}=K_{cx}$$

By making $P_{Sym./Sim./Id}$ the subject of the formula from Eqs. (7.1) and (7.2), we obtain and define the models in Eqs. (7.3) and (7.4) respectively. These Eqs. (7.3) and (7.4) translate the obtained Kabirian bi-coefficients of symmetry, similarity, and identity to the probability of symmetry, similarity, and identity respectively.

(7.3) $$\begin{array}{c} P_{\mathrm{Sym}./\mathrm{Sim}./\mathrm{Id}.}\left(A,B\;\mathrm{or}\;A,A^{\text{'}}\right)=\frac{\left(nr_{1}+r_{1}\right)-K_{c}\left(2nr_{1}+r_{1}\right)}{r_{1}K_{c}-\left(nr_{1}+r_{1}\right)},\;\forall \;0\leq K_{c}\leq 1 \end{array}$$

$$\left\{ \begin{array}{c} if\;\frac{n+1}{2n+1}\leq \;KC_{Sym./Sim./Id}\left(A,B\;or\;A,A^{\prime }\right)\leq 1;\;\;\;then\;\;\;0\leq P_{Sym./Sim./Id.}\left(A,B\;or\;A,A^{\prime }\right)\leq 1\\ if\;0\leq \;KC_{Sym./Sim./Id}\left(A,B\;or\;A,A^{\prime }\right)\leq \frac{n+1}{2n+1};\;\;\;then\;\;\;-1\leq P_{Sym./Sim./Id.}\left(A,B\;or\;A,A^{\prime }\right)\leq 0 \end{array}\right.$$

Or inversely as:

(7.4) $$\begin{array}{c} P_{\mathrm{Sym}./\mathrm{Sim}./\mathrm{Id}.}\left(A,B\;\mathrm{or}\;A,A^{\text{'}}\right)=\frac{\left(nr_{1}+r_{1}\right)-r_{1}K_{c}}{K_{c}\left(2nr_{1}+r_{1}\right)-\left(nr_{1}+r_{1}\right)},\;\forall \;1\leq K_{c}\leq n+1;K_{c}\geq n+1;\;\&K_{c}\leq 0 \end{array}$$

$$\left\{ \begin{array}{c} if\;1\leq \;KC_{Sym./Sim./Id}\left(A,B\;or\;A,A^{\prime }\right)\leq n+1;\;\;\;then\;\;\;0\leq P_{Sym./Sim./Id.}\left(A,B\;or\;A,A^{\prime }\right)\leq 1\\ if\;KC_{Sym./Sim./Id}\left(A,B\;or\;A,A^{\prime }\right)\geq n+1,\;or\;\leq 0;\;\;\;then\;\;\;-1\leq P_{Sym./Sim./Id}\left(A,B\;or\;A,A^{\prime }\right)\leq 0 \end{array}\right.$$

Phase 2: Forward translation, from the probability of symmetry, similarity, and identity to the probability of asymmetry, dissimilarity, and unidentity

Eqs. (8) and (9) translate forward the probability of symmetry, similarity, and identity ($P_{Sym./Sim./Id.}$) to the probability of asymmetry, dissimilarity, and unidentity ($P_{Asym./Dsim./Uid.}$) between isoreflective or autoreflective pair of mathematical structures under Kabirian-based optinalysis. Translation of the Kabirian coefficient is valid if and only if the outcomes are within the range of values −1 to 1 (or −100 to 100 of its equivalent percentage).

If $P_{Sym./Sim./Id}\left(A,B\;or\;A,A^{\prime }\right)\geq 0$, then

(8) $$P_{Asym./Dsim.\;/Uid.}\left(A,B\;or\;A,A^{\prime }\right)=1-P_{Sym./Sim./Id}\left(A,B\;or\;A,A^{\prime }\right)$$

If $P_{Sym./Sim./Id}\left(A,B\;or\;A,A^{\prime }\right)\leq 0$, then

(9) $$P_{Asym./Dsim.\;/Uid.}\left(A,B\;or\;A,A^{\prime }\right)=-1-P_{Sym./Sim./Id}\left(A,B\;or\;A,A^{\prime }\right)$$

Phase 3: backward translation: from the probability of asymmetry, dissimilarity, and unidentity to the probability of symmetry, similarity, and identity

Eqs. (10) and (11) translate backward the probability of asymmetry, dissimilarity, and unidentity ($P_{Asym./Dsim./Uid.}$) to the probability of symmetry, similarity, and identity ($P_{Sym./Sim./Id.}$) respectively.

If $P_{Asym./Dsim./Uid}\left(A,B\;or\;A,A^{\prime }\right)\geq 0$, then

(10) $$P_{Sym./Sim.\;/id.}\left(A,B\;or\;A,A^{\prime }\right)=1-P_{Asym./Dsim./Uid}\left(A,B\;or\;A,A^{\prime }\right)$$

If $P_{Asym./Dsim./Uid}\left(A,B\;or\;A,A^{\prime }\right)\geq 0$, then

(11) $$P_{Sym./Sim.\;/id.}\left(A,B\;or\;A,A^{\prime }\right)=-1-P_{Asym./Dsim./Uid}\left(A,B\;or\;A,A^{\prime }\right)$$

Phase 4: backward translation: from the probability of symmetry, similarity, and identity to Kabirian bi-coefficients

These Eqs. (12) and (13) translate backward the probability of symmetry, similarity, and identity outcomes to its two possible Kabirian bi-coefficients, designated as KC_Alt.1 and KC_Alt.2.

(12) $$KC\_Alt.1\left(A,B\;or\;A,A^{\prime }\right)=\frac{\left(nr_{1}+r_{1}\right)\left(P_{Sym./Sim./Id}+1\right)}{\left(r_{1}\times P_{Sym./Sim./Id}\right)+\left(2nr_{1}+r_{1}\right)},\;\forall \;0\leq K_{c}\leq 1$$

(13) $$KC\_Alt.2\left(A,B\;or\;A,A^{\prime }\right)=\frac{\left(nr_{1}+r_{1}\right)\left(1+P_{Sym./Sim./Id}\right)}{r_{1}+P_{Sym./Sim./Id}\left(2nr_{1}+r_{1}\right)},\;\forall \;1\leq K_{c}\leq n+1;K_{c}\geq n+1\;\;\&\;\forall \;K_{c}\leq 0$$

where $r_{1}$ is the first term of the established optiscale and n is the sample size/item length.

The expectation of the translation models

The expectations of this translation model (of forward and backward translations of Kabirian-based optinalytic outcomes) are described as *Y-rule* (of Kabirian-based isomorphic or automorphic optinalysis). The Y-rule demonstrated below, is a Y-shaped chain of forward and backward proceedings of Kabirian-based isomorphic or automorphic outcomes.

For Kabirian-based automorphic optinalysis ($Opt(A,A^{\prime })$)

$$\begin{array}{c} KC1_{P_{Sym./Id.}}\left(A,\;A^{\prime }\right)\\ \;\;\ldots \;\;\;\;\;⇌P_{Sym./Id.}\left(A,\;A^{\prime }\right),=P_{Sym./Id.}\left(A^{\prime },\;A\right)⇌P_{Asym./Uid.}\left(A,\;A^{\prime }\right)=P_{Asym./Uid.}\left(A^{\prime },\;A\right)\\ KC2_{P_{Sym./Id.}}\left(A^{\prime },\;A\right) \end{array}$$

For Kabirian-based isomorphic optinalysis (Opt(A,B))

$$\begin{array}{c} KC1_{P_{Sim./Id.}}\left(A,\;B\right)\\ \;\;\;\;\;\;\;\;\ldots \;\;\;\;\;\;⇌P_{Sim./Id.}\left(A,\;B\right),=P_{Sim./Id.}\left(B,A\right)⇌P_{Dsim.\;/Uid.}\left(A,\;B\right)=P_{Dsim.\;/Uid.}\left(B,A\right)\\ KC1_{P_{Sim./Id.}}\left(A,\;B\right) \end{array}$$

## Properties of Kabirian-based optinalysis

i. The coefficients of Kabirian-based optinalysis and their translated estimates follow the *Y-rule of Kabirian-based isomorphic or automorphic optinalysis*.
ii. It is invariant to transformations such as pericentral rotation (alternate reflection), central rotation (inversion), translation (location shift), scaling, and central modulation if completely symmetrical, similar, or identical mathematical structures are under Kabirian-based optinalysis. The proof can be found in **Appendix C** of the supplementary material.
iii. It is invariant to transformations such as product translation and central rotation (inversion) if incompletely symmetrical, similar, or identical mathematical structures are under Kabirian-based optinalysis. The proof can be found in **Appendix D** of the supplementary material.
iv. It neutralizes near-completeness through central modulation transformation if incompletely symmetrical, similar, or identical mathematical structures are under Kabirian-based optinalysis. Further details can be found in **Appendix E** of the supplementary material.

## Python implementation

The proposed methods of Kabirian-based isomorphic and automorphic optinalysis, computing codes were written in Python language. Get the Python codes at Abdullahi [41] or via these links: https://data.mendeley.com/datasets/gnrcj8s7fp/2. Here is the documentation of the implemented codes.

## For automorphic optinalysis

### Meta-data

• Project: Automorphic Optinalysis
• Language: Python
• Libraries Used: NumPy
• Functionality: Performs automorphic optinalysis based on input data.
• Author: Abdullahi, Kabir Bindawa
• Version: 1.1

## Code description

### Importing libraries

• The code begins by importing the necessary libraries, namely NumPy.

### Function definitions

1. *automorphic_optinalysis:* The main function of automorphic optinalysis.
  • *Input:* It takes an instruction_list, which is expected to contain a list of 2 elements:
    ○ *data:* list of numerical values from a set of real numbers.
    ○ *print_result:* Specifies which type of result to print.
2. *kc_automorphic_optinalysis:* A nested function for computing the Kabirian coefficient of symmetry (kc) during automorphic optinalysis. It calculates the kc value based on the input data.
3. *psym:* A function for translating the Kabirian coefficient (kc) to percentage symmetry (psym). It takes kc and the number of dimensions as input.
4. *pasym:* A function for translating percentage symmetry (psym) to percentage asymmetry (pasym). It takes psym as input.
5. *kc_alt, kc_alt1, kc_alt2:* Functions for translating percentage symmetry (psym) to alternative Kabirian coefficients. They take kc, psym, and the number of dimensions as input.

### Main process

• Data is extracted from the instruction_list.
• The Kabirian coefficient (kc) is computed using the kc_automorphic_optinalysis function.
• The number of dimensions is calculated based on the length of data_x.
• Various estimates are calculated, including psym, pasym, and alternative Kabirian coefficients (i.e., kc_alt1, kc_alt2, and kc_alt).

### Output

• The output result depends on the print_result parameter in the instruction_list. The following options are available:

*“print:kc”:* Prints the Kabirian coefficient (kc).

*“print:psym”:* Prints the percentage symmetry (psym).

*“print:pasym”:* Prints the percentage asymmetry (pasym).

*“print:kcalt1”:* Prints the alternative Kabirian coefficient_1 (kcalt1).

*“print:kcalt2”:* Prints the alternative Kabirian coefficient_2 (kcalt2).

*“print:kcalt”:* Prints the alternative Kabirian coefficient (kcalt).

*“print:all”:* Prints all estimates encapsulated in the all_estimates dictionary.

### Error handling

• If the print_result parameter is invalid, an error message is returned.

### How it's used for analysis

i. *Automorphic Optinalysis:* This code performs automorphic optinalysis, a specific mathematical analysis of input data.
ii. *Customizable Output:* Users can choose which result(s) to print based on their analysis needs.
iii. *Data Transformation:* The code may be useful for certain mathematical analyses and transformations of data.
iv. *Extensibility:* It can be extended to support additional analyses or coefficients.
v. Overall, this code provides a tool for automorphic optinalysis and related coefficient calculations, with flexibility in selecting which results to display.

## For isomorphic optinalysis

### Meta-data

• Project: Isomorphic Optinalysis
• Language: Python
• Libraries Used: NumPy
• Functionality: Performs isomorphic optinalysis based on input data.
• Author: Abdullahi, Kabir Bindawa
• Version: 1.1

## Code description

### Importing libraries

• The code begins by importing the necessary libraries, namely NumPy.

### Function definitions

1. *isomorphic_optinalysis*: The main function of isomorphic optinalysis.
  • *Input:* It takes an instruction_list, which is expected to contain a list of 4 elements:
    ○ *data_x:* list of numerical values from a set of real numbers.
    ○ *data_y:* list of numerical values from a set of real numbers.
    ○ *pairing:* The type of sequence alignment, either Head-to-head or Tail-to-tail.
    ○ *print_result:* specifies which type of result to print.
2. *kc_isomorphic_optinalysis:* A nested function for computing the Kabirian coefficient (kc) during isomorphic optinalysis. It calculates the kc value based on the input data and pairing type.
3. *psim:* A function for translating the Kabirian coefficient (kc) to percentage similarity (psim). It takes kc and the number of dimensions as input.
4. *pdsim:* A function for translating percentage similarity (psim) to percentage dissimilarity (pdsim). It takes psim as input.
5. *kc_alt, kc_alt1, kc_alt2:* Functions for translating percentage similarity (psim) to alternative Kabirian coefficients. They take kc, psim, and the number of dimensions as input.

### Main process

• Input data (data_x, data_y) and other parameters are extracted from the instruction_list.
• The Kabirian coefficient (kc) is computed using the kc_isomorphic_optinalysis function.
• The number of dimensions is calculated based on the length of data_x.
• Various estimates are calculated, including psim, pdsim, kc_alt1, kc_alt2, and kc_alt.
• All estimates are stored in a dictionary called all_estimates.

### Output

• The output result depends on the print_result parameter in the instruction_list. The following options are available:

*“print:kc”:* Prints the Kabirian coefficient (kc).

*“print:psim”:* Prints the percentage similarity (psim).

*“print:pdsim”:* Prints the percentage dissimilarity (pdsim).

*“print:kcalt1”:* Prints the alternative Kabirian coefficient_1 (kcalt1).

*“print:kcalt2”:* Prints the alternative Kabirian coefficient_2 (kcalt2).

*“print:kcalt”:* Prints the alternative Kabirian coefficient (kcalt).

*“print:all”:* Prints all estimates encapsulated in the all_estimates dictionary.

### Error handling

• If the print_result parameter is invalid, an error message is returned.

### How it's used for analysis

i. *Isomorphic Optinalysis:* This code performs isomorphic optinalysis, a specific mathematical analysis on two input datasets.
ii. *Customizable Output:* Users can choose which result(s) to print based on their analysis needs.
iii. *Data Transformation:* The code may be useful for certain mathematical analyses and transformations on two datasets.
iv. *Extensibility:* It can be extended to support additional analyses or coefficients.
v. Overall, this code provides a tool for isomorphic optinalysis and related coefficient calculations, with flexibility in selecting which results to display.

## Drawbacks and limitations of Kabirian-based optinalysis

The following are some of the identified drawbacks and limitations of Kabirian-based optinalysis.

i. It is sensitive to the order of elements in an isoreflective or autoreflective pair of mathematical structures. Therefore, the order must be established either empirically or theoretically (e.g., ascending or descending sorting).
ii. It strictly relies on linear bijective mapping. Consequently, the length of elements in an isoreflective or autoreflective pair must be the same. If not, a suitable method should be employed to align them.
iii. For Kabirian-based isomorphic optinalysis, a suitable and efficient pairing style or alternate reflection must be selected and adopted to ensure repeatability and facilitate result comparison.
iv. The two possible Kabirian bi-coefficients do not operate on the same optinalytic scale. To compare results, the estimates with mixed Kabirian coefficients should either be translated forward or standardized through a backward alternate translation. (Refer to the Y-rule of Kabirian-based isomorphic or automorphic optinalysis).

### Method application and validation

There have been some developed working applications of Kabirian-based optinalysis [42] that proposed other methods/estimators which are too lengthy to address in a single article paper. In this paper, a geometrical pairwise analysis of biological sequences is proposed, demonstrated, and validated. The proposed method was validated and compared with a well-known metric used for pairwise analysis of biological sequences.

### Geometrical pairwise comparison of biological sequences

Geometrical pairwise comparison of biological sequences, following Kabirian-based optinalysis looks at two biological sequences (i.e., DNA, RNA, and amino acid sequences) as isoreflective as a mirror-like reflection of each other about a central point. It estimates the percentage similarity between two sequences.

In the context of biological sequence analysis, geometrical pairwise comparison refers to the empirical ordering, sequence alignment, numerical encoding, and finally optinalysing of the established isoreflective pair for the given sequences.

## Computational steps and algorithmic procedure

Suppose we have two biological sequences $X{=}^{\text{'}}\mathrm{CTA}\cdots T\text{'}$ and $\;Y{=}^{\prime }CGAT\ldots G^{\prime }$. The geometrical pairwise comparison of biological sequences, following Kabirian-based optinalysis, implies the following phases and steps:

### Preprocessing/pre-optinalysis phase

Let the order of algorithmic preprocessing treatments $t_{o}$, $t_{a}$ and $t_{e}$ as ordering, sequence alignment, and numerical encoding of the sequences X and Y respectively.

*Step 1:* Empirical ordering: establish an empirical order for the sequences X and Y. The empirical ordering is the observed order that directs transcription, translation, or protein synthesis. The experimental sequence order is the empirical order.
  $$t_{o}\left(X\right)=\left(C_{1},T_{2},A_{3},\ldots ,T_{n}\right)$$
  $$t_{o}\left(Y\right)=\left(C_{1},G_{2},A_{3},T_{4},\ldots ,G_{n}\right)$$
*Step 2:* Sequence alignment: where applicable, sequence-align the sequences $t_{o}\left(X\right)$ and $t_{o}\left(Y\right)$, using a dash symbol to represent the indels. Sequence alignment can be achieved by the traditional methods of global or local sequence alignment algorithms (such as Needle, Stretcher, Water, Matcher, MAFFT, MUSCLE, Clustal Omega, T-Coffee, etc.). The sequence alignment ensured that the isoreflective pairs had the same sequence length.
  $$t_{o\to a}\left(X\right)=\left(C_{1},T_{2},A_{3},{-}_{4},\ldots ,T_{n}\right)$$
  $$t_{o\to a}\left(Y\right)=\left(C_{1},G_{2},A_{3},T_{4},\ldots ,G_{n}\right)$$
*Step 3:* Numerical encoding: convert each biological sequence (i.e., $t_{o\to a}\left(X\right)$ and $t_{o\to a}\left(Y\right)$) into a numerical representation using a specific encoding scheme. This encoding captures the features of the sequences such as base or amino acid compositions, and aligned gabs of the sequences $t_{o\to a}\left(X\right)$ and $t_{o\to a}\left(Y\right)$. In this paper, two numerical encoding schemes (i.e., DNA and protein-encoding schemes) were provided based on the molecular mass representation of each biological letter of the biological sequences (Tables 4 and 5).

**Table 4:** DNA encoding scheme based on the molecular mass of the nucleic acids.

DNA/RNA bases and symbol	1-letter code	Molecular weight (g/mol)
All other gabs	–	0
Cytosine	C	111
Tymine	T	126
Adenine	A	135
Guinine	G	151
Uracil	U	112

**Table 5:** Protein encoding scheme based on the molecular mass of amino acids.

Amino Acid	3-letter Code	1-letter Code	Molecular weight (g/mol)
All other gabs		–	0
Glycine	Gly	G	75
Alanine	Ala	A	89
Serine	Ser	S	105
Proline	Pro	P	115
Valine	Val	V	117
Threonine	Thr	T	119
Cysteine	Cys	C	121
Isoleucine	Ile	I	131
Leucine	Leu	L	131
Asparagine	Asn	N	132
Aspartate	Asp	D	133
Glutamine	Gln	Q	146
Lysine	Lys	K	146
Glutamate	Glu	E	147
Methionine	Met	M	149
Histidine	His	H	155
Phenylalanine	Phe	F	165
Arginine	Arg	R	174
Tyrosine	Tyr	Y	181
Tryptophan	Trp	W	204

The aligned biological sequences $t_{o\to a}\left(X\right)$ and $t_{o\to a}\left(Y\right)$ now becomes:

$$t_{o\to a\to e}\left(X\right)=\left(111_{1},126_{2},135_{3},0_{4},\ldots ,126_{n}\right)$$

$$t_{o\to a\to e}\left(Y\right)=\left(111_{1},151_{2},135_{2},126_{2},\ldots ,151_{n}\right)$$

### Optinalysis phase

*Step 4:* Choose an efficient pairing style (reflection) and establish the isoreflective pair between $t_{o\to a\to e}\left(X\right)$ and $t_{o\to a\to e}\left(Y\right)$ about δ. For instance,

Head-to-head pairing or reflection is given as:

Tail-to-tail pairing or reflection is given as:

For DNA, RNA, and amino acid sequences, it is recommended to use head-to-head pairing of biological sequences because they are typically presented and read in the $5^{\prime }$ to $3^{\prime }$ direction, This directionality ensures the proper progression and coordination of essential cellular processes. It allows for the accurate transcription of DNA into RNA and the correct translation of mRNA into proteins. However, tail-to-tail pairing can be used where applicable.

*Step 5:* Optinalysis: Optinalyse by Kabirian-based optinalysis the isoreflective pair of  or  about a mid-point δ. In this representation, the head-to-head pairing was used.

Such that δ∉X,Y;$R\subseteq \{r\in R^{+*}|r=n*k,\;n\in N,\;k>0\}$ or alternatively $R\subseteq \{r\in R^{-*}|r=-n*k,\;n\in N,\;k>0\}$; n∈N; and X&Y are isoreflective pairs. δ=0 is by default operation, except under optinalytic normalization.

By Kabirian-based optinalysis, the Kabirian coefficient of similarity $\left(KC_{Gsim.}\right)$, percentage similarity, percentage dissimilarity, and others satisfy the Y-rule of Kabirian-based isomorphic optinalysis (Opt(X,Y)) be:

$$\begin{array}{c} KC1_{Gsim.}\left(X,Y\right)\\ \;\;\ldots \;\;\;\;⇌P_{Gsim.}\left(X,Y\right)=P_{Gsim.}\left(Y,X\right)⇌P_{Gdsim.}\left(X,Y\right)=P_{Gdsim.}\left(Y,X\right)\\ KC2_{Gsim.}\left(Y,X\right) \end{array}$$

where X and Y are the preprocessed biological sequences

The two possible Kabirian bi-coefficients $\left(\mathrm{KC}1_{\mathrm{Gsim}.}\&\;\mathrm{KC}2_{\mathrm{Gsim}.}\right)$ function on two different, but inverse optinalytic scales.

## Python implementation

Get the Python code at Abdullahi [43] or via this link: (https://data.mendeley.com/datasets/tnwpt54jnb/2). Here is the documentation of the code:

### Meta-data

• Project: Isomorphic Optinalysis
• Language: Python
• Libraries Used: Numpy
• Functionality: Perform sequence comparison and similarity analysis on DNA, RNA, or Protein sequences.
• Author: Abdullahi, Kabir Bindawa
• Version: 1.1

## Code description

### Importing libraries

• The code starts by importing the Numpy library, useful for numerical computations.

### Function definitions

1. *pairwise_analysis:* This is the main function where the whole process takes place.
  • *Input:* takes an instruction_list, which is expected to contain a list of 5 elements:
    ○ *sequence_x and sequence_y:* DNA, RNA, or Protein sequences for comparison.
    ○ *encoding_scheme:* The type of encoding to be applied ('seq_type:DNA', 'seq_type:RNA', or 'seq_type:protein').
    ○ *pairing:* The type of sequence alignment, either Head-to-head or Tail-to-tail.
    ○ *print_result:* Specifies which type of result to print.
2. *DNA_encoding, RNA_encoding, protein_encoding:* These functions convert sequences into numerical values based on a predefined encoding scheme.
3. *kc_isomorphic_optinalysis:* Calculates the Kabirian coefficient of similarity based on the provided sequences and the type of pairing.
4. *psim:* A function for translating the Kabirian coefficient (kc) to percentage similarity (psim). It takes kc and the number of dimensions as input.
5. *pasim:* A function for translating percentage similarity (psim) to percentage dissimilarity (pdsim). It takes psim as input.
6. *kc_alt, kc_alt1, kc_alt2:* Functions for translating percentage similarity (psim) to alternative Kabirian coefficients. They take kc, psim, and the number of dimensions as input.

### Main process

• Input data (sequence_x and sequence_y) and other parameters are extracted from the instruction_list.
• The sequence type is identified and encoded accordingly.
• The Kabirian coefficient (kc) is computed using the kc_isomorphic_optinalysis function.
• Various estimates are calculated, including psim, pdsim, and alternative Kabirian coefficients (i.e., kc_alt1, kc_alt2, and kc_alt).

### Output

• The output result depends on the print_result parameter in the instruction_list. The following options are available:

*“print:kc”:* Prints the Kabirian coefficient (kc).

*“print:psim”:* Prints the percentage similarity (psim).

*“print:pdsim”:* Prints the percentage dissimilarity (pdsim).

*“print:kcalt1”:* Prints the alternative Kabirian coefficient_1 (kcalt1).

*“print:kcalt2”:* Prints the alternative Kabirian coefficient_2 (kcalt2).

*“print:kcalt”:* Prints the alternative Kabirian coefficient (kcalt).

*“print:all”:* Prints all estimates encapsulated in the all_estimates dictionary.

### Error handling

• The code includes several checks for invalid input for the pairing, encoding_scheme, and print_result parameters.

### How it's used for analysis

i. *Biological Sequence Comparison:* It can be used for comparing the similarity or dissimilarity between two DNA, RNA, or Protein sequences.
ii. *Data Transformation:* Converts complex biological data into numerical data that can be easily analyzed.
iii. *Multi-dimensional Analysis:* Calculates various types of coefficients and similarities, offering a multi-dimensional view of sequence similarity.
iv. *Extensible:* By changing the encoding scheme or formulae, the function can be adapted for different kinds of analyses.
v. This code provides a comprehensive tool for sequence analysis, useful in fields like bioinformatics, genetics, and computational biology.

## Manual calculations in a geometrical pairwise analysis of biological sequences

Table 6a demonstrates a manual calculation of the geometrical pairwise analysis of two DNA sequences using the proposed method.

**Table 6a:** Manual demonstration of geometrical pairwise analysis of DNA sequences.

Parameters and operations	Selection	Kabirian-based Isomorphic Optinalysis: Steps and calculations for the geometrical pairwise analysis of biological sequence
Empirically ordered observations	Aligned DNA sequences	Sequence: *A* = “ATC - TA”
		Sequence: *B* = “A - G - TA"
Numerical encoding	Ordinal encoding based on molecular masses	Sequence: *A* = [135, 126, 111, 0, 126, 135]
		Sequence: *B* = [135, 0, 151, 0, 126, 135]
Optinalytic construction and pairing style	Head-to-head (*H—H*) pairing is used but can be chosen as tail-to-tail (*T-T*) pairing, of the isoreflective pairs	Such that δ∉A,B;A,B,δ∈R; $R\subseteq \{r\in R^{+*}|r=n*k,\;n\in N,\;k>0\}$ or alternatively $R\subseteq \{r\in R^{-*}|r=-n*k,\;n\in N,\;k>0\}$; n∈N; and A&B are isoreflective pair on a chosen pairing about a central point δ.
Normalization of δ and data substitution	By default, normalization is zero, δ=0	
Calculations/computations	Numeration (the sum of variables × median optiscale)	$r_{n+1}\left[\sum_{i=1}^{n}\left(a_{i}+\delta +b_{i}\right)\right]=8260$
	Denomination (the sum of the scalements)	$\sum_{i=1}^{n}\sum_{j=n+2}^{2n+1}\left(r_{i}a_{i}+r_{n+1}\delta +r_{j}b_{i}\right)=7790$
Kabirian coefficient and optinalysis-to-probability translation translations	Kabirian coefficient of similarity	$KC_{Sim\;}\left(A,B\right)=\frac{r_{n+1}\left[\sum_{i=1}^{n}\left(a_{i}+\delta +b_{i}\right)\right]}{\sum_{i=1}^{n}\sum_{j=n+2}^{2n+1}\left(r_{i}a_{i}+r_{n+1}\delta +r_{j}b_{i}\right)}=1.060334$
	Estimate of similarity	$\forall \;0\leq K_{c}\leq 1$ $P_{\mathrm{Sim}.}\left(A,B\right)=\frac{\left(nr_{1}+r_{1}\right)-K_{c}\left(2nr_{1}+r_{1}\right)}{r_{1}K_{c}-\left(nr_{1}+r_{1}\right)}$
		$\forall \;1\leq K_{c}\leq n+1;$ $K_{c}\geq n+1\;;\;$ $\&\;K_{c}\leq 0$ $P_{\mathrm{Sim}.}\left(A,B\right)=\frac{\left(nr_{1}+r_{1}\right)-r_{1}K_{c}}{K_{c}\left(2nr_{1}+r_{1}\right)-\left(nr_{1}+r_{1}\right)}=0.8755=87.55\%$
	Estimate of dissimilarity	$\forall \;P_{Sim.}\geq 0$ $P_{Dsim.\;}\left(A,B\right)=1-P_{Sim.}\left(A,B\right)=0.1245=12.45\%$
		$\forall \;P_{Sim.}\leq 0$ $P_{Dsim.\;}\left(A,B\right)=-1-P_{Sim.}\left(A,B\right)$

## Drawbacks and limitations of geometrical pairwise comparison

The following are some of the identified drawbacks and limitations of geometrical pairwise comparison following Kabirian-based optinalysis:

i. Variables lengths must be the same, otherwise, a suitable method needs to be used to align them.
ii. A suitable and efficient pairing style or alternate reflection has to be chosen and adopted for repeatability and comparison of results.

## Methods’ comparison and evaluation

### Datasets and sequence analysis

To examine the variations in biological sequences (i.e., DNA, and protein sequences), well-structured sequences were generated, encompassing position-specific and character-specific matches, mismatches, and deletions. The alignment of these sequences was assumed to have been performed using any suitable alignment tools (such as Needle, Stretcher, Water, and Matcher algorithms), ensuring the influence of alignment methods is eliminated. For comparative analysis, pairwise comparison was conducted using two approaches: the conventional percentage similarity metric (Eq. (14)) and a proposed method of geometrical pairwise sequence comparison.

(14) $$\%\;similarity=\frac{Number\;of\;Matches}{Total\;Aligned\;Positions\;or\;Length\;of\;the\;Longer\;Sequence}\times 100$$

This (Eq. (14)) formula provides a measure of similarity based on the proportion of identical positions between the sequences. It is widely used in bioinformatics and sequence analysis to quantify the degree of similarity or conservation between biological sequences, such as DNA, RNA, or protein sequences.

The percentage similarity calculations using the proposed method can be found in the resources available at Abdullahi [43] or via https://data.mendeley.com/datasets/tnwpt54jnb/2

### Evaluation methods

To calculate the sensitivity of the two methods in detecting variations in datasets (of the well-structured sequences), entropy was used to evaluate the results obtained by each method following a pairwise comparison of the reference sequence with all the variant sequences bearing the same patterns of variation. Sensitivity, in this context, refers to the ability of a method to detect and respond to variations in the datasets.

Entropy: Entropy is a measure commonly used in information theory to quantify the randomness or uncertainty of a set of estimates [13]. A higher entropy indicates greater uniqueness and unpredictability in the results, suggesting that the method is more sensitive to dataset variations. Conversely, a lower entropy indicates less distinctiveness and more predictability in the results, suggesting that the method is less sensitive. Entropy is expressed in Eq. (15). The percentage relative entropy (measured as the proportion of the observed entropy in a method to the overall expected entropy) is expressed in Eq. (16).

(15) $$Entropy=-\sum P\left(x\right)\times log_{2}\left(P\left(x\right)\right)$$

(16) $$\%\;relative\;entropy=-\frac{Entropy}{log_{2}\left(\frac{1}{n}\right)}\times 100$$

In this equation, P(x) represents the probability of occurrence for each unique value or category x in a set of estimates, $log_{2}\left(\frac{1}{n}\right)$ always represents the overall expected entropy when all the estimates are unique, n represents the estimates sample size. The logarithm is usually taken to base 2 to represent the entropy in bits.

## Results and Discussions

 

## Results

### Kabirian-based optinalysis

In this study, a novel approach called Kabirian-based optinalysis was introduced, presenting a paradigm shift in estimating symmetry/asymmetry, similarity/dissimilarity, and identity/unidentity measures between isoreflective or autoreflective pairs of mathematical structures. The method is conceptually and theoretically grounded in the principles of automorphism and isomorphism.

The core concept of Kabirian-based optinalysis lies in its utilization of uniform intervals on the optiscale, which ensures an equidistant relationship (isometry) between corresponding mathematical structures. Moreover, this paper provides proof of the bijective and inverse functional nature of the optinalytic relationship between any pair of isoreflective or autoreflective mathematical structures. Importantly, the bijectivity of one pair of points is independent of the others, making Kabirian-based optinalysis a reliable method for comparing mathematical structures and establishing functional bijectivity for isomorphic or automorphic structures.

One of the key strengths of Kabirian-based optinalysis is its invariance under various mathematical operations or transformations such as scaling, rotation, and location shift. These invariance properties serve as strong evidence of the method's robustness in estimating symmetry, similarity, and identity measures.

However, it is important to acknowledge some limitations and drawbacks of Kabirian-based optinalytic measures. Firstly, the ordering of the variables in the list must be predetermined or established beforehand. Additionally, the length of the variables must be the same to ensure accurate comparisons. Lastly, the choice and adoption of pairing style or alternate reflection are factors that need to be considered when applying Kabirian-based optinalysis.

In conclusion, Kabirian-based optinalysis introduces a new approach to comparing mathematical structures, offering valuable insights into symmetry estimation, similarity, and identity measures. Its theoretical foundations, coupled with the demonstrated robustness under mathematical transformations, make it a promising tool for analyzing and establishing functional bijectivity in isomorphic or automorphic structures.

Sensitivity to position-specific match and mismatch (substitution)in biological sequence analysis

Our results demonstrate a notable disparity between the percentage similarity scores obtained using a method and our proposed method. The conventional methods used for calculating percentage similarity overlook the position of the base/amino acid mismatch within the sequence. As a result, these methods provide similar percentage similarity scores for any comparison between the reference sequence (S_0_) and the variant sequences (S_1_-S_12_) that have position-specific matches and mismatches (Table 6b). The conventional method shows 0 % sensitivity to position-specific variations in the sequences as a result of a single character mismatch.

**Table 6b:** Sensitivity to position-specific match and mismatch in biological sequence analysis.

Aligned DNA and protein sequences		Pairwise percentage similarity between the reference DNA or protein sequences and the variant sequences.						
Reference sequence	Reference sequence	CM	Proposed methods (DNA)			Proposed methods (protein)		
		$\%\mathrm{Sim}.^{*}$	$KC_{\mathrm{Gsim}.}$	$P_{\mathrm{Gsim}.}$	$P_{\mathrm{Gdsim}.}$	$KC_{\mathrm{Gsim}.}$	$P_{\mathrm{Gsim}.}$	$P_{\mathrm{Gdsim}.}$
S_0_ = CTAGCTAGCTAG	S_0_ = GASPCLDQMFRY	**S_0_**	**S_0_**	**S_0_**	**S_0_**	**S_0_**	**S_0_**	**S_0_**
**Variant sequences**	**Variant sequences**							
S_0_ = CTAGCTAGCTAG	S_0_ = GASPCLDQMFRY	100	100	100	100	100	100	100
S_1_ = ***G***TAGCTAGCTAG	S_1_ = ***W***ASPCLDQMFRY	91.67	1.011755	97.51	2.49	1.037470	92.47	7.53
S_2_ = C***A***AGCTAGCTAG	S_2_ = G***W***SPCLDQMFRY	91.67	1.002426	99.48	0.52	1.030545	93.78	6.22
S_3_ = CT***T***GCTAGCTAG	S_3_ = GA***W***PCLDQMFRY	91.67	0.997792	99.52	0.48	1.023866	95.07	4.93
S_4_ = CTA***C***CTAGCTAG	S_4_ = GAS***W***CLDQMFRY	91.67	0.991140	98.08	1.92	1.019283	95.98	4.02
S_5_ = CTAG***G***TAGCTAG	S_5_ = GASP***W***LDQMFRY	91.67	1.007806	98.34	1.66	1.015962	96.65	3.35
S_6_ = CTAGC***A***AGCTAG	S_6_ = GASPC***W***DQMFRY	91.67	1.001542	99.67	0.33	1.012277	97.41	2.59
S_7_ = CTAGCT***T***GCTAG	S_7_ = GASPCL***W***QMFRY	91.67	0.998674	99.71	0.29	1.010220	97.83	2.17
S_8_ = CTAGCTA***C***CTAG	S_8_ = GASPCLD***W***MFRY	91.67	0.995059	98.93	1.07	1.006963	98.51	1.49
S_9_ = CTAGCTAG***G***TAG	S_9_ = GASPCLDQ***W***FRY	91.67	1.003888	99.16	0.84	1.005278	98.87	1.13
S_10_ = CTAGCTAGC***A***AG	S_10_ = GASPCLDQM***W***RY	91.67	1.000660	99.86	0.14	1.002814	99.39	0.61
S_11_ = CTAGCTAGCT***T***G	S_11_ = GASPCLDQMF***W***Y	91.67	0.999558	99.90	0.10	1.001445	99.69	0.31
S_12_ = CTAGCTAGCTA***C***	S_12_ = GASPCLDQMFR***W***	91.67	0.999008	99.79	0.21	1.000555	99.88	0.12
Sensitivity (entropy) for S_1_**-**S_12_		0	3.58	3.58	3.58	3.58	3.58	3.58
% relative sensitivity (entropy) for S_1_**-**S_12_		0	100	100	100	100	100	100

Keys: CM = Conventional method; $\%\;\mathrm{Sim}.^{*}$ = Percentage similarity in DNA and Protein sequences; $KC_{\mathrm{Gsim}.}$ = Kabirian-coefficient of geometrical similarity; $P_{\mathrm{Gsim}.}$ = Percentage geometrical similarity; $P_{\mathrm{Gdsim}.}$ = Percentage geometrical dissimilarity.

Note: Each variant sequence differs from the reference sequence by one position-specific mismatch of character. The bolded and italicized character(s) indicate the points of variations.

On the other hand, the proposed method takes into account the position of the base/amino acid mismatch. The results (Table 6b) obtained from the proposed method show that the percentage similarity scores increase as the position of the base/amino acid mismatch moves towards the right terminal end of the sequence (left-to-right direction). This means that the proposed method is more sensitive to the location of the mismatch, and a mismatch closer to the right terminal end contributes to a higher similarity score. The proposed method shows 100 % sensitivity to position-specific variations in the sequences as a result of single character mismatch.

These findings underscore the improved sensitivity and discriminatory capabilities of the proposed method in capturing the effects of position-specific mismatches within the sequence. By considering the location of character matches and mismatches, the proposed method provides a more nuanced and accurate assessment of sequence similarity.

Sensitivity to character-specific match and mismatch (substitution)in biological sequence analysis

In the context of sequence analysis, the conventional methods utilized in this study exhibit similar percentage similarity scores when comparing the reference sequence (S_0_) to variant sequences (S_13_-S_21_) with character-specific matches and mismatches (Table 7). These mismatches can arise from transitional and transversional alterations in DNA sequences or isobaric and non-isobaric alterations in protein sequences. The conventional method shows 0 % sensitivity to character-specific variations in the sequences as a result of a single character mismatch.

**Table 7:** Sensitivity to character-specific match and mismatch in biological sequence analysis.

Aligned DNA and protein sequences		Pairwise percentage similarity between the reference DNA or protein sequences and the variant sequences.						
Reference sequence	Reference sequence	CM	Proposed methods (for DNA analysis)			Proposed methods (for protein analysis)		
		$\%\mathrm{Sim}.^{*}$	$KC_{\mathrm{Gsim}.}$	$P_{\mathrm{Gsim}.}$	$P_{\mathrm{Gdsim}.}$	$KC_{\mathrm{Gsim}.}$	$P_{\mathrm{Gsim}.}$	$P_{\mathrm{Gdsim}.}$
S_0_ = CTAGCTAGCTAG	S_0_ = GASPCLDQMFRY	**S_0_**	**S_0_**	**S_0_**	**S_0_**	**S_0_**	**S_0_**	**S_0_**
**Variant sequences**	**Variant sequences**							
S_0_ = CTAGCTAGCTAG	S_0_ = GASPCLDQMFRY	100	100	100	100	100	100	100
S_13_ = ***G***TAGCTAGCTAG	S_13_ = ***A***ASPCLDQMFRY	91.67	1.011755	97.51	2.49	1.004078	99.12	0.88
S_14_ = ***A***TAGCTAGCTAG	S_14_ = ***S***ASPCLDQMFRY	91.67	1.007056	98.49	1.51	1.008735	98.14	1.86
S_15_ = ***T***TAGCTAGCTAG	S_15_ = ***P***ASPCLDQMFRY	91.67	1.004411	99.05	0.95	1.011644	97.54	2.46
S_16_ = CTAGC***G***AGCTAG	S_16_ = GASPC***A***DQMFRY	91.67	1.004274	99.08	0.92	0.992817	98.44	1.56
S_17_ = CTAGC***A***AGCTAG	S_17_ = GASPC***S***DQMFRY	91.67	1.001542	99.67	0.33	0.995564	99.04	0.96
S_18_ = CTAGC***C***AGCTAG	S_18_ = GASPC***P***DQMFRY	91.67	0.997420	99.44	0.56	0.997274	99.41	0.59
S_19_ = CTAGCTAGCT***G***G	S_19_ = GASPCLDQMF***A***Y	91.67	1.000781	99.83	0.17	0.995776	99.09	0.91
S_20_ = CTAGCTAGCT***T***G	S_20_ = GASPCLDQMF***S***Y	91.67	0.999558	99.90	0.10	0.996586	99.26	0.74
S_21_ = CTAGCTAGCT***C***G	S_21_ = GASPCLDQMF***P***Y	91.67	0.998816	99.74	0.26	0.997089	99.37	0.63
Sensitivity (entropy) for S_13_**-**S_21_		0	3.17	3.17	3.17	3.17	3.17	3.17
% relative sensitivity (entropy) for S_13_**-**S_21_		0	100	100	100	100	100	100

Keys: CM = Conventional method; $\%\;\mathrm{Sim}.^{*}$ = Percentage similarity in DNA and Protein sequences; $KC_{\mathrm{Gsim}.}$ = Kabirian-coefficient of geometrical similarity; $P_{\mathrm{Gsim}.}$ = Percentage geometrical similarity; $P_{\mathrm{Gdsim}.}$ = Percentage geometrical dissimilarity.

Note: Each variant sequence differs from the reference sequence by one character-specific mismatch in a point. The bolded and italicized characters(s) indicate the points of variations.

In contrast, the proposed method introduces variability in the percentage similarity scores obtained from these comparisons (Table 7). Analyzing DNA sequences reveals that higher similarity scores are observed when the character mismatch involves transitional alterations, specifically changes between purines or between pyrimidines, and when the mismatch is located close to the right terminal end of the sequence. Conversely, lower similarity scores are observed when the mismatch involves transversional alterations, such as changes between purines and pyrimidines, and when it is situated towards the left terminal end. These findings indicate that the proposed method exhibits enhanced sensitivity to transitional mismatches, particularly when they occur towards the right terminal end of the sequence. The proposed method shows 100 % sensitivity to character-specific variations in the DNA sequences as a result of single base mismatch.

Similarly, when comparing protein sequences, the results (Table 7) demonstrate higher similarity scores for character mismatches that are isobaric, involving substitutions with amino acids of equal or nearly equal molecular masses, and located close to the right terminal end. Conversely, lower similarity scores are observed for non-isobaric mismatches, where substitutions involve amino acids with different or widely different molecular masses, and when these mismatches are located towards the left terminal end. This suggests that the proposed method displays greater sensitivity to non-isobaric mismatches, especially when they occur further toward the left terminal end of the sequence. The proposed method shows 100 % sensitivity to character-specific variations in the protein sequences as a result of single amino acid mismatch.

These findings underscore the improved sensitivity and discriminatory capabilities of the proposed method in capturing the effects of specific types of mismatches based on their position within the sequence. By considering the location and nature of character matches and mismatches, the proposed method provides a more nuanced and accurate assessment of sequence similarity.

### Sensitivity to position-specific deletion in biological sequence analysis

For comparisons between the reference sequence (S_0_) and the variant sequences (S_22_-S_33_) that have position-specific deletions, the conventional methods provide similar percentage similarity scores (Table 8). These methods do not consider the position of the deleted base/amino acid, resulting in consistent similarity scores. The conventional method shows 0 % sensitivity to position-specific variations in the sequences as a result of single character deletion.

**Table 8:** Sensitivity to position-specific deletion in biological sequence analysis.

Aligned DNA and protein sequences		Pairwise percentage similarity between the reference DNA or protein sequences and the variant sequences.						
Reference sequence	Reference sequence	CM	Proposed methods (for DNA analysis)			Proposed methods (for protein analysis)		
		$\%\mathrm{Sim}.^{*}$	$KC_{\mathrm{Gsim}.}$	$P_{\mathrm{Gsim}.}$	$P_{\mathrm{Gdsim}.}$	$KC_{\mathrm{Gsim}.}$	$P_{\mathrm{Gsim}.}$	$P_{\mathrm{Gdsim}.}$
S_0_ = CTAGCTAGCTAG	S_0_ = GASPCLDQMFRY	**S_0_**	**S_0_**	**S_0_**	**S_0_**	**S_0_**	**S_0_**	**S_0_**
**Variant sequences**	**Variant sequences**							
S_0_ = CTAGCTAGCTAG	S_0_ = GASPCLDQMFRY	100	100	100	100	100	100	100
S_22_ = ***-*** TAGCTAGCTAG	S_22_ = ***-*** ASPCLDQMFRY	91.67	0.967259	92.93	7.07	0.978107	95.27	4.73
S_23_ = C ***-*** AGCTAGCTAG	S_23_ = G***-*** SPCLDQMFRY	91.67	0.965813	92.61	7.39	0.976125	94.84	5.16
S_24_ = CT ***-*** GCTAGCTAG	S_24_ = GA ***-*** PCLDQMFRY	91.67	0.966575	92.78	7.22	0.974308	94.45	5.55
S_25_ = CTA ***-*** CTAGCTAG	S_25_ = GAS***-*** CLDQMFRY	91.67	0.966186	92.69	7.31	0.974585	94.51	5.49
S_26_ = CTAG ***-*** TAGCTAG	S_26_ = GASP***-*** LDQMFRY	91.67	0.977932	95.23	4.77	0.976145	94.84	5.16
S_27_ = CTAGC ***-*** AGCTAG	S_27_ = GASPC***-*** DQMFRY	91.67	0.977971	95.24	4.76	0.977301	95.09	4.91
S_28_ = CTAGCT ***-*** GCTAG	S_28_ = GASPCL***-*** QMFRY	91.67	0.979673	95.60	4.40	0.980175	95.71	4.29
S_29_ = CTAGCTA***-*** CTAG	S_29_ = GASPCLD***-*** MFRY	91.67	0.980928	95.87	4.13	0.981757	96.05	3.95
S_30_ = CTAGCTA G***-*** TAG	S_30_ = GASPCLDQ***-*** FRY	91.67	0.988843	97.58	2.42	0.985041	96.76	3.24
S_31_ = CTAGCTAGC ***-*** AG	S_31_ = GASPCLDQM***-*** RY	91.67	0.990439	97.93	2.07	0.987479	97.29	2.71
S_32_ = CTAGCTAGCT ***-*** G	S_32_ = GASPCLDQMF ***-*** Y	91.67	0.993131	98.51	1.49	0.991138	98.08	1.92
S_33_ = CTAGCTAGCTA ***-***	S_33_ = GASPCLDQMFR ***-***	91.67	0.996126	99.16	0.84	0.995360	99.00	1.00
Sensitivity (entropy) for S_22_**-**S_33_		0	3.58	3.58	3.58	3.58	3.58	3.58
% relative sensitivity (entropy) for S_22_**-**S_33_		0	100	100	100	100	100	100

Keys: CM = Conventional method; $\%\;\mathrm{Sim}.^{*}$ = Percentage similarity in DNA and Protein sequences; $KC_{\mathrm{Gsim}.}$ = Kabirian-coefficient of geometrical similarity; $P_{\mathrm{Gsim}.}$ = Percentage geometrical similarity; $P_{\mathrm{Gdsim}.}$ = Percentage geometrical dissimilarity.

Note: Each variant sequence differs from the reference sequence by one position-specific deletion of character. The bolded character(s) indicate the points of variations.

In contrast, the proposed method yields variably different percentage similarity scores for these comparisons (Table 8). The results indicate that the similarity scores increase as the position of the deleted base/amino acid moves toward the right terminal end of the sequence. This means that the proposed method is influenced by the location of the deletion, and deletions closer to the right terminal end contribute to higher similarity scores. The proposed method shows 100 % sensitivity to position-specific variations in the sequences as a result of single-character deletion.

These findings underscore the improved sensitivity and discriminatory capabilities of the proposed method in capturing the effects of position-specific deletions within the sequence. By considering the location of character deletion(s), the proposed method provides a more nuanced and accurate assessment of sequence similarity.

### Sensitivity to terminal deletions in biological sequence analysis

When considering comparisons between the reference sequence (S_0_) and the variant sequences (S_34_-S_47_) that have terminal deletions, the conventional methods provide variable similarity scores influenced by the sequence length (Table 9). As the length of the sequence reduces due to deletions, the similarity scores decrease. However, the terminal in which the deleted bases occur does not affect the similarity scores obtained from this conventional method. The conventional method shows 74 % sensitivity to terminal deletions (variations) in the sequences.

**Table 9:** Sensitivity to terminal deletions in biological sequence analysis.

Aligned DNA and protein sequences		Pairwise percentage similarity between the reference DNA or protein sequences and the variant sequences.						
Reference sequence	Reference sequence	CM	Proposed methods (for DNA analysis)			Proposed methods (for protein analysis)		
		$\%\mathrm{Sim}.^{*}$	$KC_{\mathrm{Gsim}.}$	$P_{\mathrm{Gsim}.}$	$P_{\mathrm{Gdsim}.}$	$KC_{\mathrm{Gsim}.}$	$P_{\mathrm{Gsim}.}$	$P_{\mathrm{Gdsim}.}$
S_0_ = CTAGCTAGCTAG	S_0_ = GASPCLDQMFRY	**S_0_**	**S_0_**	**S_0_**	**S_0_**	**S_0_**	**S_0_**	**S_0_**
**Variant sequences**	**Variant sequences**							
S_0_ = CTAGCTAGCTAG	S_0_ = GASPCLDQMFRY	100	100	100	100	100	100	100
S_34_ = CTAGCTAGCT ***- -***	S_34_ = GASPCLDQMF***- -***	83.33	0.988772	97.57	2.43	0.985740	96.91	3.09
S_35_ = CTAGCTAGC ***- - -***	S_35_ = GASPCLDQM ***- - -***	75.00	0.977951	95.23	4.77	0.971113	93.76	6.24
S_36_ = CTAGCTAG ***- - - -***	S_36_ = GASPCLDQ***- - - -***	66.67	0.964726	92.38	7.62	0.952502	89.75	10.25
S_37_ = CTAGCTA ***- - - - -***	S_37_ = GASPCLD ***- - - - -***	58.33	0.941287	87.34	12.66	0.928656	84.63	15.37
S_38_ = CTAGCT ***- - - - - -***	S_38_ = GASPCL ***- - - - - -***	50.00	0.915128	81.74	18.26	0.901650	78.86	21.14
S_39_ = CTAGC ***- - - - - - -***	S_39_ = GASPC ***- - - - - - -***	41.67	0.885861	75.50	24.50	0.869803	72.09	27.91
S_40_ = CTAG ***- - - - - - - -***	S_40_ = GASP ***- - - - - - - -***	33.33	0.855920	69.15	30.85	0.835614	64.86	35.14
S_41_ = ***- - - - - - - -*** CTAG	S_41_ = ***- - - - - - - -*** MFRY	33.33	0.756327	48.25	51.75	0.798805	57.13	42.87
S_42_ =***- - - - - - -*** GCTAG	S_42_ =***- - - - - - -*** QMFRY	41.67	0.784561	54.14	45.86	0.824311	62.48	37.52
S_43_ = ***- - - - - -*** AGCTAG	S_43_ = ***- - - - - -*** DQMFRY	50.00	0.811157	59.72	40.28	0.849116	67.71	32.29
S_44_ = ***- - - - -***TAGCTAG	S_44_ = ***- - - - -***LDQMFRY	58.33	0.837523	65.27	34.73	0.875300	73.26	26.74
S_45_ = ***- - - -*** CTAGCTAG	S_45_ = ***- - - -*** CLDQMFRY	66.67	0.862336	70.51	29.49	0.901285	78.79	21.21
S_46_ = ***- - -***GCTAGCTAG	S_46_ = ***- - -*** PCLDQMFRY	75.00	0.898366	78.16	21.84	0.927886	84.47	15.53
S_47_ = ***- -***AGCTAGCTAG	S_47_ = ***- -*** SPCLDQMFRY	83.33	0.932774	85.52	14.48	0.954093	90.09	9.91
Sensitivity (entropy) for S_34_**-**S_47_		2.81	3.81	3.81	3.81	3.81	3.81	3.81
% relative sensitivity (entropy) for S_34_**-**S_47_		73.75	100	100	100	100	100	100

Keys: CM = Conventional method; $\%\;\mathrm{Sim}.^{*}$ = Percentage similarity in DNA and Protein sequences; $KC_{\mathrm{Gsim}.}$ = Kabirian-coefficient of geometrical similarity; $P_{\mathrm{Gsim}.}$ = Percentage geometrical similarity; $P_{\mathrm{Gdsim}.}$ = Percentage geometrical dissimilarity.

Note: Each variant sequence differs from the reference sequence by multiple terminal deletions of characters. The bolded characters indicate the points of variations.

In contrast, the proposed method also shows variably different percentage similarity scores for these comparisons, which are influenced by the sequence length as expected (Table 9). Additionally, the proposed method is influenced by the terminals in which the deleted bases/amino acids occur. Specifically, when the deleted bases/amino acids occur towards the right terminal end of the sequence, the similarity scores are higher compared to when the deletions occur towards the left terminal end. Therefore, the proposed method takes into account both the sequence length and the position of the deleted bases/amino acids to determine the similarity scores. The proposed method shows 100 % sensitivity to terminal deletions (variations) in the sequences.

These findings underscore the improved sensitivity and discriminatory capabilities of the proposed method in capturing the effects of terminal deletions to the right or the left end within a sequence. This provides a more robust and accurate assessment of sequence similarity.

### Sensitivity to the composition of variations in biological sequence analysis

Our study highlights a significant disparity in percentage similarity scores between conventional methods and our proposed approach, emphasizing the critical role of considering the composition of variations in sequence analysis. Traditional methods overlook the position-specific and character-specific matches, mismatches, and deletions of bases/amino acids in sequence comparisons, limiting their ability to capture the diverse effects of different types of mismatches (such as transition, transversion, isobaric, and non-isobaric mismatches of bases/amino acids in the sequences). These methods primarily rely on counting the number of identical pairs, regardless of their position and character, thereby neglecting other forms of variation. As a result, this method provides similar percentage similarity scores for any comparison between the reference sequence (S_0_) and the variant sequences (S_48_-S_60_) that have a composition of variable parameters (i.e., matches, mismatches, and deletions) (Table 10). The conventional method shows 0 % sensitivity to the composition of variable parameters in the sequences as a result of multiple character(s) mismatches and deletions.

**Table 10:** Sensitivity to the composition of variations in biological sequence analysis.

Aligned DNA and protein sequences		Pairwise percentage similarity between the reference DNA or protein sequences and the variant sequences.						
Reference sequence	Reference sequence	CM	Proposed methods (DNA)			Proposed methods (protein)		
		$\%\mathrm{Sim}.^{*}$	$KC_{\mathrm{Gsim}.}$	$P_{\mathrm{Gsim}.}$	$P_{\mathrm{Gdsim}.}$	$KC_{\mathrm{Gsim}.}$	$P_{\mathrm{Gsim}.}$	$P_{\mathrm{Gdsim}.}$
S_0_ = CTAGCTAGCTAG	S_0_ = GASPCLDQMFRY	**S_0_**	**S_0_**	**S_0_**	**S_0_**	**S_0_**	**S_0_**	**S_0_**
**Variant sequences**	**Variant sequences**							
S_0_ = CTAGCTAGCTAG	S_0_ = GASPCLDQMFRY	100	100	100	100	100	100	100
S_48_ = ***G***TA ***-*** CT***GC***CT ***-*** G	S_48_ = ***Y***AS *-* CL***WS***MF ***-***Y	58.33	0.967486	92.97	7.03	1.002814	99.39	0.61
S_49_ = C ***-******G***G ***-*** TAG***A***TA***T***	S_49_ = G ***-******W***P ***-*** LDQ***G***FR***W***	58.33	0.948862	88.97	11.03	0.969485	93.41	6.59
S_50_ = ***-*** TAG***G***TA***A***C***C***A ***-***	S_50_ = ***-*** ASP***W***LD***A***M***A***R ***-***	58.33	0.966798	92.83	7.17	0.975614	94.73	5.27
S_51_ = C ***- -*** GCTA**AG**TA***A***	S_51_ = G ***- -*** PCLD***PA***FR***S***	58.33	0.933494	85.67	14.33	0.935883	86.18	13.82
S_52_ = ***TC***AGC***G***AGCT***- -***	S_52_ = ***WA***SPC***Y***DQMF ***- -***	58.33	0.993862	98.67	1.33	1.036480	92.65	7.35
S_53_ = C***C***AGC***GC******- -*** TAG	S_53_ = G***Y***SPC***DL- -*** FRY	58.33	0.965412	92.53	7.47	0.991971	98.26	1.74
S_54_ = C***C***AGC***CG***– TAG	S_54_ = G***Y***SPC***DL - -*** FRY	58.33	0.964411	92.31	7.69	0.991920	98.25	1.75
S_55_ = C***A***AGC***CG******- -*** TAG	S_55_ = G***R***SPC***DL - -*** FRY	58.33	0.97129	93.79	6.21	0.989934	97.82	2.18
S_56_ = C***G***AGC***CG******- -*** TAG	S_56_ = GFSPC***DL - -*** FRY	58.33	0.975866	94.78	5.22	0.987380	97.27	2.73
S_57_ = C***- -******A***CTAG***GG***AG	S_57_ = ***- -W***PCLDQ***PP***RY	58.33	0.934985	85.99	14.01	0.970932	93.72	6.28
S_58_ = C***- - A***CTAGC***GAT***	S_58_ = ***- -F*** PCLDQM***YRF***	58.33	0.929291	84.77	15.23	0.969644	93.44	6.56
S_59_ = C ***- - T***CTAGC***ATG***	S_59_ = ***- -R*** PCLDQM***RFY***	58.33	0.926252	84.12	15.88	0.971310	93.80	6.20
S_60_ = C***- -C***CTAGC***AGT***	S_60_ = ***- -Y*** PCLDQM***RYF***	58.33	0.923341	83.50	16.50	0.973404	94.25	5.75
Sensitivity (entropy) for S_48_**-**S_60_		0	3.70	3.70	3.70	3.70	3.70	3.70
% relative sensitivity (entropy) for S_48_**-**S_60_		0	100	100	100	100	100	100

Keys: CM = Conventional method; $\%\;\mathrm{Sim}.^{*}$ = Percentage similarity in DNA and Protein sequences; $KC_{\mathrm{Gsim}.}$ = Kabirian-coefficient of geometrical similarity; $P_{\mathrm{Gsim}.}$ = Percentage geometrical similarity; $P_{\mathrm{Gdsim}.}$ = Percentage geometrical dissimilarity.

Note: Each variant sequence differs from the reference sequence by three mismatches and two deletions. The bolded and italicized character(s) indicate the points of variations.

In contrast, our proposed method takes into account the position-specific and character-specific matches, mismatches, and deletions, resulting in variable percentage similarity scores. Notably, our method exhibits 100 % sensitivity to the composition of variations in the sequences, effectively capturing multiple character(s) mismatches and deletions (Table 10). By considering all observed matches, mismatches, and deletions, our approach enables a comprehensive analysis of sequence variations.

These findings underscore the importance of incorporating position-specific and character-specific information in sequence analysis and demonstrate the enhanced sensitivity of our proposed method to the composition of variations.

## Discussion

### Kabirian-based optinalysis

The introduction of Kabirian-based optinalysis represents a significant paradigm shift in the field of mathematical structure comparison. By leveraging the concepts of automorphism and isomorphism, this method provides a novel approach for estimating symmetry/asymmetry, similarity/dissimilarity, and identity/unidentity measures between isoreflective or autoreflective pairs of mathematical structures.

One of the key strengths of Kabirian-based optinalysis lies in its ability to preserve an equidistant relationship, or isometry, between corresponding pairs of mathematical structures through the use of uniform intervals in the optiscale. This property ensures that the method is robust and consistent in capturing the structural similarities and differences between mathematical entities.

Furthermore, the demonstrated bijective and inverse functional relationship between any pair of points within isoreflective or autoreflective mathematical structures adds to the reliability and validity of Kabirian-based optinalysis. The independence of bijectivity from other pair points further enhances its applicability in accurately comparing isomorphic or automorphic structures.

The invariance properties of Kabirian-based optinalysis under various mathematical operations and transformations, such as scaling, rotation, and location shift, provide additional evidence of its robustness. This means that the outcomes of optinalysis remain consistent regardless of these transformations, further supporting its suitability for symmetry estimation, similarity assessment, and identity measurement.

While Kabirian-based optinalytic measures offer valuable insights and advantages, it is important to acknowledge their limitations. The ordering of the variable(s) in the list must be chosen or established, and the lengths of the variables need to be the same for meaningful comparisons. Additionally, the selection of pairing styles or alternate reflection techniques may influence the results. These considerations highlight the need for careful planning and adherence to specific requirements when applying Kabirian-based optinalysis.

The significance of Kabirian-based optinalysis in research lies in its ability to provide a numerical and mathematical approach to prove functional bijectivity in isomorphic or automorphic structures. By enabling a more precise and reliable comparison of mathematical structures, this method opens up new possibilities for investigating complex relationships, uncovering underlying symmetries, and exploring the depths of mathematical concepts.

The pursuit of a unified theoretical foundation for symmetry/asymmetry, similarity/dissimilarity, and identity/unidentity estimations is a complex and multi-disciplinary endeavor. The exploration of isomorphism, automorphism, and their integration into estimation techniques is another novelty of Kabirian-based optinalysis, which is a crucial step for advancing the coherence and validity of these analyses.

In conclusion, Kabirian-based optinalysis offers a fresh perspective on mathematical structure comparison, driven by its innovative use of automorphism and isomorphism concepts. Its outcomes, including equidistant relationship preservation, bijective functionality, invariance properties, and clear limitations, significantly impact research in mathematics and related fields. This method paves the way for a deeper understanding of complex structural relationships and fosters advancements in the coherence and validity of symmetry, similarity, and identity estimations.

### Sensitivity to position-specific matches and mismatches (substitution)

The incorporation of position-specific nucleotide base mismatches in sequence analysis provides invaluable information for understanding evolutionary processes, identifying genetic variations, and inferring relationships among species [30,44]. However, the conventional metrics used for calculating percentage similarity have disregarded the position-specific aspect of base/amino acid mismatches, leading to limited insights into sequence evolution. By addressing this limitation, the proposed method has the keen sensitivity to detect variations due to the position-specific mismatches in nucleotide base sequences and enhances the accuracy and depth of analysis in various fields, including evolutionary biology, genetics, and phylogenetics [45,46].

The findings highlight the significance of considering the location of base/amino acid mismatches within a sequence. Following the proposed method, the observed increase in percentage similarity scores as the mismatch position moves towards the right terminal end underscores the influence of mismatch location on sequence divergence. This heightened sensitivity to mismatch position(s) contributes to a more comprehensive understanding of sequence evolution and functional implications of base/amino acid substitutions, shedding light on the underlying mechanisms driving genetic diversity and species divergence [47]. Furthermore, this approach has practical implications for the identification of conserved domains, detection of functional motifs, and prediction of protein structures, bolstering research efforts in fields such as drug discovery and protein engineering [48,49].

In conclusion, the incorporation of position-specific base mismatches in sequence analysis represents a novel and powerful approach. It not only addresses the limitations of conventional methods but also provides a deeper and more accurate understanding of sequence evolution, genetic diversity, and functional implications. Its significance spans evolutionary biology, genetics, phylogenetics, and practical applications in fields like drug discovery and protein engineering. This approach is poised to catalyze advancements in our knowledge of the natural world and its applications in various scientific endeavors.

### Sensitivity to character-specific matches and mismatches (substitution)

The conventional methods employed in this study yielded similar percentage similarity scores for comparisons between the reference sequence and the variant sequences with character-specific mismatches resulting from transitional, transversional, isobaric, and non-isobaric alterations [33], [50], [51]. However, the proposed alternative method exhibited variable percentage similarity scores for these comparisons, providing a more nuanced assessment of sequence similarity and evolutionary relationships.

The obtained results revealed that the similarity scores were higher when the base/amino acid mismatch involved a transitional or isobaric alteration [52]. Moreover, these higher similarity scores were observed specifically when the transitional or isobaric mismatch occurred very close to the right terminal end of the sequence. In contrast, when the base/amino acid mismatch involved a transversional or non-isobaric alteration, and was located farther from the right terminal end, the similarity scores were lower. This indicates that the proposed method is particularly sensitive to transitional mismatches, especially when they are positioned towards the right terminal end of the sequence.

These findings highlight the improved discriminatory power of the proposed method in capturing the effects of transitional, transversional, isobaric, and non-isobaric alterations on sequence similarity. By considering both the character-specificity of base/amino acid mismatches and their positional information within the sequence, the proposed method offers a more comprehensive and precise estimation of sequence similarity.

The observed sensitivity of the proposed method to transitional mismatches aligns with previous studies that have demonstrated the higher occurrence frequency of transition substitutions compared to transversion substitutions [44]. The higher similarity scores associated with transitional mismatches could be attributed to their more frequent and potentially less disruptive nature in terms of functional and structural consequences.

Furthermore, the positional dependence of the similarity scores indicates that the right terminal end of the sequence plays a crucial role in determining sequence similarity. Mismatch located closer to this terminal end has a more pronounced impact on similarity scores, suggesting potential functional and evolutionary significance in this region [53].

The results obtained from the proposed method emphasize its potential utility in accurately assessing sequence similarity, deciphering evolutionary relationships, and uncovering functional implications. By incorporating the properties of character-specific matches and mismatches and their positional effects, this alternative method enhances our understanding of sequence evolution, genetic variations, and functional constraints.

In summary, the proposed alternative method for percentage similarity estimation, which incorporates character-specific transitional and transversional mismatches and considers their positional effects, provides a more refined and comprehensive framework for sequence analysis. The variable similarity scores observed in this study highlight the method's sensitivity to transitional mismatches, particularly when located towards the right terminal end of the sequence. This approach holds great promise for advancing our understanding of sequence evolution, genetic variations, and functional implications in various scientific disciplines.

### Sensitivity to position-specific deletion

The conventional methods used for calculating percentage similarity between biological sequences often overlook the position-specificity of base/amino acid deletions within a sequence [33], [50], [51]. These methods do not take into account the location of the deleted base/amino acid, resulting in consistent similarity scores across all comparisons between the reference sequence and variant sequences with position-specific deletions [54]. While these methods provide a convenient way to assess overall sequence similarity, they cannot capture the positional effects of deletions.

In contrast, the proposed method of pairwise percentage similarity estimation accounts for the position of the deleted base/amino acid within the sequence, leading to variably different similarity scores for these comparisons. The obtained results demonstrate a significant trend: as the position of the deleted base/amino acid moves towards the right terminal end of the sequence, the similarity scores increase. This finding highlights the influence of the deletion's location on the similarity assessment, with deletions closer to the right terminal end contributing to higher similarity scores [55].

The observed increase in similarity scores with deletions towards the right terminal end indicates that the proposed method is sensitive to the positional effects of deletions. This sensitivity may reflect the functional and structural implications associated with deletions in different regions of the sequence. Deletions occurring closer to the left terminal end may severely disrupt functional domains or regulatory elements, leading to a more significant impact on sequence comparison [53].

These results underscore the significance of considering the position of deletions in the assessment of sequence similarity. By incorporating position-specific information, the proposed method provides a more comprehensive and accurate estimation of sequence similarity, allowing for a more refined understanding of the evolutionary relationships and functional implications of biological sequences.

In conclusion, the conventional methods used for percentage similarity calculation often overlook the position-specificity of base/amino acid deletions within sequences. In contrast, the proposed method takes into account the position of the deleted base/amino acid and yields variably different similarity scores. The observed increase in similarity scores with deletions towards the right terminal end emphasizes the importance of considering the position of deletions in assessing sequence similarity. By incorporating position-specific information, the proposed method enhances our understanding of sequence evolution, genetic variations, and functional implications in diverse biological fields.

### Sensitivity to terminal deletions

The conventional methods used for calculating percentage similarity between biological sequences often overlook the influence of deletions on similarity scores, regardless of whether they occur at the right or left terminal ends of the sequences [33], [50], [51]. These methods treat all deletions equally, failing to capture the positional effects and potential functional implications associated with deletions at specific locations within the sequence.

In contrast, the proposed method of pairwise percentage similarity estimation considers both the sequence length and the terminals in which the deleted bases/amino acids occur [54]. The results obtained from the comparison demonstrate that the similarity scores obtained from the conventional methods are variable and influenced by the sequence length. As the length of the sequence reduces due to deletions, the similarity scores decrease. However, the terminals in which the deleted bases/amino acids occur do not significantly impact the similarity scores obtained from this conventional method.

In contrast, the proposed method yields variably different percentage similarity scores for these comparisons, taking into account both the sequence length and the position of the deleted bases/amino acids. The results indicate that when the deleted bases/amino acids occur towards the right terminal end of the sequence, the similarity scores are higher compared to when they occur towards the left terminal end. This finding suggests that the proposed method considers the combined effects of sequence length and the positional context of deletions in determining the similarity scores.

The observed differences in similarity scores between the two terminals of the sequence highlight the significance of considering the positional effects of deletions in assessing sequence similarity. Deletions occurring towards the left terminal end may have a more pronounced impact on sequence structure, functional domains, or regulatory elements, potentially leading to lower similarity scores [53]. In contrast, deletions towards the right terminal end may have a relatively lesser impact on the overall structure and function of the sequence, resulting in higher similarity scores.

The sensitivity of the proposed method to the position of deletions enhances the accuracy and depth of analysis in sequence comparison. By incorporating position-specific information, the proposed method provides valuable insights into the evolutionary processes, genetic variations, and functional implications associated with deletions in biological sequences.

In conclusion, while conventional methods overlook the influence of deletions on sequence similarity, the proposed method considers both the sequence length and the positional effects of deletions. The observed differences in similarity scores between the two terminals emphasize the importance of incorporating positional information in assessing sequence similarity. By considering these factors, the proposed method enhances our understanding of evolutionary relationships, genetic variations, and functional implications in biological sequence analysis.

### Sensitivity to the composition of variations in biological sequence analysis

Our study highlights a significant disparity in percentage similarity scores between conventional methods and our proposed approach, emphasizing the critical role of considering the composition of variations in sequence analysis. Traditional methods of computing the percentage similarity between aligned sequences integrated into some bioinformatics tools, such as the widely used Needle, Stretcher, Water, Matcher, MAFFT, MUSCLE, Clustal Omega, and T-Coffee algorithms [28], [29], [30], [31], [32], [33], [34], [35], [36], [37], overlook the position-specific and character-specific matches, mismatches, and deletions of bases/amino acids in sequence comparisons, limiting their ability to capture the diverse effects of different types of mismatches [56], [57], [58].

These methods primarily rely on counting the number of identical pairs, regardless of their position and character, thereby neglecting other forms of variation. As a result, this method provides similar percentage similarity scores for any comparison between the reference sequence (S_0_) and the variant sequences (S_48_-S_60_) that have a composition of variable parameters (i.e., matches, mismatches, and deletions) (Table 8). The conventional method shows low sensitivity to the composition of variable parameters in the sequences as a result of multiple character(s) mismatches and deletions [59].

In contrast, our proposed method takes into account the position-specific and character-specific matches, mismatches, and deletions, resulting in variable percentage similarity scores. Notably, our method exhibits 100 % sensitivity to the composition of variations in the sequences, effectively capturing multiple character(s) mismatches and deletions (Table 8). By considering all observed matches, mismatches, and deletions, our approach enables a comprehensive analysis of sequence variations.

The robustness of the proposed method is underscored by its superior performance compared to conventional methods. The conventional methods, despite their widespread use, exhibit 0 % sensitivity to the composition of variable parameters due to their inability to account for position-specific and character-specific information [60].

In the context of biological sequence analysis, our proposed method holds significant implications. It enables a more comprehensive understanding of sequence variations and their functional implications, allowing researchers to uncover important insights into the structure, function, and evolution of DNA, RNA, and protein sequences [61]. By capturing the differential effects of transition, transversion, isobaric, and non-isobaric mismatches, our method provides a more refined analysis of evolutionary relationships and genetic variations.

Overall, our findings emphasize the robustness and significance of incorporating position-specific and character-specific information in sequence analysis. By offering enhanced sensitivity to the composition of variations, our proposed method provides a valuable tool for researchers in diverse areas, including comparative genomics, metagenomics, molecular epidemiology, and other fields requiring accurate pairwise sequence analysis [62].

### Methodological novelties/innovations

The findings presented in the results have several significant implications for sequence similarity analysis:

1. Improved sensitivity to positional variations: The proposed method demonstrates increased sensitivity to position-specific variations, such as substitutions and indels. By considering the position of matches, mismatches, or deleted bases/amino acids, the method provides more nuanced and informative similarity scores. This enhanced sensitivity allows for a more accurate assessment of the degree of similarity between sequences, particularly when the variations occur towards the right terminal end.
2. Differentiation between transitional, transversional, isobaric, and non-isobaric mismatches: The proposed method highlights the importance of distinguishing between transitional, transversional, isobaric, and non-isobaric mismatches. Transitional mismatches (changes within the same category of nucleotides) or isobaric mismatches (changes within amino acids with closely similar molecular masses) towards the right terminal result in higher similarity scores, indicating a closer relationship between the sequences. In contrast, transversional mismatches (changes between different categories of nucleotides) or non-isobaric mismatches (changes within amino acids with widely similar molecular masses) located farther from the right terminal end lead to lower similarity scores. This differentiation provides valuable insights into the types of variations that have occurred and their impact on sequence similarity.
3. Consideration of indel position and sequence length: The proposed method takes into account the position of deleted bases/amino acids and sequence length. It shows that the similarity scores are influenced by the position of indels, with indels towards the right terminal end resulting in higher similarity scores. Additionally, the method recognizes the influence of sequence length reduction due to indels, resulting in decreasing similarity scores. This consideration of indel position and sequence length provides a more comprehensive evaluation of sequence similarity, accounting for both the local and global effects of indels.

## Conclusion

This study presents a groundbreaking framework, “Kabirian-based optinalysis,” which fundamentally transforms the estimation of symmetry/asymmetry, similarity/dissimilarity, and identity/unidentity in both mathematical structures and biological sequences. By drawing inspiration from the foundational concepts of isomorphism and automorphism, the primary objective was to provide a theoretically grounded framework, addressing the shortcomings of existing methods.

Kabirian-based optinalysis introduces a novel perspective that unifies these estimations under a single theoretical umbrella. It leverages the concept of the optiscale, autoreflective pairing, isoreflective pairing, and others demonstrating robustness and invariance under various mathematical transformations, thus offering a fresh approach to comparing mathematical structures. This framework challenges traditional limitations by establishing functional bijectivity for isomorphic or automorphic structures, paving the way for more accurate and interpretable estimations.

The secondary objective, “geometrical pairwise analysis,” addresses the inadequacies of conventional percentage similarity metrics in biological sequence comparison. It exhibits remarkable sensitivity to position-specific and character-specific variations, enabling a nuanced assessment of sequence similarity.

The novelty of our findings lies in the transformative potential of Kabirian-based optinalysis. It transcends disciplinary boundaries, revolutionizing the analysis of mathematical structures and biological sequences. This holistic approach offers a robust and comprehensive solution, promising to catalyze advancements not only in mathematics and biology but also in computer science, data analysis, pattern recognition, and evolutionary biology.

Finally, Kabirian-based optinalysis opens new horizons for researchers, providing a unified and theoretically grounded framework that has the potential to reshape the way we perceive and analyze complex structures and sequences.

## Future research application

a. Applications and studies related to optinalysis can be designed in five (5) aspects/approaches:
  i. Scaling or re-scaling and formulation. This involves designing a set for the optiscale and expressing equation(s) that can be mathematically proven as a bijection of its isoreflective or autoreflective points. This article has presented Kabirian-based optinalysis as one such approach.
  ii. Parameterization of the domain and codomain. This includes defining what conceptually or empirically represents the domain and the codomain of the isoreflective or autoreflective pair of the variable(s).
  iii. Ordering of the domain and codomain. This includes establishing a theoretical or empirical order for the isoreflective or autoreflective pair of the parameterized variable(s).
  iv. Optimization of the optinalytic operations. This includes optimization in the rotation, reflection, or pairing style of the isoreflective or autoreflective pair of the parameterized and ordered variable(s).
  v. Composition of two or more of the approaches stated in (a)–(c).
b. Further validation and benchmarking studies of the proposed method against established methods are important to evaluate the performance and reliability across different datasets and analysis scenarios.

## Ethics statements

The author declares to comply with the Journal's ethical guidelines.

## CRediT author statement

No other authors state their contribution.

## Declaration of generative AI and AI-assisted technologies in the writing process

During the preparation of this work, the author used *ChatGPT* to improve language, and readability, and clarify some of the mathematical expressions used in the article. After using this tool/service, the author reviewed and edited the content as needed and took full responsibility for the content of the publication.

## Funding

This research did not receive any specific grant from funding agencies in the public, commercial, or not-for-profit sectors.

## Declaration of Competing Interest

The authors declare that they have no known competing financial interests or personal relationships that could have appeared to influence the work reported in this paper.

## Data Availability

Python codes were deposited and published in the online repository, the Mendely Data. The links to the codes implementation of the proposed method and its proposed application in biological sequences were provided in the attachment files. Python Codes for Kabirian-based Automorphic and Isomorphic Optinalysis (Original data) (Mendeley Data) Python Code for Geometrical Pairwise Analysis of Biological Sequences Following Kabirian-based Isomorphic Optinalysis (Original data) (Mendeley Data)

## Appendix Supplementary materials

Image, application 1
