# Supplementary material for: Kabirian-based optinalysis: A conceptually grounded framework for symmetry/asymmetry, similarity/dissimilarity and identity/unidentity estimations in mathematical structures and biological sequences
Source: MethodsX. 2023 Oct 1;11:102400. doi: 10.1016/j.mex.2023.102400 (PMC10622715; doi:10.1016/j.mex.2023.102400)
Supplement: Supplementary file 1 [file mmc1.docx]

Supplementary material

**Appendix A: Functional bijectivity of Kabirian-based automorphic optinalysis**

Theorem: Let $A=(x_{1},x_{2}, x_{3},\ldots,x_{n})$ and its mirror image $A'=({x'}_{1},{x'}_{2}, {x'}_{3},\ldots,{x'}_{n})$ be a mathematical structure. If under Kabirian-based automorphic optinalysis ($K_{c}(A,A')$), and every element of $A$ and $A'$ is expressed as a function, then the resulting functions generate a bijective relationship between corresponding elements of $A$ and $A'$.

*Prove the theorem:*

Let the optinalytic construction of the autoreflective pair of an identical or symmetrical mathematical structure$A$ and its mirror image $A'$ with an assigned optiscale ($R=1, 2, 3, 4, 5, 6, 7$) be:

$$f:\left[ \begin{matrix} A=(x_{1},x_{2}, x_{3}) & \begin{matrix} \delta\\ ⇻ \end{matrix} & A'=({x'}_{3},{x'}_{2}, {x'}_{1}) \\ ↡ & ↡ & ↡ \\ R= (1, 2, 3, & 4, & 5, 6, 7) \end{matrix} \right]$$

Such that $\delta\notin A,A'$; $A,A',\delta\mathbb{\in R}$;$R\subseteq\left\{ r\in\mathbb{R}^{+*} | r=n*k, n\mathbb{\in N,}k>0 \right\}$ or alternatively $R\subseteq\left\{ r\in\mathbb{R}^{-*} | r=-n*k, n\mathbb{\in N,}k>0 \right\}$; and $A \& A'$ are autoreflective pairs about a central point $\delta$.

By Kabirian-based optinalysis (i.e., as in **Eq.A**), each element functions as **Eq.A1 - Eq.A7**:

| $K_{c}=\frac{4(x_{1}+x_{2}+x_{3}+ \delta+{x'}_{3}+{x'}_{2}+{x'}_{1})}{x_{1}+{2x}_{2}+{3x}_{3}+4\delta+{5x'}_{3}+6{x'}_{2}+7{x'}_{1}}$ | (**Eq.A**) |
| --- | --- |
| $x_{1}=\frac{K_{c}\left( 2x_{2}+3x_{3}+ 4\delta+5{x'}_{3}+6{x'}_{2}+7{x'}_{1} \right)-4(x_{2}+x_{3}+ \delta+{x'}_{3}+{x'}_{2}+{x'}_{1})}{4-K_{c}}$ | (**Eq.A1**) |
| $x_{2}=\frac{K_{c}(x_{1}+3x_{3}+ 4\delta+5{x'}_{3}+6{x'}_{2}+7{x'}_{1})-4(x_{1}+x_{3}+ \delta+{x'}_{3}+{x'}_{2}+{x'}_{1})}{4-{2K}_{c}}$ | (**Eq.A2**) |
| $x_{3}=\frac{K_{c}(x_{1}+2x_{2}+ 4\delta+5{x'}_{3}+6{x'}_{2}+7{x'}_{1})-4(x_{1}+x_{2}+ \delta+{x'}_{3}+{x'}_{2}+{x'}_{1})}{4-{3K}_{c}}$ | (**Eq.A3**) |
| $\delta=\frac{K_{c}\left( x_{1}+2x_{2}+3x_{3}+5{x'}_{3}+6{x'}_{2}+7{x'}_{1} \right)-4(x_{1}+x_{2}+x_{3}+{x'}_{3}+{x'}_{2}+{x'}_{1})}{4-{4K}_{c}}$ | (**Eq.A4**) |
| ${x'}_{3}=\frac{K_{c}\left( x_{1}+2x_{2}+3x_{3}+ 4\delta+6{x'}_{2}+7{x'}_{1} \right)-4(x_{1}+x_{2}+x_{3}+ \delta+{x'}_{2}+{x'}_{1})}{4-{5K}_{c}}$ | (**Eq.A5**) |
| ${x'}_{2}=\frac{K_{c}\left( x_{1}+2x_{2}+3x_{3}+ 4\delta+5{x'}_{3}+7{x'}_{1} \right)-4(x_{1}+x_{2}+x_{3}+ \delta+{x'}_{3}+{x'}_{1})}{4-{6K}_{c}}$ | (**Eq.A6**) |
| ${x'}_{1}=\frac{K_{c}\left( x_{1}+2x_{2}+3x_{3}+ 4\delta+5{x'}_{3}+6{x'}_{2} \right)-4(x_{1}+x_{2}+x_{3}+ \delta+{x'}_{3}+{x'}_{2})}{4-{7K}_{c}}$ | (**Eq.A7**) |

Recall the definition of bijective mapping (*one-to-one and onto*), such that if $x=y$, then $f(g\left( x \right))=g(f\left( y \right))$. We now have three (3) cases evaluated as follows:

Case A1:

Firstly, we verify if the pair of autoreflective points (i.e., $x_{1}$ and ${x'}_{1}$) are functionally mapped *one-to-one*.

$$x_{1}={x'}_{1}\Rightarrow\frac{K_{c}\left( {2x}_{2}+{3x}_{3}+4\delta+{5x'}_{3}+6{x'}_{2}+7{x'}_{1} \right)-4(x_{2}+x_{3}+ \delta+{x'}_{3}+{x'}_{2}+{x'}_{1})}{4-K_{c}}=\frac{K_{c}(x_{1}+{2x}_{2}+{3x}_{3}+4\delta+{5x'}_{3}+6{x'}_{2})-4(x_{1}+x_{2}+x_{3}+\delta+{x'}_{3}+{x'}_{2})}{4-7K_{c}}$$

$$x_{1}={x'}_{1}\Rightarrow\frac{K_{c}\left( {2x}_{2}+{3x}_{3}+4\delta+{5x'}_{3}+6{x'}_{2} \right)-4(x_{2}+x_{3}+ \delta+{x'}_{3}+{x'}_{2})}{4-K_{c}}+\frac{K_{c}(7{x'}_{1})-4{x'}_{1}}{4-K_{c}}=\frac{K_{c}({2x}_{2}+{3x}_{3}+4\delta+{5x'}_{3}+6{x'}_{2})-4(x_{2}+x_{3}+\delta+{x'}_{3}+{x'}_{2})}{4-7K_{c}}+\frac{K_{c}(x_{1})-4x_{1}}{4-7K_{c}}$$

When two completely symmetrical or identical pairs of mathematical structures are compared optinalytically, then $K_{c}=1$.

Therefore, we now have

| $x_{1}={x'}_{1}\Rightarrow\frac{\left( {2x}_{2}+{3x}_{3}+4\delta+{5x'}_{3}+6{x'}_{2} \right)-4(x_{2}+x_{3}+ \delta+{x'}_{3}+{x'}_{2})}{3}+\frac{7{x'}_{1}-4{x'}_{1}}{3}=\frac{({2x}_{2}+{3x}_{3}+4\delta+{5x'}_{3}+6{x'}_{2})-4(x_{2}+x_{3}+\delta+{x'}_{3}+{x'}_{2})}{-3}+\frac{x_{1}-4x_{1}}{-3}$ | (**Eq.A8**) |
| --- | --- |

Let the common factor for both sides of **Eq.A8** be $p_{1}=\left( {2x}_{2}+{3x}_{3}+4\delta+{5y}_{3}+6y_{2} \right)-4(x_{2}+x_{3}+ \delta+y_{3}+y_{2})$

$$x_{1}={x'}_{1}\Rightarrow\frac{p_{1}+3{x'}_{1}}{3}=\frac{-p_{1}-3x_{1}}{-3}$$

Secondly, we verify if the pair of autoreflective points (i.e., $x_{1}$ and ${x'}_{1}$) are functionally mapped *onto* each other.

By composing $g({x'}_{1}$) onto $f(x_{1})$

$$f\left( g\left( x_{1} \right) \right)= \frac{p_{1}+3\left( \frac{-p_{1}-3x_{1}}{-3} \right)}{3}=\frac{-3p_{1}+3p_{1}-9x_{1}}{-9}=x_{1}\Rightarrow{f\left( x_{1} \right)=g}^{-1}({x'}_{1})$$

Again by composing $f(x_{1}$) onto $g({x'}_{1})$

$$g\left( f\left( {x'}_{1} \right) \right)\Rightarrow\frac{-p_{1}-3\left( \frac{p_{1}+3{x'}_{1}}{3} \right)}{-3}=\frac{3p_{1}-3p_{1}-9{x'}_{1}}{-9}={x'}_{1}\Rightarrow{g\left( {x'}_{1} \right)=f}^{-1}(x_{1})$$

Finally, since $x_{1}$ and ${x'}_{1}$ are *one-to-one and onto*, we conclude that $x_{1}$ and ${x'}_{1}$ are functionally bijective and inverse.

Case A2:

Firstly, we verify if the pair of autoreflective points (i.e., $x_{2}$ and ${x'}_{2}$) are functionally mapped *one-to-one*.

$$x_{2}={x'}_{2}\Rightarrow\frac{K_{c}\left( x_{1}+{3x}_{3}+4\delta+{5x'}_{3}+6{x'}_{2}+7{x'}_{1} \right)-4(x_{1}+x_{3}+ \delta+{x'}_{3}+{x'}_{2}+{x'}_{1})}{4-2K_{c}}=\frac{K_{c}(x_{1}+{2x}_{2}+{3x}_{3}+4\delta+{5x'}_{3}+7{yx'}_{1})-4(x_{1}+x_{2}+x_{3}+\delta+{x'}_{3}+{x'}_{1})}{4-6K_{c}}$$

$$x_{2}={x'}_{2}\Rightarrow\frac{K_{c}\left( x_{1}+{3x}_{3}+4\delta+{5x'}_{3}+7{x'}_{1} \right)-4(x_{1}+x_{3}+ \delta+{x'}_{3}+{x'}_{1})}{4-2K_{c}}+\frac{K_{c}(6{x'}_{2})-4{x'}_{2}}{4-{2K}_{c}}=\frac{K_{c}(x_{1}+{3x}_{3}+4\delta+{5x'}_{3}+7{x'}_{1})-4(x_{1}+x_{3}+\delta+{x'}_{3}+{x'}_{1})}{4-6K_{c}}+\frac{K_{c}(2x_{2})-4x_{2}}{4-{6K}_{c}}$$

When two completely symmetrical or identical pairs of mathematical structures are compared optinalytically, then $K_{c}=1$.

Therefore, we now have

| $x_{2}={x'}_{2}\Rightarrow\frac{\left( x_{1}+{3x}_{3}+4\delta+{5x'}_{3}+7{x'}_{1} \right)-4(x_{1}+x_{3}+ \delta+{x'}_{3}+{x'}_{1})}{2}+\frac{6{x'}_{2}-4{x'}_{2}}{2}=\frac{(x_{1}+{3x}_{3}+4\delta+{5x'}_{3}+7{x'}_{1})-4(x_{1}+x_{3}+\delta+{x'}_{3}+{x'}_{1})}{-2}+\frac{2x_{2}-4x_{2}}{-2}$ | (**Eq.A9**) |
| --- | --- |

Let the common factor for both sides of **Eq.A9** be $p_{2}=\left( x_{1}+{3x}_{3}+4\delta+{5x'}_{3}+7{x'}_{1} \right)-4(x_{1}+x_{3}+ \delta+{x'}_{3}+{x'}_{1})$

$$x_{2}={x'}_{2}\Rightarrow\frac{p_{2}+2{x'}_{2}}{2}=\frac{{-p}_{2}-2x_{2}}{-2}$$

Secondly, we verify if the pair of autoreflective points (i.e., $x_{2}$ and ${x'}_{2}$) are functionally mapped *onto* each other.

By composing $g({x'}_{2}$) onto $f(x_{2})$

$$f\left( g\left( x_{2} \right) \right)= \frac{p_{2}+2\left( \frac{{-p}_{2}-2x_{2}}{-2} \right)}{2}=\frac{-2p_{2}+2p_{2}-4x_{2}}{-4}=x_{2}\Rightarrow{f\left( x_{2} \right)=g}^{-1}({x'}_{2})$$

Again by composing $f(x_{2}$) onto $g({x'}_{2})$

$$g\left( f\left( {x'}_{2} \right) \right)\Rightarrow\frac{-p_{2}-2\left( \frac{p_{2}+2{x'}_{2}}{2} \right)}{-2}=\frac{2p_{2}-2p_{2}-4{x'}_{1}}{-4}=y_{2}\Rightarrow{g\left( {x'}_{2} \right)=f}^{-1}(x_{2})$$

Finally, since $x_{2}$ and ${x'}_{2}$ are *one-to-one and onto*, we conclude that $x_{2}$ and ${x'}_{2}$ are functionally bijective and inverse.

Case A3:

Firstly, we verify if the pair of autoreflective points (i.e., $x_{3}$ and ${x'}_{3}$) are functionally mapped *one-to-one*.

$$x_{3}={x'}_{3}\Rightarrow\frac{K_{c}\left( {x_{1}+2x}_{2}+4\delta+{5x'}_{3}+6{x'}_{2}+7{x'}_{1} \right)-4(x_{1}+x_{2}+ \delta+{x'}_{3}+{x'}_{2}+{x'}_{1})}{4-3K_{c}}=\frac{K_{c}(x_{1}+{2x}_{2}+{3x}_{3}+4\delta+6{x'}_{2}+7{x'}_{1})-4(x_{1}+x_{2}+x_{3}+\delta+{x'}_{2}+{x'}_{1})}{4-5K_{c}}$$

$$x_{3}={x'}_{3}\Rightarrow\frac{K_{c}\left( {x_{1}+2x}_{2}+4\delta+6{x'}_{2}+7{x'}_{1} \right)-4(x_{1}+x_{2}+ \delta+{x'}_{2}+{x'}_{1})}{4-3K_{c}}+\frac{K_{c}(5{x'}_{3})-4{x'}_{3}}{4-{3K}_{c}}=\frac{K_{c}(x_{1}+{2x}_{2}+4\delta+6{x'}_{2}+7{x'}_{1})-4(x_{1}+x_{2}+\delta+{x'}_{2}+{x'}_{1})}{4-5K_{c}}+\frac{K_{c}(3x_{3})-4x_{3}}{4-{5K}_{c}}$$

When two completely symmetrical or identical pairs of mathematical structures are compared optinalytically, then $K_{c}=1$.

Therefore, we now have

| $x_{3}={x'}_{3}\Rightarrow\frac{\left( {x_{1}+2x}_{2}+4\delta+6{x'}_{2}+7{x'}_{1} \right)-4(x_{1}+x_{2}+ \delta+{x'}_{2}+{x'}_{1})}{1}+\frac{5{x'}_{3}-4{x'}_{3}}{1}=\frac{(x_{1}+{2x}_{2}+4\delta+6{x'}_{2}+7{x'}_{1})-4(x_{1}+x_{2}+\delta+{x'}_{2}+{x'}_{1})}{-1}+\frac{3x_{3}-4x_{3}}{-1}$ | (**Eq.A10**) |
| --- | --- |

Let the common factor to both sides of the equation **Eq.A10**. be $p_{3}=\left( {x_{1}+2x}_{2}+4\delta+6{x'}_{2}+7{x'}_{1} \right)-4(x_{1}+x_{2}+ \delta+{x'}_{2}+{x'}_{1})$

$$x_{3}={x'}_{3}\Rightarrow\frac{p_{3}+{x'}_{3}}{1}=\frac{-p_{3}-x_{3}}{-1}$$

Secondly, we verify if the pair of autoreflective points (i.e., $x_{3}$ and ${x'}_{3}$) are functionally mapped *onto* each other.

By composing $g({x'}_{3}$) onto $f(x_{3})$

$$f\left( g\left( x_{3} \right) \right)= \frac{p_{3}+\frac{(-p_{3}-x_{3)}}{-1}}{1}=\frac{-p_{3}+p_{3}-x_{3}}{-1}=x_{3}\Rightarrow{f\left( x_{3} \right)=g}^{-1}({x'}_{3})$$

Again by composing $f(x_{3}$) onto $g({x'}_{3})$

$$g\left( f\left( {x'}_{3} \right) \right)\Rightarrow\frac{p_{3}-\frac{p_{3}+{x'}_{3}}{1}}{-1}=\frac{p_{3}-p_{3}-{x'}_{3}}{-1}=y_{3}\Rightarrow{g\left( {x'}_{3} \right)=f}^{-1}(x_{3})$$

Finally, since $\boldsymbol{x}_{\boldsymbol{3}}$ and $\boldsymbol{x'}_{\boldsymbol{3}}$ are *one-to-one and onto*, we conclude that $\boldsymbol{x}_{\boldsymbol{3}}$ and $\boldsymbol{y}_{\boldsymbol{3}}$ are functionally bijective and inverse.

**Appendix B: Functional bijectivity of Kabirian-based isomorphic optinalysis**

Theorem: Let $A=(x_{1},x_{2}, x_{3},\ldots,x_{n})$ and $B=(y_{1},y_{2}, y_{3},\ldots,y_{n}$ be mathematical structures. If under Kabirian-based isomorphic optinalysis ($K_{c}(A,B)$), and every element of $A$and $B$ is expressed as a function, then the resulting functions generate a bijective relationship between corresponding elements of $A$ and $B$.

*Prove theorem:*

Let the optinalytic construction of an isoreflective pair of identical or similar mathematical structures $A$ and $B$ with an assigned optiscale ($R=1, 2, 3, 4, 5, 6, 7$) be:

$$f:\left[ \begin{matrix} A=(x_{1},x_{2}, x_{3}) & \begin{matrix} \delta\\ ⇻ \end{matrix} & B=(y_{3},y_{2}, y_{1}) \\ ↡ & ↡ & ↡ \\ R= (1, 2, 3, & 4, & 5, 6, 7) \end{matrix} \right]$$

Such that $\delta\notin A,B$; $A,B,\delta\mathbb{\in R}$;$R\subseteq\left\{ r\in\mathbb{R}^{+*} | r=n*k, n\mathbb{\in N,}k>0 \right\}$ or alternatively$R\subseteq\left\{ r\in\mathbb{R}^{-*} | r=-n*k, n\mathbb{\in N,}k>0 \right\}$; and $A \& B$ are isoreflective pair on a chosen pairing about a central point $\delta$.

By Kabirian-based optinalysis (i.e., as in **Eq.B** ), each element functions as **Eq.B1 - Eq.B7**:

| $K_{c}=\frac{4(x_{1}+x_{2}+x_{3}+ \delta+y_{3}+y_{2}+y_{1})}{x_{1}+{2x}_{2}+{3x}_{3}+4\delta+{5y}_{3}+6y_{2}+7y_{1}}$ | (**Eq.B**) |
| --- | --- |
| $x_{1}=\frac{K_{c}\left( 2x_{2}+3x_{3}+ 4\delta+5y_{3}+6y_{2}+7y_{1} \right)-4(x_{2}+x_{3}+ \delta+y_{3}+y_{2}+y_{1})}{4-K_{c}}$ | (**Eq.B1**) |
| $x_{2}=\frac{K_{c}(x_{1}+3x_{3}+ 4\delta+5y_{3}+6y_{2}+7y_{1})-4(x_{1}+x_{3}+ \delta+y_{3}+y_{2}+y_{1})}{4-{2K}_{c}}$ | (**Eq.B2**) |
| $x_{3}=\frac{K_{c}(x_{1}+2x_{2}+ 4\delta+5y_{3}+6y_{2}+7y_{1})-4(x_{1}+x_{2}+ \delta+y_{3}+y_{2}+y_{1})}{4-{3K}_{c}}$ | (**Eq.B3**) |
| $\delta=\frac{K_{c}\left( x_{1}+2x_{2}+3x_{3}+5y_{3}+6y_{2}+7y_{1} \right)-4(x_{1}+x_{2}+x_{3}+y_{3}+y_{2}+y_{1})}{4-{4K}_{c}}$ | (**Eq.B4**) |
| $y_{3}=\frac{K_{c}\left( x_{1}+2x_{2}+3x_{3}+ 4\delta+6y_{2}+7y_{1} \right)-4(x_{1}+x_{2}+x_{3}+ \delta+y_{2}+y_{1})}{4-{5K}_{c}}$ | (**Eq.B5**) |
| $y_{2}=\frac{K_{c}\left( x_{1}+2x_{2}+3x_{3}+ 4\delta+5y_{3}+7y_{1} \right)-4(x_{1}+x_{2}+x_{3}+ \delta+y_{3}+y_{1})}{4-{6K}_{c}}$ | (**Eq.B6**) |
| $y_{1}=\frac{K_{c}\left( x_{1}+2x_{2}+3x_{3}+ 4\delta+5y_{3}+6y_{2} \right)-4(x_{1}+x_{2}+x_{3}+ \delta+y_{3}+y_{2})}{4-{7K}_{c}}$ | (**Eq.B7**) |

Recall the definition of bijective mapping (*one-to-one and onto*), such that if $x=y$, then $f(g\left( x \right))=g(f\left( y \right))$. We now have three (3) cases evaluated as follows:

Case B1:

Firstly, we verify if the pair of isoreflective points (i.e., $x_{1}$ and $y_{1}$) are functionally mapped *one-to-one*.

$$x_{1}=y_{1}\Rightarrow\frac{K_{c}\left( {2x}_{2}+{3x}_{3}+4\delta+{5y}_{3}+6y_{2}+7y_{1} \right)-4(x_{2}+x_{3}+ \delta+y_{3}+y_{2}+y_{1})}{4-K_{c}}=\frac{K_{c}(x_{1}+{2x}_{2}+{3x}_{3}+4\delta+{5y}_{3}+6y_{2})-4(x_{1}+x_{2}+x_{3}+\delta+y_{3}+y_{2})}{4-7K_{c}}$$

$$x_{1}=y_{1}\Rightarrow\frac{K_{c}\left( {2x}_{2}+{3x}_{3}+4\delta+{5y}_{3}+6y_{2} \right)-4(x_{2}+x_{3}+ \delta+y_{3}+y_{2})}{4-K_{c}}+\frac{K_{c}(7y_{1})-4y_{1}}{4-K_{c}}=\frac{K_{c}({2x}_{2}+{3x}_{3}+4\delta+{5y}_{3}+6y_{2})-4(x_{2}+x_{3}+\delta+y_{3}+y_{2})}{4-7K_{c}}+\frac{K_{c}(x_{1})-4x_{1}}{4-7K_{c}}$$

When two completely similar or identical pairs of mathematical structures are compared optinalytically, then $K_{c}=1$.

Therefore, we now have

| $x_{1}=y_{1}\Rightarrow\frac{\left( {2x}_{2}+{3x}_{3}+4\delta+{5y}_{3}+6y_{2} \right)-4(x_{2}+x_{3}+ \delta+y_{3}+y_{2})}{3}+\frac{7y_{1}-4y_{1}}{3}=\frac{({2x}_{2}+{3x}_{3}+4\delta+{5y}_{3}+6y_{2})-4(x_{2}+x_{3}+\delta+y_{3}+y_{2})}{-3}+\frac{x_{1}-4x_{1}}{-3}$ | (**Eq.B8**) |
| --- | --- |

Let the common factor for both sides of **Eq.B8** be $p_{1}=\left( {2x}_{2}+{3x}_{3}+4\delta+{5y}_{3}+6y_{2} \right)-4(x_{2}+x_{3}+ \delta+y_{3}+y_{2})$

$$x_{1}=y_{1}\Rightarrow\frac{p_{1}+3y_{1}}{3}=\frac{-p_{1}-3x_{1}}{-3}$$

Secondly, we verify if the pair of isoreflective points (i.e., $x_{1}$ and $y_{1}$) are functionally mapped *onto* each other.

By composing $g(y_{1}$) onto $f(x_{1})$

$$f\left( g\left( x_{1} \right) \right)= \frac{p_{1}+3\left( \frac{-p_{1}-3x_{1}}{-3} \right)}{3}=\frac{-3p_{1}+3p_{1}-9x_{1}}{-9}=x_{1}\Rightarrow{f\left( x_{1} \right)=g}^{-1}(y_{1})$$

Again by composing $f(x_{1}$) onto $g(y_{1})$

$$g\left( f\left( y_{1} \right) \right)\Rightarrow\frac{-p_{1}-3\left( \frac{p_{1}+3y_{1}}{3} \right)}{-3}=\frac{3p_{1}-3p_{1}-9y_{1}}{-9}=y_{1}\Rightarrow{g\left( y_{1} \right)=f}^{-1}(x_{1})$$

Finally, since $x_{1}$ and $y_{1}$ are *one-to-one and onto*, we conclude that $x_{1}$ and $y_{1}$ are functionally bijective and inverse.

Case B2:

Firstly, we verify if the pair of isoreflective points (i.e., $x_{2}$ and $y_{2}$) are functionally mapped *one-to-one*.

$$x_{2}=y_{2}\Rightarrow\frac{K_{c}\left( x_{1}+{3x}_{3}+4\delta+{5y}_{3}+6y_{2}+7y_{1} \right)-4(x_{1}+x_{3}+ \delta+y_{3}+y_{2}+y_{1})}{4-2K_{c}}=\frac{K_{c}(x_{1}+{2x}_{2}+{3x}_{3}+4\delta+{5y}_{3}+7y_{1})-4(x_{1}+x_{2}+x_{3}+\delta+y_{3}+y_{1})}{4-6K_{c}}$$

$$x_{2}=y_{2}\Rightarrow\frac{K_{c}\left( x_{1}+{3x}_{3}+4\delta+{5y}_{3}+7y_{1} \right)-4(x_{1}+x_{3}+ \delta+y_{3}+y_{1})}{4-2K_{c}}+\frac{K_{c}(6y_{2})-4y_{2}}{4-{2K}_{c}}=\frac{K_{c}(x_{1}+{3x}_{3}+4\delta+{5y}_{3}+7y_{1})-4(x_{1}+x_{3}+\delta+y_{3}+y_{1})}{4-6K_{c}}+\frac{K_{c}(2x_{2})-4x_{2}}{4-{6K}_{c}}$$

When two completely similar or identical pairs of mathematical structures are compared optinalytically, then $K_{c}=1$.

Therefore, we now have

| $x_{2}=y_{2}\Rightarrow\frac{\left( x_{1}+{3x}_{3}+4\delta+{5y}_{3}+7y_{1} \right)-4(x_{1}+x_{3}+ \delta+y_{3}+y_{1})}{2}+\frac{6y_{2}-4y_{2}}{2}=\frac{(x_{1}+{3x}_{3}+4\delta+{5y}_{3}+7y_{1})-4(x_{1}+x_{3}+\delta+y_{3}+y_{1})}{-2}+\frac{2x_{2}-4x_{2}}{-2}$ | (**Eq.B9**) |
| --- | --- |

Let the common factor for both sides of **Eq.B9** be $p_{2}=\left( x_{1}+{3x}_{3}+4\delta+{5y}_{3}+7y_{1} \right)-4(x_{1}+x_{3}+ \delta+y_{3}+y_{1})$

$$x_{2}=y_{2}\Rightarrow\frac{p_{2}+2y_{2}}{2}=\frac{{-p}_{2}-2x_{2}}{-2}$$

Secondly, we verify if the pair of isoreflective points (i.e., $x_{2}$ and $y_{2}$) are functionally mapped *onto* each other.

By composing $g(y_{2}$) onto $f(x_{2})$

$$f\left( g\left( x_{2} \right) \right)= \frac{p_{2}+2\left( \frac{{-p}_{2}-2x_{2}}{-2} \right)}{2}=\frac{-2p_{2}+2p_{2}-4x_{2}}{-4}=x_{2}\Rightarrow{f\left( x_{2} \right)=g}^{-1}(y_{2})$$

Again by composing $f(x_{2}$) onto $g(y_{2})$

$$g\left( f\left( y_{2} \right) \right)\Rightarrow\frac{-p_{2}-2\left( \frac{p_{2}+2y_{2}}{2} \right)}{-2}=\frac{2p_{2}-2p_{2}-4y_{1}}{-4}=y_{2}\Rightarrow{g\left( y_{2} \right)=f}^{-1}(x_{2})$$

Finally, since $x_{2}$ and $y_{2}$ are *one-to-one and onto*, we conclude that $x_{2}$ and $y_{2}$ are functionally bijective and inverse.

Case B3:

Firstly, we verify if the pair isoreflective points (i.e., $x_{3}$ and $y_{3}$) are functionally mapped *one-to-one*.

$$x_{3}=y_{3}\Rightarrow\frac{K_{c}\left( {x_{1}+2x}_{2}+4\delta+{5y}_{3}+6y_{2}+7y_{1} \right)-4(x_{1}+x_{2}+ \delta+y_{3}+y_{2}+y_{1})}{4-3K_{c}}=\frac{K_{c}(x_{1}+{2x}_{2}+{3x}_{3}+4\delta+6y_{2}+7y_{1})-4(x_{1}+x_{2}+x_{3}+\delta+y_{2}++y_{1})}{4-5K_{c}}$$

$$x_{3}=y_{3}\Rightarrow\frac{K_{c}\left( {x_{1}+2x}_{2}+4\delta+6y_{2}+7y_{1} \right)-4(x_{1}+x_{2}+ \delta+y_{2}+y_{1})}{4-3K_{c}}+\frac{K_{c}(5y_{3})-4y_{3}}{4-{3K}_{c}}=\frac{K_{c}(x_{1}+{2x}_{2}+4\delta+6y_{2}+7y_{1})-4(x_{1}+x_{2}+\delta+y_{2}++y_{1})}{4-5K_{c}}+\frac{K_{c}(3x_{3})-4x_{3}}{4-{5K}_{c}}$$

When two completely similar or identical pairs of mathematical structures are compared optinalytically, then $K_{c}=1$.

Therefore, we now have

| $x_{3}=y_{3}\Rightarrow\frac{\left( {x_{1}+2x}_{2}+4\delta+6y_{2}+7y_{1} \right)-4(x_{1}+x_{2}+ \delta+y_{2}+y_{1})}{1}+\frac{5y_{3}-4y_{3}}{1}=\frac{(x_{1}+{2x}_{2}+4\delta+6y_{2}+7y_{1})-4(x_{1}+x_{2}+\delta+y_{2}++y_{1})}{-1}+\frac{3x_{3}-4x_{3}}{-1}$ | (**Eq.B10**) |
| --- | --- |

Let the common factor for both sides of **Eq.B10** be $p_{3}=\left( {x_{1}+2x}_{2}+4\delta+6y_{2}+7y_{1} \right)-4(x_{1}+x_{2}+ \delta+y_{2}+y_{1})$

$$x_{3}=y_{3}\Rightarrow\frac{p_{3}+y_{3}}{1}=\frac{-p_{3}-x_{3}}{-1}$$

Secondly, we verify if the pair of isoreflective points (i.e., $x_{3}$ and $y_{3}$) are functionally mapped *onto* each other.

By composing $g(y_{3}$) onto $f(x_{3})$

$$f\left( g\left( x_{3} \right) \right)= \frac{p_{3}+\frac{(-p_{3}-x_{3)}}{-1}}{1}=\frac{-p_{3}+p_{3}-x_{3}}{-1}=x_{3}\Rightarrow{f\left( x_{3} \right)=g}^{-1}(y_{3})$$

Again by composing $f(x_{3}$) onto $g(y_{3})$

$$g\left( f\left( y_{3} \right) \right)\Rightarrow\frac{p_{3}-\frac{p_{3}+y_{3}}{1}}{-1}=\frac{p_{3}-p_{3}-y_{3}}{-1}=y_{3}\Rightarrow{g\left( y_{3} \right)=f}^{-1}(x_{3})$$

Finally, since $x_{3}$ and $y_{3}$ are *one-to-one and onto*, we conclude that $x_{3}$ and $y_{3}$ are functionally bijective and inverse.

**Appendix C: Transformation invariance of completeness**

Theorem: Let $A=(x_{1},x_{2}, x_{3},\ldots,x_{n})$ and its mirror image $A'=\left( {x'}_{1},{x'}_{'2}, {x'}_{3},\ldots,{x'}_{n} \right)$ be a mathematical structure. Let $A=(x_{1},x_{2}, x_{3},\ldots,x_{n})$ and $B=\left( x_{1},x_{2}, x_{3},\ldots,x_{n} \right)$ be two mathematical structures. If under Kabirian-based automorphic optinalysis ($Opt(A,A')$), or Kabirian-based isomorphic optinalysis ($Opt(A,B)$); and $A$ and $A'$ are completely symmetrical/identical, or $A$ and $B$ are completely similar/identical; then the estimates are invariant to transformations such as pericentral rotation (alternate reflection), central rotation (inversion), translation (location shift), scaling, and central modulation.

*Prove of theorem of transformation-invariance: location-invariance*

Let the optinalytic construction of an isoreflective pair of mathematical structures $A$ and $B$ with an assigned optiscale ($R=1, 2, 3, 4, 5, 6, 7$) be:

$$f:\left[ \begin{matrix} A=(x_{1},x_{2},x_{3}) & \begin{matrix} \delta\\ ⇻ \end{matrix} & B=(x_{3}, x_{2},x_{1}) \\ ↡ & ↡ & ↡ \\ R= (1, 2, 3, & 4, & 5, 6, 7) \end{matrix} \right]$$

Let $c$ be a location shift. The optinalytic construction becomes:

$$f:\left[ \begin{matrix} A=\left[ \left( x_{1}+c \right),\left( x_{2}+c \right),(x_{3}+c) \right] & \begin{matrix} \delta\\ ⇻ \end{matrix} & B=\left[ \left( x_{3}+c \right),\left( x_{2}+c \right),(x_{1}+c) \right] \\ ↡ & ↡ & ↡ \\ R= (1, 2, 3, & 4, & 5, 6, 7) \end{matrix} \right]$$

Such that $\delta\notin A, B,c$; $A,B,\delta, c\mathbb{\in R}$;$R\subseteq\left\{ r\in\mathbb{R}^{+*} | r=n*k, n\mathbb{\in N,}k>0 \right\}$ or alternatively $R\subseteq\left\{ r\in\mathbb{R}^{-*} | r=-n*k, n\mathbb{\in N,}k>0 \right\}$; and $A \& B$ are isoreflective pair on a chosen pairing about a central point $\delta$.

Following Kabirian-based isomorphic optinalysis, we check the condition that $A$ is completely similar or identical to $B$. Then, we have

$${KC}_{Sim./Id.}\left( A,B \right)=\frac{4(x_{1}+x_{2}+x_{3}+\delta+x_{3}+ x_{2}+x_{1})}{x_{1}+2x_{2}+3x_{3}+4\delta+5x_{3}+6x_{2}+7x_{1}}$$

$${KC}_{Sim./Id.}\left( A,B \right)=\frac{8x_{1}+8x_{2}+8x_{3}+4\delta}{8x_{1}+8x_{2}+8x_{3}+4\delta}=1$$

Having satisfied the condition above, we transform by location shift as:

$${KC}_{Sim./Id.}\left( A+c,B+c \right)=\frac{4\left[ (x_{1}+c)+(x_{2}+c)+(x_{3}+c)+(\delta+c)+(x_{3}+c)+( x_{2}+c)+(x_{1}+c) \right]}{(x_{1}+c)+(2x_{2}+c)+(3x_{3}+c)+(4\delta+c)+(5x_{3}+c)+(6x_{2}+c)+(7x_{1}+c)}$$

$${KC}_{Sim./Id.}\left( A+c,B+c \right)=\frac{8 \left( x_{1}+c \right)+ 8 \left( x_{2}+c \right)+8 (x_{3}+c)+4\delta}{8 \left( x_{1}+c \right)+ 8 \left( x_{2}+c \right)+8 (x_{3}+c)+4\delta}=\frac{8x_{1}+8x_{2}+8x_{3}+4\delta+24c}{8x_{1}+8x_{2}+8x_{3}+4\delta+24c}=1$$

We now conclude that ${KC}_{Sim./Id.}\left( A,B \right)={KC}_{Sim./Id.}\left( A+c,B+c \right)$.Therefore,

If ${KC}_{Sim./Id.}\left( A,B \right)=1$, then ${KC}_{Sim./Id.}\left( A+c,B+c \right)=1$

Similarly, the relationship is the same following the Kabirian-based optinalysis-to-probability translation models.

If $P_{Sim./Id.}\left( A,B \right)=1$, then $P_{Sim./Id.}\left( A+c,B+c \right)=1$

If $P_{Dsim./Uid.}\left( A,B \right)=0$, then $P_{Dsim./Uid.}\left( A+c,B+c \right)=0$

Let $A=(x_{1},x_{2}, x_{3},\ldots,x_{n})$ and its mirror image $A'=\left( {x'}_{1},{x'}_{'2}, {x'}_{3},\ldots,{x'}_{n} \right)$ be a mathematical structure. Following the Kabirian-based automorphic optinalysis and optinalysis-to-probability translation models, a similar conclusion will be drawn:

If ${KC}_{Sym./Id.}\left( A,A' \right)=1$, then ${KC}_{Sym./Id.}\left( A+c,A^{'}+c \right)=1$

If $P_{Sym./Id.}\left( A,A' \right)=1$, then $P_{Sym./Id.}\left( A+c,A'+c \right)=1$

If $P_{Asym./Uid.}\left( A,A' \right)=0$, then $P_{Asym./Uid.}\left( A+c,A'+c \right)=0$

Therefore, Kabirian-based optinalysis is a location-invariant.

*Prove of a theorem of transformation-invariance: scale-invariance*

Let the optinalytic construction of an isoreflective pair of mathematical structures $A$ and $B$ with an assigned optiscale ($R=1, 2, 3, 4, 5, 6, 7$) be:

$$f:\left[ \begin{matrix} A=(x_{1},x_{2},x_{3}) & \begin{matrix} \delta\\ ⇻ \end{matrix} & B=(x_{3}, x_{2},x_{1}) \\ ↡ & ↡ & ↡ \\ R= (1, 2, 3, & 4, & 5, 6, 7) \end{matrix} \right]$$

Let $c$ be a scaling factor. The optinalytic construction becomes:

$$f:\left[ \begin{matrix} A=(cx_{1}, cx_{2},cx_{3}) & \begin{matrix} \delta\\ ⇻ \end{matrix} & B=(cx_{3},cx_{2},cx_{1}) \\ ↡ & ↡ & ↡ \\ R= (1, 2, 3, & 4, & 5, 6, 7) \end{matrix} \right]$$

Such that $\delta\notin A, B,c$; $A,B,\delta, c\mathbb{\in R}$;$R\subseteq\left\{ r\in\mathbb{R}^{+*} | r=n*k, n\mathbb{\in N,}k>0 \right\}$ or alternatively $R\subseteq\left\{ r\in\mathbb{R}^{-*} | r=-n*k, n\mathbb{\in N,}k>0 \right\}$; and $A \& B$ are isoreflective pair on a chosen pairing about a central point $\delta$.

Following Kabirian-based isomorphic optinalysis, we check the condition that $A$ is completely similar or identical to $B$. Then, we have

$${KC}_{Sim./Id.}\left( A,B \right)=\frac{4(x_{1}+x_{2}+x_{3}+\delta+x_{3}+ x_{2}+x_{1})}{x_{1}+2x_{2}+3x_{3}+4\delta+5x_{3}+6x_{2}+7x_{1}}$$

$${KC}_{Sim./Id.}\left( A,B \right)=\frac{8x_{1}+8x_{2}+8x_{3}+4\delta}{8x_{1}+8x_{2}+8x_{3}+4\delta}=1$$

Having satisfied the condition above, we transform by scaling as:

$${KC}_{Sim./Id.}\left( cA,cB \right)=\frac{4(cx_{1}+ cx_{2}+cx_{3}+\delta+cx_{3}+cx_{2}+cx_{1})}{cx_{1}+2cx_{2}+3cx_{3}+4\delta+5cx_{3}+6cx_{2}+7cx_{1}}$$

$${KC}_{Sim./Id.}\left( cA,cB \right)=\frac{8cx_{1}+8cx_{2}+8cx_{3}+4\delta}{8cx_{1}+8cx_{2}+8cx_{3}+4\delta}=1$$

We now conclude that ${KC}_{Sim./Id.}\left( A,B \right)={KC}_{Sim./Id.}\left( cA,cB \right)$. Therefore,

If ${KC}_{Sim./Id.}\left( A,B \right)=1$, then ${KC}_{Sim./Id.}\left( cA,cB \right)=1$

Similarly, the relationship is the same following the Kabirian-based optinalysis-to-probability translation models.

If $P_{Sim./Id.}\left( A,B \right)=1$, then $P_{Sim./Id.}\left( cA,cB \right)=1$

If $P_{Dsim./Uid.}\left( A,B \right)=0$, then $P_{Dsim./Uid.}\left( cA,cB \right)=0$

Let $A=(x_{1},x_{2}, x_{3},\ldots,x_{n})$ and its mirror image $A'=\left( {x'}_{1},{x'}_{'2}, {x'}_{3},\ldots,{x'}_{n} \right)$ be a mathematical structure. Following the Kabirian-based automorphic optinalysis and optinalysis-to-probability translation models, a similar conclusion will be drawn:

If ${KC}_{Sym./Id.}\left( A,A' \right)=1$, then ${KC}_{Sym./Id.}\left( cA,cA' \right)=1$

If $P_{Sym./Id.}\left( A,A' \right)=1$, then $P_{Sym./Id.}\left( cA,cA' \right)=1$

If $P_{Asym./Uid.}\left( A,A' \right)=0$, then $P_{Asym./Uid.}\left( cA,cA' \right)=0$

Therefore, Kabirian-based optinalysis is a scale-invariant.

*Prove of a theorem of transformation-invariance: central rotation-invariance*

Let the optinalytic construction of an isoreflective pair of mathematical structures $A$ and $B$ with an assigned optiscale ($R=1, 2, 3, 4, 5, 6, 7$) be:

$$f:\left[ \begin{matrix} A=(x_{1},x_{2},x_{3}) & \begin{matrix} \delta\\ ⇻ \end{matrix} & B=(x_{3}, x_{2},x_{1}) \\ ↡ & ↡ & ↡ \\ R= (1, 2, 3, & 4, & 5, 6, 7) \end{matrix} \right]$$

By central rotation, the optinalytic construction becomes:

$$f:\left[ \begin{matrix} B=(x_{1},x_{2},x_{3}) & \begin{matrix} \delta\\ ⇻ \end{matrix} & A=(x_{3}, x_{2},x_{1}) \\ ↡ & ↡ & ↡ \\ R= (1, 2, 3, & 4, & 5, 6, 7) \end{matrix} \right]$$

Such that $\delta\notin A,B$; $A,B,\delta\mathbb{\in R}$;$R\subseteq\left\{ r\in\mathbb{R}^{+*} | r=n*k, n\mathbb{\in N,}k>0 \right\}$ or alternatively $R\subseteq\left\{ r\in\mathbb{R}^{-*} | r=-n*k, n\mathbb{\in N,}k>0 \right\}$; and $A \& B$ are isoreflective pair on a chosen pairing about a central point $\delta$.

Following Kabirian-based isomorphic optinalysis, we check the condition that $A$ is completely similar or identical to $B$. Then, we have

$${KC}_{Sim./Id.}\left( A,B \right)=\frac{4(x_{1}+x_{2}+x_{3}+\delta+x_{3}+ x_{2}+x_{1})}{x_{1}+2x_{2}+3x_{3}+4\delta+5x_{3}+6x_{2}+7x_{1}}$$

$${KC}_{Sim./Id.}\left( A,B \right)=\frac{8x_{1}+8x_{2}+8x_{3}+4\delta}{8x_{1}+8x_{2}+8x_{3}+4\delta}=1$$

Having satisfied the condition above, we transform by central rotation (inversion) as:

$${KC}_{Sim./Id.}\left( B,A \right)=\frac{4(x_{1}+x_{2}+x_{3}+\delta+x_{3}+ x_{2}+x_{1})}{x_{1}+2x_{2}+3x_{3}+4\delta+5x_{3}+6x_{2}+7x_{1}}$$

$${KC}_{Sim./Id.}\left( B,A \right)=\frac{4x_{1}+4x_{2}+4x_{3}+4\delta+4x_{3}+4x_{2}+4x_{1}}{x_{1}+2x_{2}+3x_{3}+4\delta+5x_{3}+6x_{2}+7x_{1}}$$

$${KC}_{Sim./Id.}\left( B,A \right)=\frac{8x_{1}+8x_{2}+8x_{3}+4\delta}{8x_{1}+8x_{2}+8x_{3}+4\delta}=1$$

We now conclude that ${KC}_{Sim./Id.}\left( A,B \right)={KC}_{Sim./Id.}\left( B,A \right)$. Therefore,

If ${KC}_{Sim./Id.}\left( A,B \right)=1$, then ${KC}_{Sim./Id.}\left( B,A \right)=1$

Similarly, the relationship is the same following the Kabirian-based optinalysis-to-probability translation models.

If $P_{Sim./Id.}\left( A,B \right)=1$, then $P_{Sim./Id.}\left( B,A \right)=1$

If $P_{Dsim./Uid.}\left( A,B \right)=0$, then $P_{Dsim./Uid.}\left( B,A \right)=0$

Let $A=(x_{1},x_{2}, x_{3},\ldots,x_{n})$ and its mirror image $A'=\left( {x'}_{1},{x'}_{'2}, {x'}_{3},\ldots,{x'}_{n} \right)$ be a mathematical structure. Following the Kabirian-based automorphic optinalysis and optinalysis-to-probability translation models, a similar conclusion will be drawn:

If ${KC}_{Sym./Id.}\left( A,A' \right)=1$, then ${KC}_{Sym./Id.}\left( A',A \right)=1$

If $P_{Sym./Id.}\left( A,A' \right)=1$, then $P_{Sym./Id.}\left( A',A \right)=1$

If $P_{Asym./Uid.}\left( A,A' \right)=0$, then $P_{Asym./Uid.}\left( A',A \right)=0$

Therefore, Kabirian-based optinalysis is a central rotation-invariant.

*Prove of theorem of transformation-invariance: pericentral rotation-invariance*

Let the optinalytic construction of an isoreflective pair of mathematical structures $A$ and $B$ with an assigned optiscale ($R=1, 2, 3, 4, 5, 6, 7$) be:

$$f:\left[ \begin{matrix} A=(x_{1},x_{2},x_{3}) & \begin{matrix} \delta\\ ⇻ \end{matrix} & B=(x_{3}, x_{2},x_{1}) \\ ↡ & ↡ & ↡ \\ R= (1, 2, 3, & 4, & 5, 6, 7) \end{matrix} \right]$$

By alternate reflection, the optinalytic construction becomes:

$$f:\left[ \begin{matrix} A=(x_{3},x_{2},x_{1}) & \begin{matrix} \delta\\ ⇻ \end{matrix} & B=(x_{1}, x_{2},x_{3}) \\ ↡ & ↡ & ↡ \\ R= (1, 2, 3, & 4, & 5, 6, 7) \end{matrix} \right]$$

Such that $\delta\notin A,B$; $A,B,\delta\mathbb{\in R}$;$R\subseteq\left\{ r\in\mathbb{R}^{+*} | r=n*k, n\mathbb{\in N,}k>0 \right\}$ or alternatively $R\subseteq\left\{ r\in\mathbb{R}^{-*} | r=-n*k, n\mathbb{\in N,}k>0 \right\}$; and $A \& B$ are isoreflective pair in an annotated pairing about a central point $\delta$.

Following Kabirian-based isomorphic optinalysis, we check the condition that $A$ is completely similar or identical to $B$. Then, we have

$${KC}_{Sim./Id.}\left( A,\vec{B} \right)=\frac{4(x_{1}+x_{2}+x_{3}+\delta+x_{3}+ x_{2}+x_{1})}{x_{1}+2x_{2}+3x_{3}+4\delta+5x_{3}+6x_{2}+7x_{1}}$$

$${KC}_{Sim./Id.}\left( A,\vec{B} \right)=\frac{8x_{1}+8x_{2}+8x_{3}+4\delta}{8x_{1}+8x_{2}+8x_{3}+4\delta}=1$$

Having satisfied the condition above, we transform by pericentral rotation as:

$${KC}_{Sim./Id.}\left( \vec{A}, B \right)=\frac{4(x_{3}+x_{2}+x_{1}+\delta+x_{1}+ x_{2}+x_{3})}{x_{3}+2x_{2}+3x_{1}+4\delta+5x_{1}+6x_{2}+7x_{3}}$$

$${KC}_{Sim./Id.}\left( \vec{A}, B \right)=\frac{4x_{3}+4x_{2}+4x_{1}+4\delta+4x_{1}+4x_{2}+4x_{3}}{x_{3}+2x_{2}+3x_{1}+4\delta+5x_{1}+6x_{2}+7x_{3}}$$

$${KC}_{Sim./Id.}\left( \vec{A}, B \right)=\frac{8x_{3}+8x_{2}+8x_{1}+4\delta}{8x_{3}+8x_{2}+8x_{1}+4\delta}=1$$

We now conclude that ${KC}_{Sim./Id.}\left( A,\vec{B} \right)={KC}_{Sim./Id.}\left( \vec{A},B \right)$. Therefore,

If ${KC}_{Sim./Id.}\left( A,\vec{B} \right)=1$, then ${KC}_{Sim./Id.}\left( \vec{A},B \right)=1$

Similarly, the relationship is the same following the Kabirian-based optinalysis-to-probability translation models.

If $P_{Sim./Id.}\left( A,\vec{B} \right)=1$, then $P_{Sim./Id.}\left( \vec{A},B \right)=1$

If $P_{Dsim./Uid.}\left( A,\vec{B} \right)=0$, then $P_{Dsim./Uid.}\left( \vec{A},B \right)=0$

Let $A=(x_{1},x_{2}, x_{3},\ldots,x_{n})$ and its mirror image $A'=\left( {x'}_{1},{x'}_{'2}, {x'}_{3},\ldots,{x'}_{n} \right)$ be a mathematical structure. Following the Kabirian-based automorphic optinalysis and optinalysis-to-probability translation models, a similar conclusion will be drawn:

If ${KC}_{Sym./Id.}\left( A,\vec{A}' \right)=1$, then ${KC}_{Sym./Id.}\left( \vec{A},A' \right)=1$

If $P_{Sym./Id.}\left( A,\vec{A}' \right)=1$, then $P_{Sym./Id.}\left( \vec{A},A' \right)=1$

If $P_{Asym./Uid.}\left( A,\vec{A}' \right)=0$, then $P_{Asym./Uid.}\left( \vec{A},A' \right)=0$

Therefore, Kabirian-based optinalysis is a pericentral rotation-invariant.

*Prove of theorem of transformation-invariance: central modulation-invariance*

Let central modulation be $\delta+\beta$. Let the optinalytic construction of an isoreflective pair of mathematical structures $A$ and $B$ with an assigned optiscale ($R=1, 2, 3, 4, 5, 6, 7$) be:

$$f:\left[ \begin{matrix} A=(x_{1},x_{2},x_{3}) & \begin{matrix} \delta\\ ⇻ \end{matrix} & B=(x_{3}, x_{2},x_{1}) \\ ↡ & ↡ & ↡ \\ R= (1, 2, 3, & 4, & 5, 6, 7) \end{matrix} \right]$$

By central modulation, the optinalytic construction becomes:

$$f:\left[ \begin{matrix} A=(x_{1},x_{2},x_{3}) & \begin{matrix} \delta\pm\beta\\ ⇻ \end{matrix} & B=(x_{3}, x_{2},x_{1}) \\ ↡ & ↡ & ↡ \\ R= (1, 2, 3, & 4, & 5, 6, 7) \end{matrix} \right]$$

Such that $\delta\notin A,B$; $A, B, \delta, \beta\mathbb{\in R}$;$R\subseteq\left\{ r\in\mathbb{R}^{+*} | r=n*k, n\mathbb{\in N,}k>0 \right\}$ or alternatively $R\subseteq\left\{ r\in\mathbb{R}^{-*} | r=-n*k, n\mathbb{\in N,}k>0 \right\}$; and $A \& B$ are isoreflective pair on a chosen pairing about a central point $\delta$.

Following Kabirian-based isomorphic optinalysis, we check the condition that $A$ is completely similar or identical to $B$. Then, we have

$${KC}_{Sym./Id.}\left( A,B \right)=\frac{4(x_{1}+x_{2}+x_{3}+\delta+x_{3}+ x_{2}+x_{1})}{x_{1}+2x_{2}+3x_{3}+4\delta+5x_{3}+6x_{2}+7x_{1}}$$

$${KC}_{Sym./Id.}\left( A,B \right)=\frac{8x_{1}+8x_{2}+8x_{3}+4\delta}{8x_{1}+8x_{2}+8x_{3}+4\delta}=1$$

Having satisfied the condition above, we transform by central modulation as:

$${KC}_{Sym./Id.}\left( A,\delta\pm\beta,B \right)=\frac{4(x_{1}+x_{2}+x_{3}+(\delta\pm\beta)+x_{3}+ x_{2}+x_{1})}{x_{1}+2x_{2}+3x_{3}+4(\delta\pm\beta)+5x_{3}+6x_{2}+7x_{1}}$$

$${KC}_{Sym./Id.}\left( A,\delta\pm\beta,B \right)=\frac{8x_{1}+8x_{2}+8x_{3}+4(\delta\pm\beta)}{8x_{1}+8x_{2}+8x_{3}+4(\delta\pm\beta)}=1$$

We now conclude that ${KC}_{Sim./Id.}\left( A,B \right)={KC}_{Sim./Id.}\left( A,\delta\pm\beta,B \right)$. Therefore,

If ${KC}_{Sim./Id.}\left( A,B \right)=1$, then ${KC}_{Sim./Id.}\left( A,\delta\pm\beta,B \right)=1$

Similarly, the relationship is the same following the Kabirian-based optinalysis-to-probability translation models.

If $P_{Sim./Id.}\left( A,B \right)=1$, then $P_{Sim./Id.}\left( A,\delta\pm\beta,B \right)=1$

If $P_{Dsim./Uid.}\left( A,B \right)=0$, then $P_{Dsim./Uid.}\left( A,\delta\pm\beta,B \right)=0$

Let $A=(x_{1},x_{2}, x_{3},\ldots,x_{n})$ and its mirror image $A'=\left( {x'}_{1},{x'}_{'2}, {x'}_{3},\ldots,{x'}_{n} \right)$ be a mathematical structure. Following the Kabirian-based automorphic optinalysis and optinalysis-to-probability translation models, a similar conclusion will be drawn:

If ${KC}_{Sym./Id.}\left( A,A' \right)=1$, then ${KC}_{Sym./Id.}\left( A,\delta\pm\beta,A' \right)=1$

If $P_{Sym./Id.}\left( A,A' \right)=1$, then $P_{Sym./Id.}\left( A,\delta\pm\beta,A' \right)=1$

If $P_{Asym./Uid.}\left( A,A' \right)=0$, then $P_{Asym./Uid.}\left( A,\delta\pm\beta,A' \right)=0$

Therefore, Kabirian-based optinalysis is a central modulation-invariant.

**Appendix D: Transformation invariance of incompleteness**

Theorem: Let $A=(x_{1},x_{2}, x_{3},\ldots,x_{n})$ and its mirror image $A'=\left( {x'}_{1},{x'}_{'2}, {x'}_{3},\ldots,{x'}_{n} \right)$ be a mathematical structure. Let $A=(x_{1},x_{2}, x_{3},\ldots,x_{n})$ and $B=\left( x_{1},x_{2}, x_{3},\ldots,x_{n} \right)$ be two mathematical structures. If under Kabirian-based automorphic optinalysis ($Opt(A,A')$), or Kabirian-based isomorphic optinalysis ($Opt(A,B)$); and $A$ and $A'$ are incompletely symmetrical/identical, or $A$ and $B$ are incompletely similar/identical; then the estimates are invariant to transformations such as product translation (scaling) and central rotation (inversion).

*Prove of a theorem of transformation-invariance: scale-invariance*

Let the optinalytic construction of an isoreflective pair of mathematical structures $A$ and $B$ with an assigned optiscale ($R=1, 2, 3, 4, 5, 6, 7$) be:

$$f:\left[ \begin{matrix} A=(tx_{1},x_{2},x_{3}) & \begin{matrix} \delta\\ ⇻ \end{matrix} & B=(x_{3}, x_{2},x_{1}) \\ ↡ & ↡ & ↡ \\ R= (1, 2, 3, & 4, & 5, 6, 7) \end{matrix} \right]$$

Let $c$ be a scale factor. The optinalytic construction becomes:

$$f:\left[ \begin{matrix} A=(ctx_{1}, cx_{2},cx_{3}) & \begin{matrix} \delta\\ ⇻ \end{matrix} & B=(cx_{3},cx_{2},cx_{1}) \\ ↡ & ↡ & ↡ \\ R= (1, 2, 3, & 4, & 5, 6, 7) \end{matrix} \right]$$

Such that $\delta\notin A, B,c$; $A,B,\delta, R,c\mathbb{\in R}$;$R\subseteq\left\{ r\in\mathbb{R}^{+*} | r=n*k, n\mathbb{\in N,}k>0 \right\}$ or alternatively $R\subseteq\left\{ r\in\mathbb{R}^{-*} | r=-n*k, n\mathbb{\in N,}k>0 \right\}$; and $A \& B$ are isoreflective pair on a chosen pairing about a central point $\delta$.

Following Kabirian-based isomorphic optinalysis, we check the condition that $A$ is incompletely similar or identical to $B$. Then, we have

$${KC}_{Sim./Id.}\left( A,B \right)=\frac{4({tx}_{1}+x_{2}+x_{3}+\delta+x_{3}+ x_{2}+x_{1})}{tx_{1}+2x_{2}+3x_{3}+4\delta+5x_{3}+6x_{2}+7x_{1}}$$

$${KC}_{Sim./Id.}\left( A,B \right)=\frac{4tx_{1}+4x_{1}+8x_{2}+8x_{3}+4\delta}{tx_{1}+7x_{1}+8x_{2}+8x_{3}+4\delta}\neq1$$

Having satisfied the condition above, we transform by scaling as:

$${KC}_{Sim./Id.}\left( cA,cB \right)=\frac{4(ctx_{1}+ cx_{2}+cx_{3}+\delta+cx_{3}+cx_{2}+cx_{1})}{c{tx}_{1}+2cx_{2}+3cx_{3}+4\delta+5cx_{3}+6cx_{2}+7cx_{1}}$$

$${KC}_{Sim./Id.}\left( cA,cB \right)=\frac{c(4tx_{1}+4x_{1}+8x_{2}+8x_{3})+4\delta}{c(tx_{1}+7x_{1}+8x_{2}+8x_{3})+4\delta}=\frac{4tx_{1}+4x_{1}+8x_{2}+8x_{3}+4\delta}{tx_{1}+7x_{1}+8x_{2}+8x_{3}+4\delta}\neq1$$

We now conclude that ${KC}_{Sim./Id.}\left( A,B \right)={KC}_{Sim./Id.}\left( cA,cB \right)$. Therefore,

If ${KC}_{Sim./Id.}\left( A,B \right)\neq1$, then ${KC}_{Sim./Id.}\left( cA,cB \right)={KC}_{Sim./Id.}\left( A,B \right)$

Similarly, the relationship is the same following the Kabirian-based optinalysis-to-probability translation models.

If$P_{Sim./Id.}\left( A,B \right)\neq1$, then $P_{Sim./Id.}\left( cA,cB \right)=P_{Sim./Id.}\left( A,B \right)$

If $P_{Dsim./Uid.}\left( A,B \right)\neq1$, then $P_{Dsim./Uid.}\left( cA,cB \right)=P_{Dsim./Uid.}\left( A,B \right)$

Let $A=(x_{1},x_{2}, x_{3},\ldots,x_{n})$ and its mirror image $A'=\left( {x'}_{1},{x'}_{'2}, {x'}_{3},\ldots,{x'}_{n} \right)$ be a mathematical structure. Following the Kabirian-based automorphic optinalysis and optinalysis-to-probability translation models, a similar conclusion will be drawn:

If ${KC}_{Sym./Id.}\left( A,A' \right)\neq1$, then ${KC}_{Sym./Id.}\left( cA,cA' \right)={KC}_{Sym./Id.}\left( A,A' \right)$

If$P_{Sym./Id.}\left( A,A' \right)\neq1$, then $P_{Sym./Id.}\left( cA,cA' \right)=P_{Sym./Id.}\left( A,A' \right)$

If $P_{Asym./Uid.}\left( A,A' \right)\neq1$, then $P_{Asym./Uid.}\left( cA,cA' \right)=P_{Asym./Uid.}\left( A,A' \right)$

Therefore, Kabirian-based optinalysis is a scale-invariant.

*Prove of a theorem of transformation-invariance: central rotation-invariance*

Let the optinalytic construction of an isoreflective pair of mathematical structures $A$ and $B$ with an assigned optiscale ($R=1, 2, 3, 4, 5, 6, 7$) be:

$$f:\left[ \begin{matrix} A=(tx_{1},x_{2},x_{3}) & \begin{matrix} \delta\\ ⇻ \end{matrix} & B=(x_{3}, x_{2},x_{1}) \\ ↡ & ↡ & ↡ \\ R= (1, 2, 3, & 4, & 5, 6, 7) \end{matrix} \right]$$

By central rotation, the optinalytic construction becomes:

$$f:\left[ \begin{matrix} B=(x_{1},x_{2},x_{3}) & \begin{matrix} \delta\\ ⇻ \end{matrix} & A=(x_{3}, x_{2},tx_{1}) \\ ↡ & ↡ & ↡ \\ R= (1, 2, 3, & 4, & 5, 6, 7) \end{matrix} \right]$$

Such that $\delta\notin A, B$; $A,B,\delta\mathbb{\in R}$;$R\subseteq\left\{ r\in\mathbb{R}^{+*} | r=n*k, n\mathbb{\in N,}k>0 \right\}$ or alternatively $R\subseteq\left\{ r\in\mathbb{R}^{-*} | r=-n*k, n\mathbb{\in N,}k>0 \right\}$; and $A \& B$ are isoreflective pair on a chosen pairing about a central point $\delta$.

Following Kabirian-based isomorphic optinalysis, we check the condition that $A$ is incompletely similar or identical to $B$. Then, we have

$${KC}_{Sim./Id.}\left( A,B \right)=\frac{4({tx}_{1}+x_{2}+x_{3}+\delta+x_{3}+ x_{2}+x_{1})}{tx_{1}+2x_{2}+3x_{3}+4\delta+5x_{3}+6x_{2}+7x_{1}}$$

$${KC}_{Sim./Id.}\left( A,B \right)=\frac{4tx_{1}+4x_{1}+8x_{2}+8x_{3}+4\delta}{tx_{1}+7x_{1}+8x_{2}+8x_{3}+4\delta}\neq1$$

Having satisfied the condition above, we transform by central rotation as:

$${KC}_{Sim./Id.}\left( B,A \right)=\frac{4(x_{1}+x_{2}+x_{3}+\delta+x_{3}+ x_{2}+tx_{1})}{tx_{1}+2x_{2}+3x_{3}+4\delta+5x_{3}+6x_{2}+7x_{1}}$$

$${KC}_{Sim./Id.}\left( B,A \right)=\frac{4x_{1}+4x_{2}+4x_{3}+4\delta+4x_{3}+4x_{2}+4tx_{1}}{x_{1}+2x_{2}+3x_{3}+4\delta+5x_{3}+6x_{2}+7tx_{1}}=\frac{4x_{1}+4tx_{1}+8x_{2}+8x_{3}+4\delta}{x_{1}+7tx_{1}+8x_{2}+8x_{3}+4\delta}\neq1$$

We now conclude that ${KC}_{Sim./Id.}\left( A,B \right)\neq{KC}_{Sim./Id.}\left( B,A \right)$. Therefore,

If ${KC}_{Sim./Id.}\left( A,B \right)\neq1$, then ${KC}_{Sim./Id.}\left( B,A \right)\neq{KC}_{Sim./Id.}\left( A,B \right)$

Similarly, the relationship is the same following the Kabirian-based optinalysis-to-probability translation models.

If$P_{Sim./Id.}\left( A,B \right)\neq1$, then $P_{Sim./Id.}\left( B,A \right)=P_{Sim./Id.}\left( A,B \right)$

If $P_{Dsim./Uid.}\left( A,B \right)\neq1$, then $P_{Dsim./Uid.}\left( B,A \right)=P_{Dsim./Uid.}\left( A,B \right)$

Let $A=(x_{1},x_{2}, x_{3},\ldots,x_{n})$ and its mirror image $A'=\left( {x'}_{1},{x'}_{'2}, {x'}_{3},\ldots,{x'}_{n} \right)$ be a mathematical structure. Following the Kabirian-based automorphic optinalysis and optinalysis-to-probability translation models, a similar conclusion will be drawn:

If ${KC}_{Sym./Id.}\left( A,A' \right)\neq1$, then ${KC}_{Sym./Id.}\left( A',A \right)\neq{KC}_{Sym./Id.}\left( A,A' \right)$

If$P_{Sym./Id.}\left( A,A' \right)\neq1$, then $P_{Sym./Id.}\left( A',A \right)=P_{Sym./Id.}\left( A,A' \right)$

If $P_{Asym./Uid.}\left( A,A' \right)\neq1$, then $P_{Asym./Uid.}\left( A',A \right)=P_{Asym./Uid.}\left( A,A' \right)$

Therefore, Kabirian-based optinalysis is a central rotation-invariant.

**Appendix E: Optinalytic normalization**

Theorem: Let $A=(x_{1},x_{2}, x_{3},\ldots,x_{n})$ and its mirror image $A'=\left( {x'}_{1},{x'}_{'2}, {x'}_{3},\ldots,{x'}_{n} \right)$ be a mathematical structure. Let $A=(x_{1},x_{2}, x_{3},\ldots,x_{n})$ and $B=\left( x_{1},x_{2}, x_{3},\ldots,x_{n} \right)$ be two mathematical structures. Let central modulation be $\delta+\beta$. If under Kabirian-based automorphic optinalysis ($Opt(A,A')$), or Kabirian-based isomorphic optinalysis ($Opt(A,B)$); and $A$ and $A'$ are incompletely symmetrical/identical, or $A$ and $B$ are incompletely similar/identical; then the estimates of the optinalysis can be neutralized to near-completeness through central modulation at the central point that connected the mathematical structure(s).

*Prove the theorem of optinalytic normalization*

Let the optinalytic construction of an isoreflective pair of mathematical structures $A$ and $B$ with an assigned optiscale ($R=1, 2, 3, 4, 5, 6, 7$) be:

$$f:\left[ \begin{matrix} A=(tx_{1},x_{2},x_{3}) & \begin{matrix} \delta\\ ⇻ \end{matrix} & B=(x_{3}, x_{2},x_{1}) \\ ↡ & ↡ & ↡ \\ R= (1, 2, 3, & 4, & 5, 6, 7) \end{matrix} \right]$$

By central modulation, the optinalytic construction becomes:

$$f:\left[ \begin{matrix} A=({tx}_{1},x_{2},x_{3}) & \begin{matrix} \delta\pm\beta\\ ⇻ \end{matrix} & B=(x_{3}, x_{2},x_{1}) \\ ↡ & ↡ & ↡ \\ R= (1, 2, 3, & 4, & 5, 6, 7) \end{matrix} \right]$$

Such that $\delta\notin A, B$; $A,B,\delta,\beta\mathbb{\in R}$;$R\subseteq\left\{ r\in\mathbb{R}^{+*} | r=n*k, n\mathbb{\in N,}k>0 \right\}$ or alternatively $R\subseteq\left\{ r\in\mathbb{R}^{-*} | r=-n*k, n\mathbb{\in N,}k>0 \right\}$; and $A \& B$ are isoreflective pair on a chosen pairing about a central point $\delta$.

Following Kabirian-based isomorphic optinalysis, we check the condition that $A$ is incompletely similar or identical to $B$. Then, we have

$${KC}_{Sim./Id.}\left( A,B \right)=\frac{4({tx}_{1}+x_{2}+x_{3}+\delta+x_{3}+ x_{2}+x_{1})}{tx_{1}+2x_{2}+3x_{3}+4\delta+5x_{3}+6x_{2}+7x_{1}}$$

$${KC}_{Sim./Id.}\left( A,B \right)=\frac{4tx_{1}+4x_{1}+8x_{2}+8x_{3}+4\delta}{tx_{1}+7x_{1}+8x_{2}+8x_{3}+4\delta}\neq1$$

Having satisfied the condition above, we transform by central modulation as:

$${KC}_{Sim./Id.}\left( A,\delta\pm\beta,B \right)=\frac{4(tx_{1}+x_{2}+x_{3}+(\delta\pm\beta)+x_{3}+ x_{2}+x_{1})}{{tx}_{1}+2x_{2}+3x_{3}+4(\delta\pm\beta)+5x_{3}+6x_{2}+7x_{1}}$$

$${KC}_{Sim./Id.}\left( A,\delta\pm\beta,B \right)=\frac{4{tx}_{1}+4x_{2}+4x_{3}+4(\delta\pm\beta)+4x_{3}+4x_{2}+4x_{1}}{tx_{1}+2x_{2}+3x_{3}+4(\delta\pm\beta)+5x_{3}+6x_{2}+7x_{1}}$$

$${KC}_{Sim./Id.}\left( A,\delta\pm\beta,B \right)=\frac{4tx_{1}+4x_{1}+8x_{2}+8x_{3}+4(\delta\pm\beta)}{tx_{1}+7x_{1}+8x_{2}+8x_{3}+4(\delta\pm\beta)}\approx1$$

We now conclude that $\lim_{\beta\to\infty} {KC}_{Sim./Id.}\left( A,\delta\pm\beta,B \right)\approx1$. Therefore,

If ${KC}_{Sim./Id.}\left( A,\delta,B \right)\neq1$, then $\lim_{\beta\to\infty} {KC}_{Sim./Id.}\left( A,\delta\pm\beta,B \right)\approx1$.

Similarly, the relationship is the same following the Kabirian-based optinalysis-to-probability translation models.

If $P_{Sim./Id.}\left( A,\delta,B \right)\neq1$, then $\lim_{\beta\to\infty} P_{Sim./Id.}\left( A,\delta\pm\beta,B \right)\approx1$.

If $P_{Dsim./Uid.}\left( A,\delta,B \right)\neq1$, then $\lim_{\beta\to\infty} P_{Dsim./Uid.}\left( A,\delta\pm\beta,B \right)\approx1$.

Let $A=(x_{1},x_{2}, x_{3},\ldots,x_{n})$ and its mirror image $A'=\left( {x'}_{1},{x'}_{'2}, {x'}_{3},\ldots,{x'}_{n} \right)$ be a mathematical structure. Following the Kabirian-based automorphic optinalysis and optinalysis-to-probability translation models, a similar conclusion will be drawn:

If ${KC}_{Sym./Id.}\left( A,\delta,A' \right)\neq1$, then $\lim_{\beta\to\infty} {KC}_{Sym./Id.}\left( A,\delta\pm\beta,A' \right)\approx1$.

If $P_{Sym./Id.}\left( A,\delta,A' \right)\neq1$, then $\lim_{\beta\to\infty} P_{Sym./Id.}\left( A,\delta\pm\beta,A' \right)\approx1$.

If $P_{Asym./Uid.}\left( A,\delta,A' \right)\neq1$, then $\lim_{\beta\to\infty} P_{Asym./Uid.}\left( A,\delta\pm\beta,A' \right)\approx1$.

Therefore, Kabirian-based optinalysis is sensitive to central modulation under incompleteness.
